# Influenza A Virus Migration and Persistence in North American Wild Birds

**DOI:** 10.1371/journal.ppat.1003570

**Published:** 2013-08-29

**Authors:** Justin Bahl, Scott Krauss, Denise Kühnert, Mathieu Fourment, Garnet Raven, S. Paul Pryor, Lawrence J. Niles, Angela Danner, David Walker, Ian H. Mendenhall, Yvonne C. F. Su, Vivien G. Dugan, Rebecca A. Halpin, Timothy B. Stockwell, Richard J. Webby, David E. Wentworth, Alexei J. Drummond, Gavin J. D. Smith, Robert G. Webster

**Affiliations:** 1 Laboratory of Virus Evolution, Program in Emerging Infectious Diseases, Duke-NUS Graduate Medical School, Singapore; 2 Center for Infectious Diseases, The University of Texas School of Public Health, Houston, Texas, United States of America; 3 Department of Infectious Diseases, St. Jude Children's Research Hospital, Memphis, Tennessee, United States of America; 4 Department of Computer Science, University of Auckland, Auckland, New Zealand; 5 Allan Wilson Centre for Molecular Ecology and Evolution, University of Auckland, Auckland, New Zealand; 6 Environment Canada, Canadian Wildlife Service, Edmonton, Alberta, Canada; 7 Conserve Wildlife Foundation of New Jersey, Bordentown, New Jersey, United States of America; 8 J. Craig Venter Institute, Rockville, Maryland, United States of America; 9 Division of Microbiology and Infectious Diseases/National Institute of Allergy and Infectious Diseases/National Institutes of Health/Department of Health and Human Services, Bethesda, Maryland, United States of America; 10 Duke Global Health Institute, Duke University, Durham, North Carolina, United States of America; University of California San Francisco, United States of America

## Abstract

Wild birds have been implicated in the emergence of human and livestock influenza. The successful prediction of viral spread and disease emergence, as well as formulation of preparedness plans have been hampered by a critical lack of knowledge of viral movements between different host populations. The patterns of viral spread and subsequent risk posed by wild bird viruses therefore remain unpredictable. Here we analyze genomic data, including 287 newly sequenced avian influenza A virus (AIV) samples isolated over a 34-year period of continuous systematic surveillance of North American migratory birds. We use a Bayesian statistical framework to test hypotheses of viral migration, population structure and patterns of genetic reassortment. Our results reveal that despite the high prevalence of *Charadriiformes* infected in Delaware Bay this host population does not appear to significantly contribute to the North American AIV diversity sampled in *Anseriformes*. In contrast, influenza viruses sampled from *Anseriformes* in Alberta are representative of the AIV diversity circulating in North American *Anseriformes*. While AIV may be restricted to specific migratory flyways over short time frames, our large-scale analysis showed that the long-term persistence of AIV was independent of bird flyways with migration between populations throughout North America. Analysis of long-term surveillance data provides vital insights to develop appropriately informed predictive models critical for pandemic preparedness and livestock protection.

## Author Summary

Despite continuous virological surveillance (1976–2009) in wild waterfowl (*Anseriformes*) and shorebirds (*Charadriiformes*), the ecological and evolutionary dynamics of avian influenza A virus (AIV) in these hosts is poorly understood. Comparative genomic analysis of AIV data revealed that the high prevalence of *Charadriiformes* infected in Delaware Bay is a reservoir of AIV that is phylogenetically distinct from AIV sampled from most North American *Anseriformes*. In contrast, influenza viruses sampled from *Anseriformes* in Alberta are representative of the remaining AIV diversity sampled across North America. While AIV may be restricted to specific migratory flyways over short time frames, our large-scale analysis showed that this population genetic structure was transient and the long-term persistence of AIV was independent of bird flyways. These results suggest an introduced virus lineage may initially be restricted to one flyway, but migration to a major congregation site such as Alberta could occur followed by subsequent spread across flyways. These generalized predictions for virus movement will be critical to assess the associated risk for widespread diffusion and inform surveillance for pandemic preparedness.

## Introduction

Migrating wild birds have been implicated in the spread and emergence of human and livestock influenza, including pandemic influenza and highly pathogenic H5N1 avian influenza [1]–[3]. Viral transmission between wild birds and domestic poultry has contributed to genomic reassortment and confounded disease control efforts [2], [4]. Subsequently, with the reintroduction of H5N1 to wild birds the virus has spread throughout Eurasia and Africa [5]–[9]. While it is contentious as to whether wild birds are the primary vectors spreading H5N1 viruses over long distances, there is little doubt that these animals play a role in confounding disease surveillance and control efforts.

It is estimated worldwide that over 50 billion birds migrate annually between breeding and non-breeding areas [10]. Even though there is evidence that *Anseriformes* infected with influenza A virus have hampered migration, these hosts vector influenza viruses vast distances [11]–[12]. Disease transmissions between the millions of conspecific birds at congregating sites throughout the world contribute to the genetic variability and reassortment of influenza A viruses [13], [14]. It is not coincidental that these major breeding, feeding, and staging sites are also regions of high viral prevalence [14]–[21].

Recent efforts to assess invasive virological threats have focused on increased surveillance and early detection of introduced viral strains [22]–[24]. Influenza A viruses have transmitted between the Eurasian and North American wild *Anseriformes* and *Charadriformes* gene pools where birds from both continental regions commingle and therefore the threat posed by introduction of H5N1 to North America remains. However, once a virological threat has entered the North American bird population there is little information regarding how that virus may behave or diffuse between spatially distant migratory bird populations.

The prediction of viral spread and disease emergence, as well as formulation of preparedness plans has generally been based on *ad hoc* approaches. This is largely due to a critical lack of knowledge of viral movements between different host populations [13]–[17]. The patterns of viral spread and subsequent risk posed by wild bird viruses therefore remain unpredictable. Methodological advances present an opportunity for large-scale assessment of spatiotemporal patterns of viral movement between migrating bird populations.

In this study we identified 20 discrete regions in North America where influenza viruses have been systematically collected from wild birds to determine whether the viral population was structured according to host migratory flyways, and rates of gene flow between these populations. Avian influenza viruses were isolated annually throughout our surveillance in Alberta, Canada and Delaware Bay, USA and an additional 287 genomes were sequenced. Using full genome data we characterize the reassortment dynamics, spatial diffusion patterns and evolutionary genomics of influenza A viruses in North America collected over a 25-year period from migratory birds.

## Results

Avian influenza H3 viruses were among the most frequently isolated influenza subtype from our surveillance in Alberta, Canada and Delaware Bay, USA [17]. We therefore randomly selected 200 H3 subtype isolates collected from 1976 to 2009 – plus an additional 100 influenza isolates of multiple subtypes – for full genome sequencing. Thirteen isolates could not be sequenced and a number of additional isolates were mixed samples containing multiple subtypes. As a result, 163 H3 subtype viruses and 124 isolates of other subtypes were sequenced. The newly sequenced H3-HA genes were analyzed with publically available H3-HA data to estimate the phylogenetic history (number of taxa (ntax) = 531). This large scale phylogeny of globally sampled H3 viruses from wild birds revealed three major lineages, two circulating in North America (Lineages I and II) and a third lineage that is a mix of North American and Eurasian isolates (Figure S1). All gene sequences that were of Eurasian origin were excluded from all further analysis in this study, including those that belonged to the mixed Eurasian/North American lineage.

**Figure 1:**
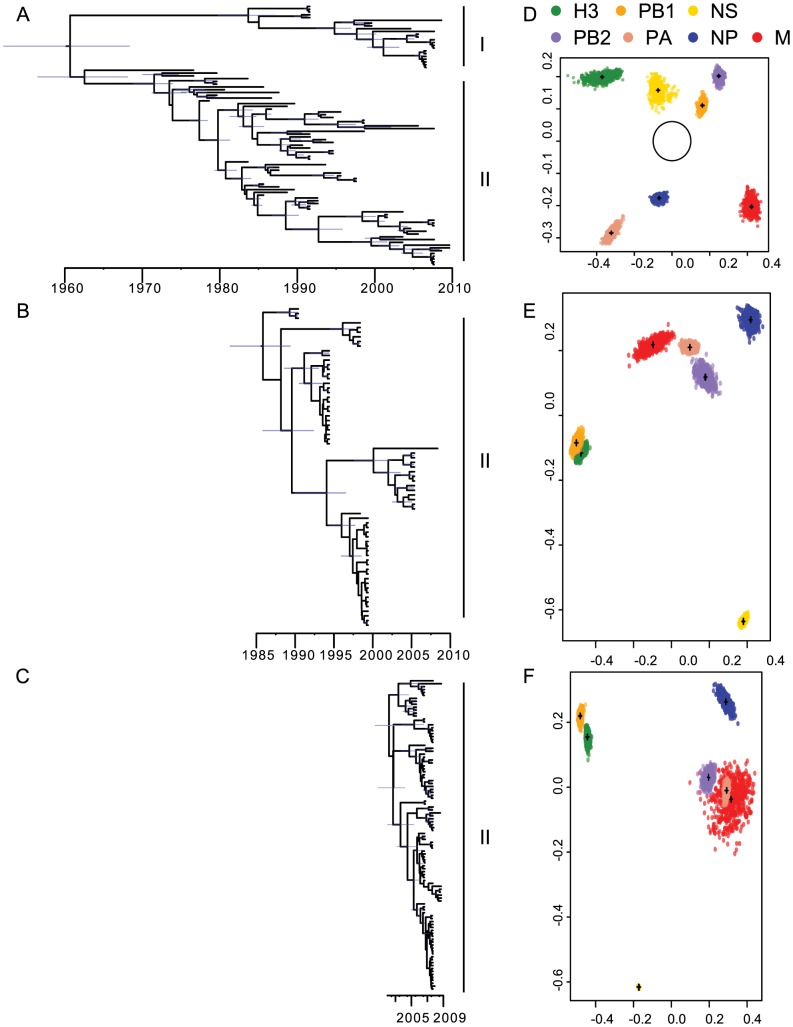
A) H3-HA phylogenetic tree for isolates from Alberta. **B) H3-HA phylogenetic tree for isolates from Delaware Bay.** C) H3-HA phylogenetic tree for isolates from Alaska. D) Multidimensional scaling of tree-to-tree TMRCA estimates from Alberta. For reference, the space occupied by human H3N2 viruses from similar analysis is centered (grey circle). E) Multidimensional scaling of tree-to-tree patristic distance from Delaware Bay. F) Multidimensional scaling of tree-to-tree patristic distance from Alaska.

Comparative genomic analysis of H3 subtype viruses isolated from the Alberta and Delaware Bay sites was conducted to test AIV evolutionary dynamics in different hosts. In Alberta, where birds sampled were primarily juvenile *Anseriformes* [20] the H3-HA phylogeny showed that H3 viruses were recovered in almost every year (ntax = 94), with both Lineage I and II viruses present (Figure 1A). In contrast, in Delaware Bay, where only *Charadriiformes* were sampled, H3 viruses were detected in only 7 years (ntax = 69) from 24 years of surveillance (Figure 1B). In those years when H3 viruses were isolated in Delaware Bay, only a single clade was detected each sampling season and no co-circulation of these clades was apparent. While viral prevalence in Delaware Bay and Alberta are similar [17], *Anseriformes* host a representative diversity of AIV in North America. In contrast, *Charadriiformes* host limited viral diversity exhibiting local epidemic-like dynamics [25] suggesting *Charadriformes* in Delaware Bay are being infected from a currently undetected AIV population.

We used multidimensional scaling of times of most recent common ancestor (tMRCAs) and patristic distances for each gene segment (excluding NA) to test differences in reassortment between populations (Figure 1C, D). In this analysis, the spread of each point cloud represents the statistical uncertainty in the phylogenetic history of each gene and we expect non-reassortant genes will have overlapping point clouds [26]. For both Alberta and Delaware Bay these analyses clearly indicate high levels of reassortment and that the evolutionary histories of the HA and internal genes are therefore partially independent, although the HA and PB1 from Delaware Bay show a higher level of similarity.

**Figure 2:**
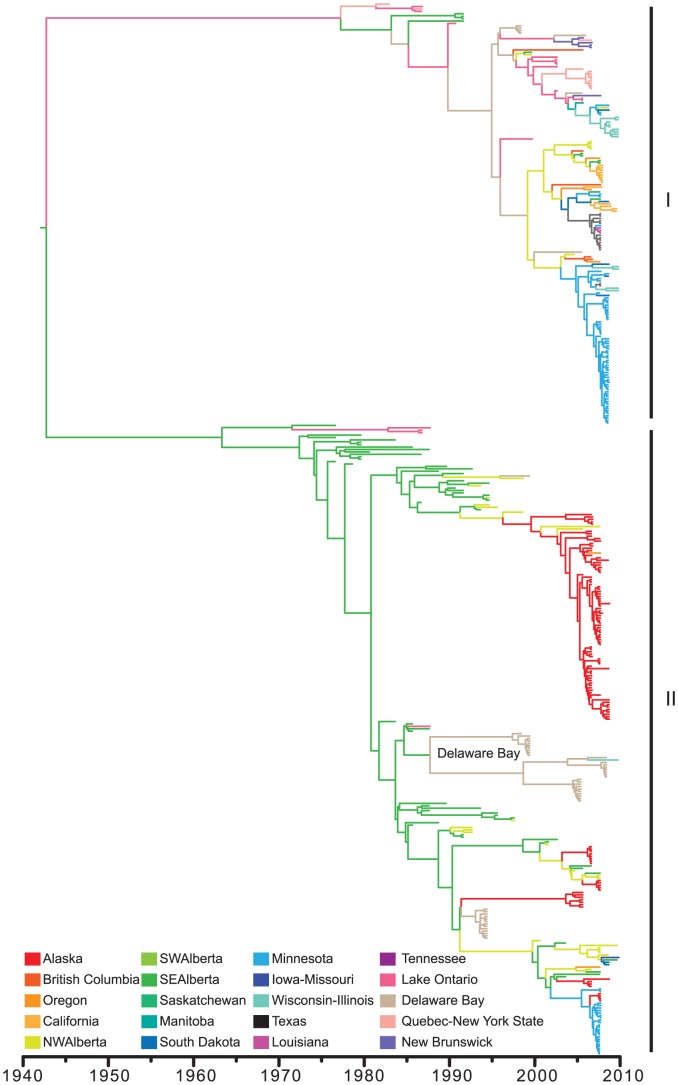
Bayesian relaxed clock HA gene phylogenetic tree from all H3 wild bird isolates in North America. The two co-circulating North American lineages (I and II) are annotated to the right of the tree. Branches are colored according to ancestral state location estimated from geographical tip-state observations for all observed localities.

To evaluate evolutionary dynamics and migration patterns of H3 subtype viruses throughout North America we identified viruses from avian hosts sampled in 20 defined discrete geographic regions excluding those sequences with recently introduced from Eurasia as described above (ntax = 437). The tMRCA of Lineages I and II was estimated to be ∼1942 (95% Bayesian Credibility Interval 1926–1962). The mechanism for maintenance of this deep divergence remains unknown, as viruses from both lineages have co-circulated in geographically overlapping host populations, primarily *Anseriformes*, throughout the entire surveillance period. One possibility is that this deep divergence is the product of (i) a very large host meta-population and (ii) relatively rare cross-species transmission rate when compared to annual seasonal epidemic dynamics leading to a lack of synchronicity of partial immunity across host species so that more than one lineage can effectively survive long periods of time. Although there was little evidence for geographic structuring of the virus population over extended periods, an obvious exception is a single lineage that has circulated for more than 10 years in birds sampled from Delaware Bay (Figure 2).

Ancestral state reconstruction of virus geographic location suggests that the population of Lineage II was localized in southeast Alberta prior to migrating to other locations across all North American flyways (Figure 2). However, the apparent geographic isolation of viruses from Alberta may be an artifact as sampling in this location began 12 years before other sites. Furthermore, in Lineage I, where sampling was temporally and spatially more consistent, we found no evidence of localized ancestral populations.

We next estimated rates of viral migration between discrete geographic locations treating each gene as an independent dataset to capitalize on the extra historical information generated by genetic reassortment. While each gene segment analysed supported lateral diffusion between migratory flyways over time, analysis of migration paths using single gene segments yielded contradictory answers (Figure S2, S3, S4, S5, S6, S7, S8). For example, the PB1 gene analysis highly supported migration events within the Pacific flyways, although none of the other gene segment analyses did (Figure S4). This is probably a reflection of the high rates of reassortment unlinking the evolutionary history of individual gene segments between subtypes.

**Figure 3:**
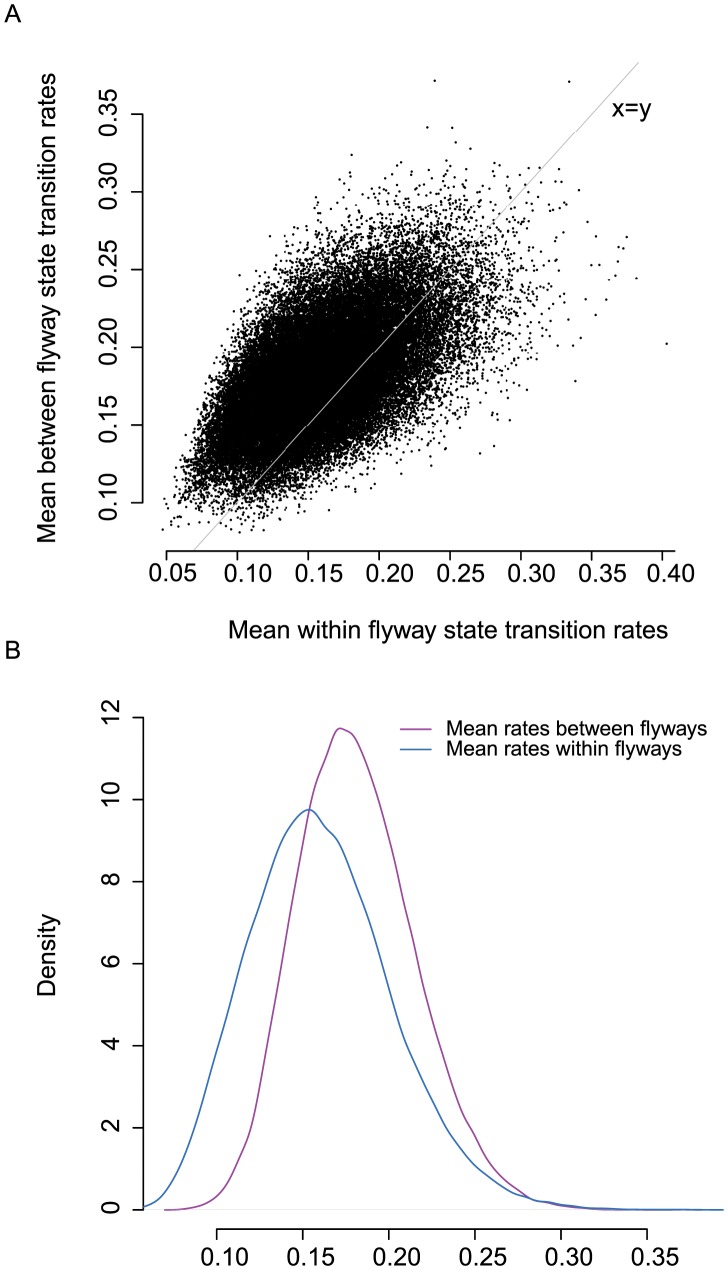
A) Mean migration rate per MCMC step within flyway migration rates vs Mean between flyway migration jointly estimated from all publically available PA, PB1, PB2, NP and M gene segments. B) Density distribution of mean within flyway and mean between flyway rates.

We further analyzed all publically available PA, PB1, PB2, NP and M sequence data from wild aquatic birds isolated between 1985–2009 in North America. The HA, NA and NS gene segments were not included in this analysis due to the deep divergence between the subtypes [16]. In this analysis we defined 16 geographic states and a 17^th^ state termed “Other”, that maintained phylogenetic tree structure. The “Other” state included taxa isolated prior to 1998 where few geographic locations were sampled and locations where few isolates were encountered over the surveillance period [27]. This analysis included more than 1300 sequences for each gene. The migration pattern was jointly estimated from all gene datasets in a single analysis even though the taxon number and subtype between each gene dataset was not identical. The phylogenetic tree space was sampled independently for each dataset, but we assumed the migration parameters were linked. These parameters were estimated across all gene trees to elucidate the migration history of the avian influenza population in North American wild birds and showed similar levels of within versus between flyway migration rates (Figure 3). This was confirmed by statistical comparison of these rates, which showed no significant difference in diffusion patterns (mean within flyway rate>mean between flyway rate, Bayes factor (BF) = 0.968; mean between flyway rate>mean within flyway rate, BF = 1.033).

**Figure 4:**
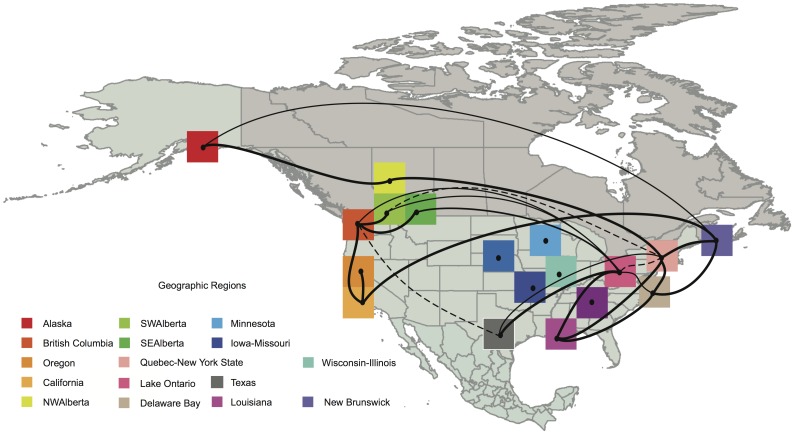
Patterns of viral migration jointly estimated across the 5 internal protein gene segments. Lines connecting discrete regions indicate statistically supported ancestral state changes and are thickened according to statistical support. There are five categories of support. The thinnest lines indicate 6≤BF<10 (supported); 10≤BF<30 (strong support); 30≤BF<100 (very strong support) and the thickest lines with BF≤100 (decisive support). Dashed lines indicate statistical supports between 3≤BF<6 but with posterior probabilities <0.5.

Table 1 shows the mean migration rates for all statistically supported state transitions recovered from our analysis. The diffusion patterns recovered from this analysis show that when all subtypes, hosts and locations are considered there is extensive mixing of influenza A virus between populations (Figure 4). However, it is unlikely that this pattern can be generalized for individual subtypes. For example, analysis of H3-HA gene segments with the six other internal gene segments (excluding NA) showed greater within flyway migration compared to between flyway migration (Figure S2, S3, S4, S5, S6, S7, S8, S10). Surprisingly, we could not reject the null hypothesis that migration rates are unrelated to the distance between locations (Pearson correlation coefficient = −0.037; Mantel test of rates vs distance, p = 0.317, Figure S10). However, the large-scale spatial diffusion and persistence of AIV is facilitated by comingling of birds in congregation sites located where multiple flyways overlap, such as Alberta (Figure 4). Taken together these results suggest that the AIV population mixes extensively and rapidly despite large geographic separation between sampling locations.

**Table 1:** Statistically supported state transitions indicating migratory events.

Transition Between ψ		Distance in km	Median Rate	Mean Rate	Mean indicator†	Bayes Factor*
Ontario-Ohio	Texas	1991	7.46	7.64	1	>100
Alaska	NW Alberta	1619	2.57	2.68	1	>100
New Brunswick	Delaware Bay	1359	1.44	1.51	1	>100
British Columbia	SE Alberta	713	1.37	1.41	1	>100
Ontario-Ohio	Delaware Bay	715	1.19	1.24	0.77	29
Alaska	New Brunswick	4797	1.01	1.11	0.6	13
Oregon	California	556	0.93	0.97	1	>100
Quebec-NY State	Texas	2665	0.87	0.87	0.61	13
SE Alberta	Ontario-Ohio	2514	0.77	0.8	0.85	47
British Columbia	Ontario-Ohio	3141	0.69	0.71	0.52	10
British Columbia	California	1390	0.63	0.65	1	>100
Quebec-NY State	Mississippi-Louisiana	2009	0.63	0.64	1	>100
Quebec-NY State	Delaware Bay	858	0.57	0.59	1	>100
Ontario-Ohio	Mississippi-Louisiana	1373	0.49	0.51	0.99	>100
Delaware Bay	Mississippi-Louisiana	1432	0.4	0.42	1	>100
NW Alberta	Quebec-NY State	3188	0.39	0.4	1	>100
Quebec-NY State	New Brunswick	616	0.25	0.26	1	>100
British Columbia	SW Alberta	749	0.18	0.19	1	>100
California	Quebec-NY State	4029	0.13	0.14	1	>100
SW Alberta	Ontario-Ohio	2447	0.12	0.13	0.74	25

ψ State Transition between the “Other” and Texas was supported once in our analysis (BF = 64, I = 88) likely due to the broad taxonomic sampling included in the “Other” state and phylogenetic uncertainty in estimating migration.

† The indicator is the posterior probability of observing non-zero migration rates in the Bayesian sampled trees.

* Bayes factor greater than 6 with indicator value greater than 0.50 was the minimum criteria for significance; 6≤BF<10 statistically significant; 10≤BF<30 strong statistical support; 30≤BF<100 very strongly supported; BF≥100 decisive.

## Discussion

Our goal was to understand the migration dynamics and diffusion patterns of influenza virus in their natural hosts by utilizing over 30 years of continuous systematic surveillance data. We show that our surveillance within Alberta, which includes convergence points for all four migratory flyways [28], [29], is capturing the majority of genetic diversity of the North American influenza gene pool. Breeding birds converging in this region facilitate the spread and generation of influenza virus genetic diversity indicating the importance of *Anseriformes'* social behavior in persistence of the virus population.

The site at Delaware Bay has been identified as a hotspot for avian influenza A viruses [30], where hundreds of thousands of migrating *Charadriiformes* stopover annually to feed in highly dense congregations. Our results showed limited genetic diversity coupled with high prevalence of infection indicating an epizootic in *Charadriiformes* that does not play a significant role in the shaping the sampled AIV diversity within North American *Anseriformes*. Even though this hotspot is not representative of gene pool diversity, these viruses are ultimately derived from the same population of viruses common throughout North America. The transmission of viruses between populations of birds is most likely occurring where migratory *Anseriformes* and *Charadriiformes* commingle, possibly in South and Central America or Arctic breeding grounds. The role of *Charadriiformes* in the persistence and transmission of influenza A viruses therefore warrants further study, especially on a more comprehensive spatial scale.

We show that the long-term persistence of the influenza A virus gene pool in North American wild birds may be independent of migratory flyways. Although virus migration could be restricted within a flyway over short time periods, our results show strong support for longer-term lateral diffusion of viral lineages between host populations. In our study, data points were not assigned to a flyway but discrete sites were assigned and used to inform within and between flyway migration rates using tip-dated time-dependent phylogenetic reconstructions. While this does contradict previous work by Lam et al [27], which suggested that migratory flyways and distance might represent a barrier for migration, both studies show that migration between flyways does occur [27]. Our study shows that the short-term evolutionary consequences of these ecological barriers may be rapidly erased by East-West virus migration, and that such diffusion may be critical for the survival and persistence of novel virus lineages introduced to North American wild birds.

Subtype specific host distribution, geographic state definition and host ecology may also be a source for the differences observed between the two studies [27]. While we found no correlation between distance migrated and rate of migration, analysis of the H3-HA indicated that subtype specific diffusion patterns might be different. In turn this may be related to host specificity of H3 viruses. Furthermore, in our study we cannot detect migration events where the distance migrated is less than 400 km due to the definition we used for geographic states (5′×5′ latitude-longitude square).

The data used in our analysis included collections from resident and short distance migratory birds [31]. This data was unavailable to Lam et al [27], and may further account for the observed differences. In our study we assume that virus migration was the same regardless of host. This assumption may be valid when analyzing viruses from all hosts in a single analysis, it is unlikely to be justified when considering specific hosts. Flyways are often applied universally to all hosts, whereas there are clear differences in the behavior and ecological habits of different hosts (see supporting information Text S1).

Using our model for virus transmission generalized predictions for movement of an introduced Eurasian virus and the associated risk for widespread diffusion can be inferred. An introduced virus lineage to Alaska might initially be restricted to the Pacific Flyway, but migration to a major congregation site such as Alberta could occur with subsequent spread across flyways occurring shortly after. While the establishment of introduced lineages into North America may be rare, introduction and reassortment events with Eurasian and North American strains probably occur more frequently than detected [16], [17], [32].

The development of fully resolved ecological and viral risk models depend upon the continued long-term active surveillance in major bird congregation zones. While the resolution and detection of migration events has been enhanced with increased surveillance in recent years, critical information for wild bird surveillance remains sparse. This is especially evident as no sampling in Central and South America was available for this study. A comprehensive understanding of spatial diffusion patterns of viruses introduced to wild animal populations is critical for the development of preparedness plans in response to emerging viral threats.

## Materials and Methods

### Sampling, virus isolation and sequencing

Systematic influenza surveillance has been conducted in ducks in Alberta, Canada since 1976, and in shorebirds and gulls at Delaware Bay (Delaware and New Jersey) since 1985. Ducks were sampled post-breeding and prior to southern migration during July through early September at various wetlands in the following regions of Alberta: Vermilion (1976–1978), Grand Prairie/Fairview (1979–1984, 1992–2011), Edmonton/Stettler (1979, 1981, 1983–2009), Brooks (1992–1995), and High River (1993–2000, 2002–2003, 2005–2007). Sampling occurred during duck banding operations conducted by the Canadian Wildlife Service after ducks were captured in swim-in bait traps. Birds banded in Alberta have been recovered in all four North American flyways but most mallards are recovered in the Central and pacific flyways. In 1984 samples were also collected from ducks captured in decoy traps during late April to early May in the Vermilion area. Overall, the majority of samples were obtained as cloacal swabs (n = 18,057) and tracheal/oropharyngeal specimens accounted for most of the remaining samples (n = 1,641; 1,293 of the oral swabs being collected since 2007). Hatch-year ducks were sampled more frequently than after-hatch-year ducks (n = 11,923 versus 7,559, respectively). A variety of duck species were sampled – primarily dabbling ducks. The most abundantly sampled species are mallard (*Anas platyrhynchos*), northern pintail (*Anas acuta*), and blue-winged teal (*Anas discors*) with these three species accounting for 93% of the total specimens. Other species (listed in decreasing rank order of samples obtained) include redhead (*Aythya americana*), green-winged teal (*Anas crecca*), american wigeon (*Anas americana*), gadwall (*Anas strepera*), canvasback (*Aythya valisineria*), lesser scaup (*Aythya affinis*), american coot (*Fulica americana*), northern shoveler (*Anas clypeata*), bufflehead (*Bucephala albeola*), cinnamon teal (*Anas cyanoptera*), common goldeneye (*Bucephala clangula*), ruddy duck (*Oxyura jamaicensis*), greater scaup (*Aythya marila*), hooded merganser (*Lophodytes cucullatus*), and wood duck (*Aix sponsa*).

Fecal samples from *Charadriiformes* – shorebirds and gulls - were collected in May at Delaware Bay from ruddy turnstone (*Arenaria interpres*), red knot (*Calidiris canutus*), semipalmated sandpiper (*Calidris pusilla*), sanderling (*Calidiris alba*), and dunlin (*Calidris alpina*) starting in 1985 and continuing to the present. Samples were also obtained from breeding colonies of gulls – primarily laughing gull (*Larus atricilla*) and herring gull (*Larus argentatus*). It is during this period in May that shorebirds (waders) are migrating north from South America to their breeding grounds in the Canadian Arctic. Delaware Bay serves as a stopover point where the birds can re-fuel on the abundance of eggs deposited by the coincident spawning of horseshoe crabs (*Limulus polyphemus*).

Although most of the 10,350 samples obtained were from freshly deposited feces on beaches we also collected 213 cloacal swabs from captured birds spanning the years 1986–1989 and 2000. A subset of 440 samples was collected outside of the May surveillance period at the following times; September 1985, September and November 1986, and June-September 1988. It should be noted that from 1988 through 2002 multiple swabs (usually 3) were combined to constitute a single sample vial. In the years prior to 1988 most sample vials contained an individual swab, and all samples since 2003 have been from single fecal deposits.

Approximately 19 sample sites were established around Delaware Bay and varied from year-to-year. Six sites were used on the west side of Delaware Bay in Maryland and Delaware from 1985 through 1989. Sampling was performed at 13 sites on the east side of the bay in New Jersey in all years. Table S1 summarizes prevalence and bird population estimates from Delaware Bay, the Prairie pothole region and the central flyway [33]–[37].

The majority of the swabs were derived from fecal deposits and therefore it was not possible to identify the species that served as the source of the sample in over half of the specimens. However, the birds tend to congregate in groups of like species, and gull feces were easily discriminated from other bird droppings, therefore in many instances we could attribute the source of the sample to a particular species. Otherwise the sample was considered “shorebird” or “gull”.

Swabs were collected using a dacron tipped applicator and placed in transport medium containing 50% phosphate buffered saline and 50% glycerol adjusted to pH 7.2 and supplemented with penicillin G, streptomycin, polymyxin B, gentamycin, and nystatin. In Alberta the duck swabs were placed immediately in liquid nitrogen and returned to the laboratory. Shorebird samples from Delaware Bay were immediately placed on ice and shipped to the laboratory within 6 days of collection. Storage of the specimens prior to testing was at −70°C.

Viruses were isolated in 10-day-old embryonated chicken eggs as previously described [38], [39]. Virus subtypes were determined by antigenic analysis in hemagglutination inhibition tests [38], neuraminidase inhibition tests, and/or by RT-PCR [40] and sequence analysis.

Through exploratory examination of surveillance records from Alberta and Delaware Bay we determined that H3 subtype viruses have been most frequently isolated throughout the time period 1985–2009. We therefore focused our sequencing efforts on this time period and randomly selected 200 viruses for full genome sequencing. This data was further supplemented with an additional 100 viruses randomly selected for genomic sequencing of various subtypes.

All samples were sequenced using a high-throughput Next-Generation sequencing pipeline at the JCVI that includes the 454/Roche GS-FLX and the Illumina HiSeq 2000. Viral RNA was first reverse transcribed and amplified by multi-segment RT-PCR (M-RTPCR) [41], which simultaneously and specifically amplifies all influenza A virus segments in a single reaction, irrespective of the virus subtype. The amplicons were barcoded and amplified using an optimized SISPA protocol [42]. Barcoded amplicons were quantitated, pooled and size selected (∼800 bp or ∼200 bp) and the pools were used for Next Generation library construction (50–100 viruses/library).

One library was prepared for sequencing on the 454/Roche GS-FLX platform using Titanium chemistry while the other was made into a library for sequencing on the Illumina HiSeq 2000. The sequence reads from the 454/Roche GS-FLX data were sorted by barcode, binned by sample, trimmed, searched by TBLASTX against custom nucleotide databases of full-length influenza A segments downloaded from GenBank to filter out both chimeric influenza sequences and non-influenza sequences amplified during the random hexamer-primed amplification. For each sample, the filtered 454/Roche GS-FLX reads were then binned by segment, and de novo assembled using CLC Bio's clc_novo_assemble program. The resulting contigs were searched against the corresponding custom full-length influenza segment nucleotide database to find the closest reference sequence for each segment. Because of the short read length of the sequences obtained from the barcode-trimmed Illumina, HiSeq 2000 these were not subjected to the TBLASTX filtering step. Both 454/Roche GS-FLX and Illumina HiSeq 2000 reads were then mapped to the selected reference influenza A virus segments using the clc_ref_assemble_long program.

At loci where both GS-FLX and Illumina sequence data agreed on a variation (as compared to the reference sequence), the reference sequence was updated to reflect the difference. A final mapping of all next-generation sequences to the updated reference sequences was then performed. Any regions of the viral genomes that were poorly covered or ambiguous after Next Generation sequencing were PCR amplified and sequenced using standard Sanger sequencing approach.

Through sequencing, some of these selected viruses have been identified as more than one isolate (“Mixed” in table S3). The direct sequencing method does not allow us to determine which internal gene segments are associated with which subtype. Furthermore, some variants could not yield unique gene sequences for each potential virus identified. Hence, some mixed variants contain more than 8 associated sequences, but fewer than 16. As such, these were not included in the analysis of genomic reassortment patterns. Other variants could not be completely sequenced and have subsequently been submitted as “Draft.” Out of the 300 variants submitted for sequencing, 287 full genomes have been completed. All data generated for this study has been made publicly available via the Influenza Virus Resource at NCBI [43] (Accession numbers CY101081to CY103740).

### Bayesian phylogenetic and coalescent analysis

We analyzed 1441 genomic sequences of influenza A viruses in wild birds (Table S2 shows NCBI accession numbers). For each dataset prepared we removed all recent introductions from Eurasia and focused this study solely on viral gene segments that have been circulating in North America for the last 25 years. Each internal gene dataset contained >1300 sequences. While no whole genomes with Eurasian origins were evident in the datasets examined, numerous reassortant genes with recent Eurasian ancestry were detected. The neuraminidase (NA) gene was not included in the analysis due to the deep divergence between NA subtypes, while distribution of locations and time was sparse or inconsistent for individual NA genes. However, H3-HA gene sequences were sampled throughout North America and we therefore analyzed all H3-HA gene sequences isolated from wild aquatic birds (ntax = 437).

We used time-stamped sequence data with a relaxed-clock Bayesian Markov chain Monte Carlo method as implemented in BEAST v1.6.2 and BEAST 2 for phylogenetic analysis [44], [45]. For all analyses we used the uncorrelated lognormal relaxed molecular clock to accommodate variation in molecular evolutionary rate amongst lineages, the SRD06 codon position model, with a different rate of nucleotide substitution for the 1^st^ plus 2^nd^ versus the 3^rd^ codon position, and the HKY85 substitution model then applied to these codon divisions [46]. This analysis was conducted with a time-aware linear Bayesian skyride coalescent tree prior over the unknown tree space with relatively uninformative priors on all model parameters a normal prior on the mean skyride size (log units) of 11.0 (standard deviation 1.8) [47]. We performed three independent analyses of 50 million generations. These analyses were combined after the removal of an appropriate burn-in (10%–20% of the samples in most cases) with 5000 generations sampled from each run for a total of 15,000 trees and parameter estimates.

We further compared relative genetic diversity and reassortment patterns of viral isolates from Alberta and Delaware Bay by estimating phylogenies as described above for these populations independently.

### Estimation of viral migration rates between discrete host populations using the internal gene sequences

Analysis of migration paths using single gene segments yields answers that do not have to agree with each other, due to multiple factors such as sampling bias and/or reassortment. Therefore, we implemented one inclusive analysis of all genes in which each gene is treated as an independent dataset, but shares the migration parameters with all other genes. In order to estimate migration patterns for a single subtype as well as an average migration pattern of the entire AIV gene pool we devised two datasets. The first dataset focused on seven gene segments from H3 influenza A (excluding NA) as this was the most commonly isolated subtype throughout the surveillance period in both Alberta and Delaware Bay. Secondly, we analyzed all publically available PB1, PB2, PA, NP, M gene segments (excluding recent introductions from Eurasia) to estimate the viral migration patterns across the entire population of birds regardless of subtype. HA, NA and NS genes were not included due to the deep divergence between subtypes. This latter analysis resulted in a dataset of more than 1300 sequences for each of the five genes included.

While the phylogeny and substitution rates were separate for each gene, based on a joint migration process a single migration matrix was estimated. We used a reversible continuous-time Markov chain model to estimate the migration rates between geographical regions and the general patterns of avian influenza A virus circulation in different populations [48]. In these analyses we used a constant-population coalescent process prior over the phylogenies and uncorrelated lognormal relaxed molecular clocks. Here we identified 16 discrete geographic regions, based on observed sampling locations, estimated from a 5′×5′ latitude-longitude square (Supporting Data Files; File S1, Table S2, S3, Figure S12), plus an additional character state containing taxa isolated prior to 1998 and locations with fewer than four sequences isolated. We selected discrete geographic sites based on the grid instead of assigning taxa to discrete flyways as these vary to a large degree between potential host populations and overlap between geographic zones. By defining the discrete characters in such a manner we were able to group a number of sampling sites and establish a parameter limit that could be addressed by the data available. A limitation of this approach is that migration rates between locations less than 400 km could not be detected. The ancestral states were mapped onto the internal nodes of phylogenetic trees sampled during the Bayesian analysis (Supporting Data Files; Figures S2, S3, S4, S5, S6, S7, S8). Given the large number of states, a Bayesian stochastic search variable selection (BSSVS) was employed to reduce the number of parameters to those with significantly non-zero transition rates [48]. The BSSVS explores and efficiently reduces the state space by employing a binary indicator (I) [48]. From the BSSVS results, a Bayes factor (BF) test can be applied to assess the support for individual transitions between discrete geographic states. The BF was deemed statistically significant where I>0.5 and the BF>6 from the combined independent analyses. Therefore our minimal critical cutoff for statistical supports were 6≤BF< 10 indicating substantial support, 10≤BF<30 indicating strong support, 30≤BF<100 indicates very strong support and BF>100 indicating decisive support [48]–[50]. Within flyway rate estimates were compared with between flyway rate estimates to determine if migration of the viral population was structured by flyway. The Pearson correlation coefficient and the Mantel statistical test of correlation (100000 permutations) were conducted to test correlation between migration rate and distance between sites.

### Statistical comparison of genomic phylogenies for reassortment

We used multidimensional scaling plots to visually assess the strength of reassortment in Alberta and Delaware Bay. In this analysis the tree-to-tree variation in branch lengths is visualized as a cloud of points where the centroid of the cloud represents the mean from the 500 trees used in the analysis. Here we assume that gene segments with similar evolutionary histories will occupy the similar locations in the 2-dimensional Euclidean space where the cloud of points should overlap. We used two metrics to assess the degree of reassortment of the influenza A virus populations in the two discrete sampling regions: the time to the most recent common ancestor (tMRCA) or patristic distances calculated from a posterior distribution of trees. From a posterior distribution of phylogenetic trees we estimated the tMRCA for influenza A viruses sampled in each location from each gene during each year and computed the correlation coefficient of the tMRCAs between each pair of trees. This method of tree to tree comparisons has been applied to seasonal influenza A viruses [26] where the uncertainty of the phylogenetic history in the Bayesian posterior sampling of trees for each influenza A gene segments was compared using the tMRCA estimated for annual seasonal influenza A virus outbreaks in two geographic locations.

In our data sets there was a sparseness of sampling through time, especially in Delaware Bay. Therefore we encountered high levels of uncertainty where no clear pattern was discernable and zero distances between trees resulted in computational errors by using the tMRCA to estimate phylogenetic uncertainty between gene trees. To overcome this we computed the correlation matrix of the pairwise tree distances. Here we calculated the correlation coefficient for each pair of trees using the patristic distances between every taxon, where the patristic distance is the sum of branch lengths between two nodes. The dissimilarity matrix was obtained by calculating one minus the correlation matrix.

### Ethics statement

All animal experiments were performed following Protocol Number 081 approved on August 19, 2011 by the St. Jude Children's Research Hospital Institutional Animal Care and Use Committee in compliance with the Guide for the Care and Use of Laboratory Animals, 8th Ed. These guidelines were established by the Institute of Laboratory Animal Resources and approved by the Governing Board of the U.S. National Research Council.

## Supporting Information

Figure S1 Neighbor joining phylogenetic tree produced from an HKY85 nucleotide substitution model optimized distance matrix from all available H3-HA data, including sequences generated in this study. The major lineages; Oceania, Eurasia, and North American Lineages I and II are indicated to the right of the tree. Bootstrap supports for these major lineages are indicated on the tree. The scale bar indicates nucleotide substitutions/site.
(PDF)

Figure S2 H3 Hemagglutinin gene tree nexus file. Temporally structured maximum clade credibility phylogenetic tree showing the mixing of avian influenza A virus isolated from North American wild birds for each individual gene dataset. Ancestral state changes recovered from the discrete phylogeographic analyses are indicated by color changes at tree nodes. Purple bars on nodes indicated 95% confidence intervals of date estimates. Trees with taxon labels and node annotations can be viewed in FigTree (available from http://tree.bio.ed.ac.uk/software/figtree/). Also applies to figures S3, S4, S5, S6, S7, S8.
(TREE)

Figure S3 PB2 gene tree nexus file.
(TREE)

Figure S4 PB1 gene tree nexus file.
(TREE)

Figure S5 PA gene tree nexus file.
(TREE)

Figure S6 NP gene tree nexus file.
(TREE)

Figure S7 M gene tree nexus file.
(TREE)

Figure S8 NS gene tree nexus file.
(TREE)

Figure S9 A) Mean migration rate per MCMC step within flyway migration rates vs Mean between flyway migration jointly estimated from a subsampled dataset of Figure S9 including 20 isolates per year and all H3 sequences available; B) Density distribution of mean within flyway and mean between flyway rates.
(PDF)

Figure S10 Relationship of migration rate and distance. A) Mean statistically supported rates vs distance between discrete migration sites; B) Median statistically supported rates vs distance between discrete migration sites; C) All Mean migration rates vs distance between discrete migration sites; D) All Median rate indicator vs distance between discrete migration sites.
(PDF)

Figure S11 Interactive Google Earth Supplementary Data. GenBank Accession numbers and specific location of virus sampling for all sequences used in this study in the 5° Latitude by 5° Longitude square used to define the discrete character for ancestral state reconstruction.
(KML)

Figure S12 PB2 gene tree nexus file used to estimate joint migration. Interactive Tree files. Temporally structured maximum clade credibility phylogenetic tree with all available data used to jointly estimate the migration patterns summarized in Figure 4. Ancestral state changes recovered from the discrete phylogeographic analyses are indicated by color changes at tree nodes. Purple bars on nodes indicated 95% confidence intervals of date estimates. Trees with taxon labels and node annotations can be viewed in FigTree (available from http://tree.bio.ed.ac.uk/software/figtree/). Also applies to figures S13, S14, S15, S16.
(TREE)

Figure S13 PB1 gene tree nexus file used to estimate joint migration.
(TREE)

Figure S14 PA gene tree nexus file used to estimate joint migration.
(TREE)

Figure S15 NP gene tree nexus file used to estimate joint migration.
(TREE)

Figure S16 M gene tree nexus file used to estimate joint migration.
(TREE)

File S1 BEAST2 executable xml file detailing the parameters for the joint estimation of the single migration rate matrix from independently generated phylogenies (BEAST2 available from http://beast2.cs.auckland.ac.nz/index.php/Main_Page).
(TXT)

Table S1 Host Avifauna most frequently infected with influenza A virus summarized from the Centers of Excellence for Influenza Research and Surveillance North American wild bird surveillance efforts reporting from 2007.
(DOC)

Table S2 GenBank Accession numbers, isolation date and location of virus sampling for additional sequences from public databases used in this study.
(DOC)

Table S3 Associated geographic metadata and exact date of sampling of newly sequenced avian influenza A viruses.
(DOC)

Table S4 Number of taxa included per protein coding region to estimate average migration dynamics between discrete regions.
(DOC)

Text S1 Supplementary information describing flyways and bird behavior.
(DOC)
