# Supplementary figures and images for: Influenza A Virus Migration and Persistence in North American Wild Birds
Source: PLoS Pathog. 2013 Aug 29;9(8):e1003570. doi: 10.1371/journal.ppat.1003570 (PMC3757048; doi:10.1371/journal.ppat.1003570)

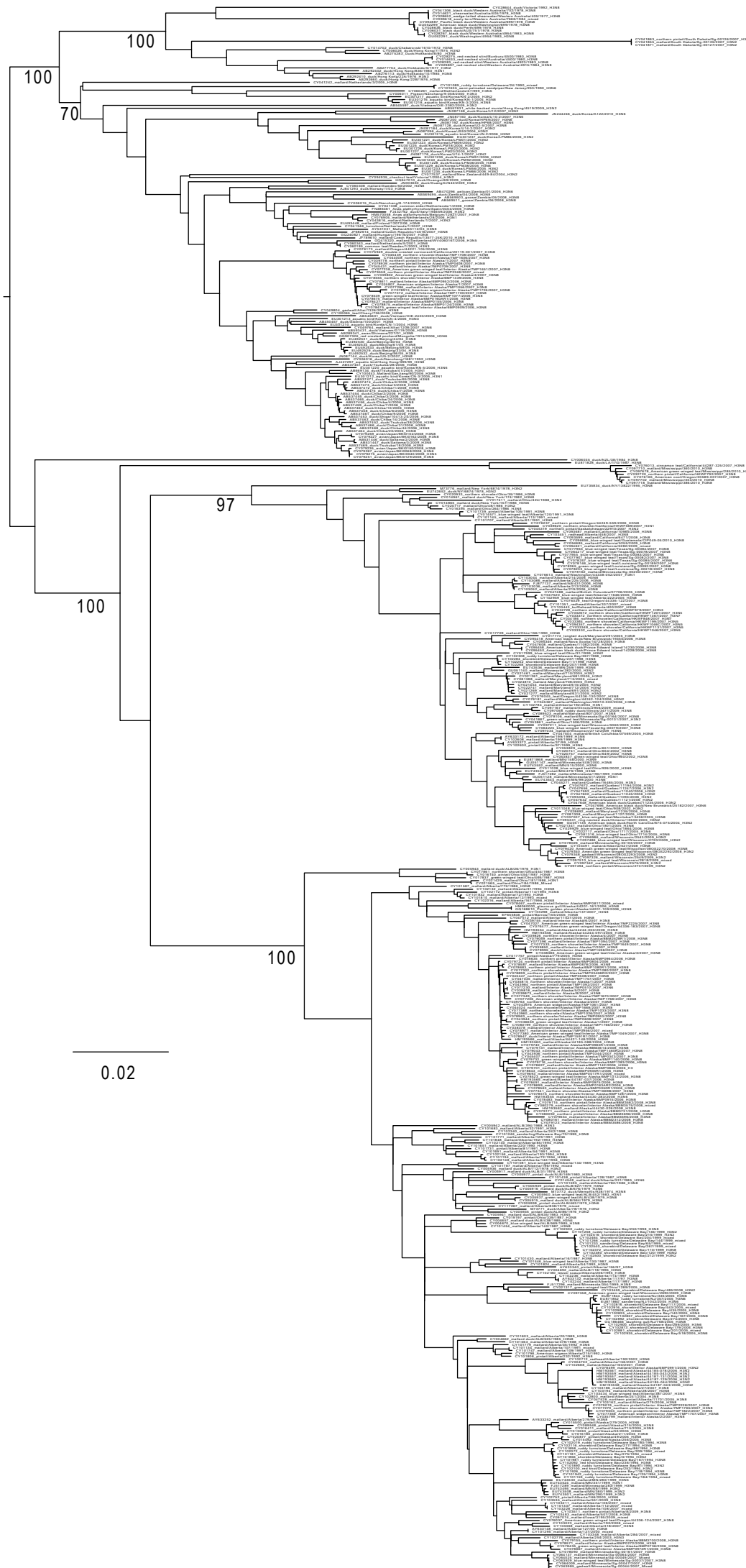

Oceania

North American  
Eurasian

North America  
Lineage I

North America  
Lineage II

Supplement: Figure S1 — Neighbor joining phylogenetic tree produced from an HKY85 nucleotide substitution model optimized distance matrix from all available H3-HA data, including sequences generated in this study. The major lineages; Oceania, Eurasia, and North American Lineages I and II are indicated to the right of the tree. Bootstrap supports for these major lineages are indicated on the tree. The scale bar indicates nucleotide substitutions/site. (PDF) [file ppat.1003570.s001.pdf]

**A****Mean migration rate per MCMC state**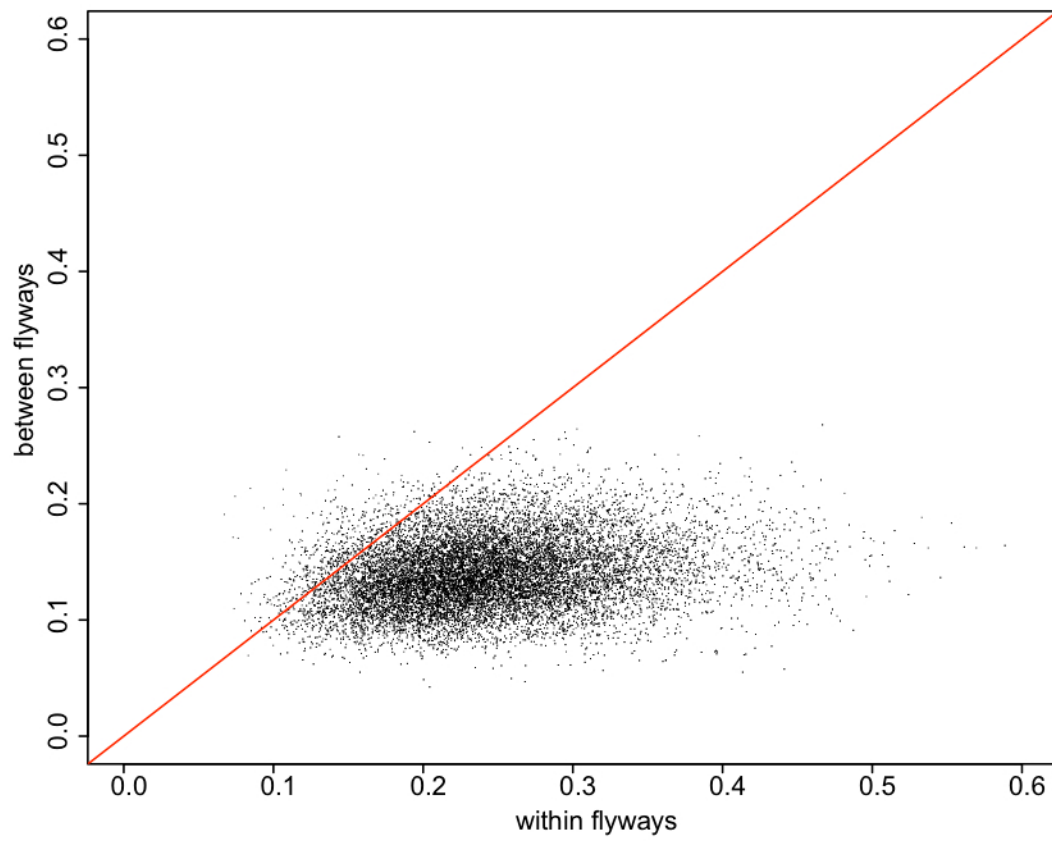**B****Mean within vs. between migration rate densities**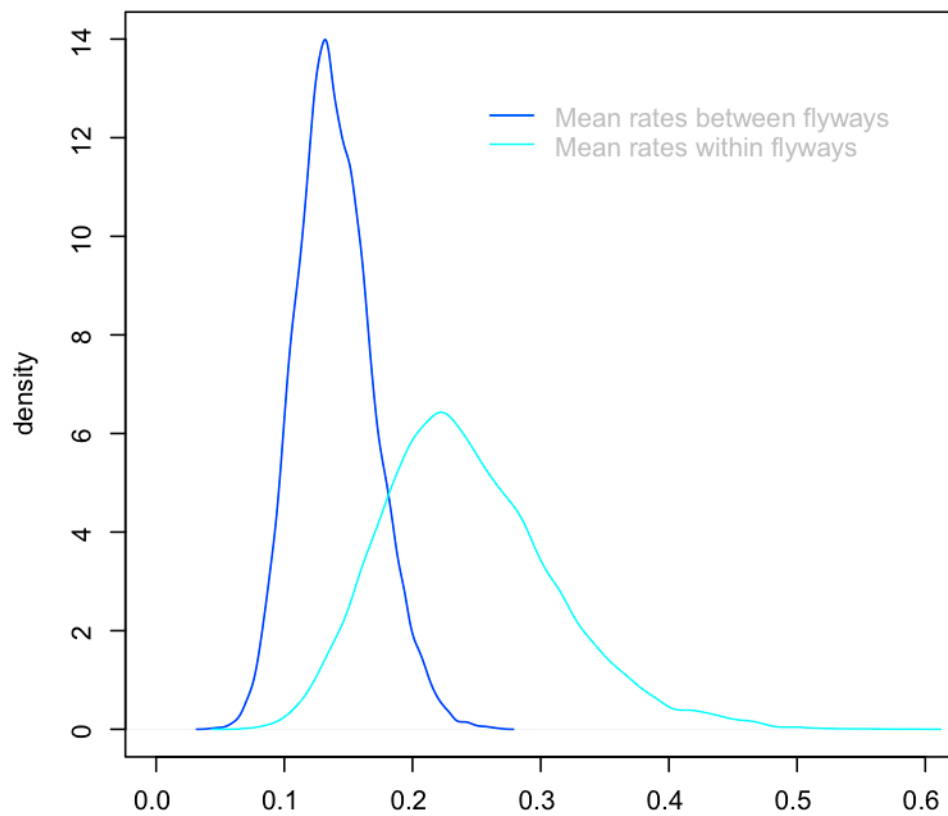

Supplement: Figure S9 — A) Mean migration rate per MCMC step within flyway migration rates vs Mean between flyway migration jointly estimated from a subsampled dataset of Figure S9 including 20 isolates per year and all H3 sequences available; B) Density distribution of mean within flyway and mean between flyway rates. (PDF) [file ppat.1003570.s009.pdf]
