# Supplementary material for: Influenza A Virus Migration and Persistence in North American Wild Birds
Source: PLoS Pathog. 2013 Aug 29;9(8):e1003570. doi: 10.1371/journal.ppat.1003570 (PMC3757048; doi:10.1371/journal.ppat.1003570)
Supplement: Figure S10 — Relationship of migration rate and distance. A) Mean statistically supported rates vs distance between discrete migration sites; B) Median statistically supported rates vs distance between discrete migration sites; C) All Mean migration rates vs distance between discrete migration sites; D) All Median rate indicator vs distance between discrete migration sites. (PDF) [file ppat.1003570.s010.pdf]

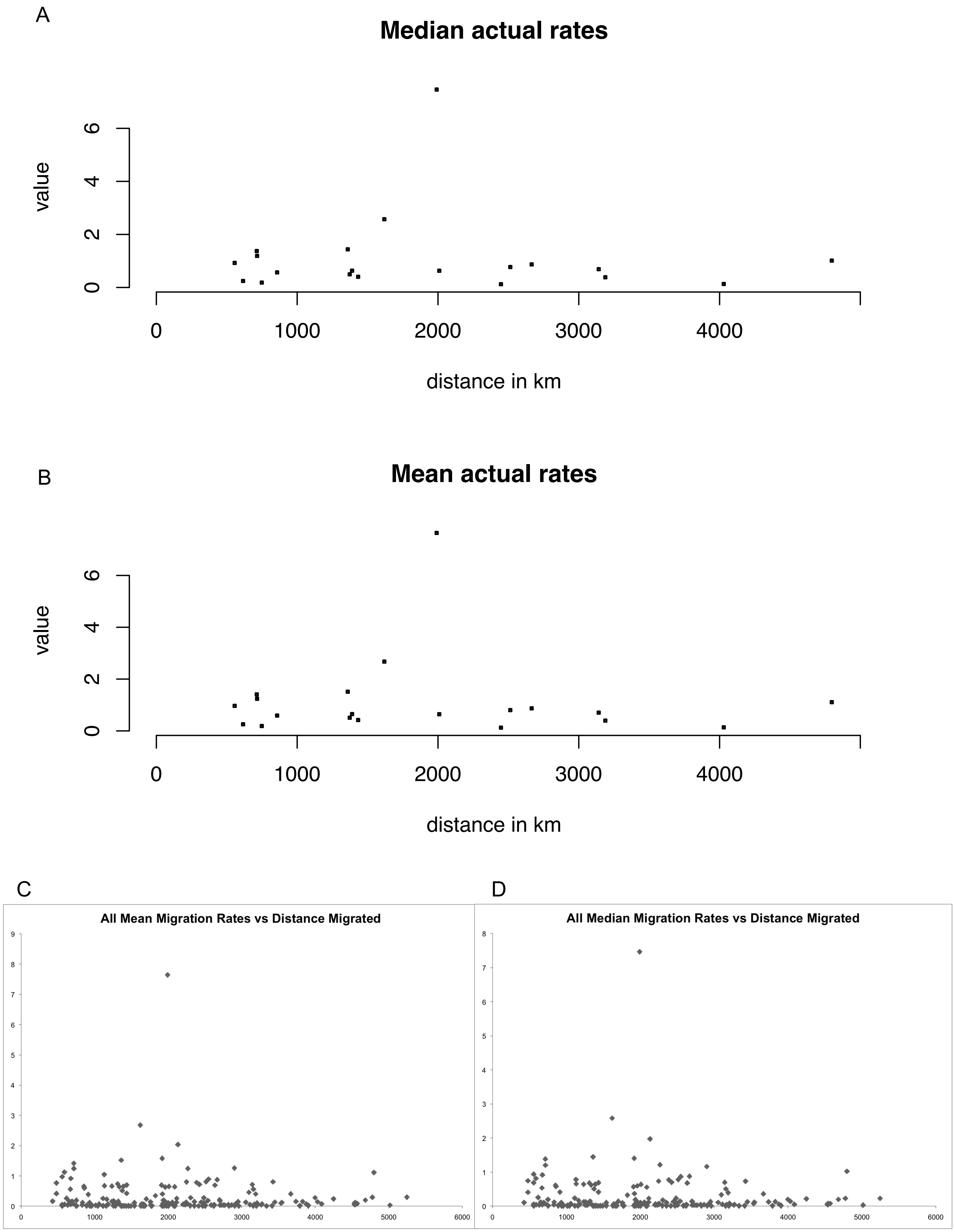
A
Median actual rates
value
0
2
4
6
0
1000
2000
3000
4000
distance in km
B
Mean actual rates
value
0
2
4
6
0
1000
2000
3000
4000
distance in km
C
All Mean Migration Rates vs Distance Migrated
0
1
2
3
4
5
6
7
8
9
0
1000
2000
3000
4000
5000
6000
D
All Median Migration Rates vs Distance Migrated
0
1
2
3
4
5
6
7
8
0
1000
2000
3000
4000
5000
6000
