# Supplementary material for: Influenza A Virus Migration and Persistence in North American Wild Birds
Source: PLoS Pathog. 2013 Aug 29;9(8):e1003570. doi: 10.1371/journal.ppat.1003570 (PMC3757048; doi:10.1371/journal.ppat.1003570)
Supplement: Table S1 — Host Avifauna most frequently infected with influenza A virus summarized from the Centers of Excellence for Influenza Research and Surveillance North American wild bird surveillance efforts reporting from 2007. (DOC) [file ppat.1003570.s018.doc]

**Table S1:** Host Avifauna most frequently infected with influenza A virus summarized from the Influenza Research Database [12] and bird population estimates [13-16].

|  |  |  |  |  | Prairie Pothole Region | | | Delaware Bay |
| --- | --- | --- | --- | --- | --- | --- | --- | --- |
| Species | Common Name | # birds sampled 2006-2009^32^ | % +’ve^12^ | Central Flyway° | AB | SK | MB | Pop count^13^ (Daily peak)^14^ |
| *Anas acuta* | Pintail | 7220 | 14.45 | 451 | 655 | 1,106 | 38 |  |
| *Anas platyrhynchos* | Mallard | 14330 | 14.35 | 1,572 | 939 | 2,093 | 521 |  |
| *Anas discors* | Blue-winged teal | 3182 | 8.52 | 77* | 470 | 2,489 | 393 |  |
| *Anas clypeata* | Shoveler | 3159 | 7.19 | 138 | 878 | 1,496 | 148 |  |
| *Aythya americana* | Redhead | 228 | 4.82 | 209 | 167 | 438 | 65 |  |
| *Anas cyanoptera* | Cinnamon Teal | 193 | 4.66 | 77* |  |  |  |  |
| *Bucephala albeola* | Bufflehead | 181 | 4.42 | 14 |  |  |  |  |
| *Aythya marila* | Greater Scaup | 168 | 4.17 | 216ǂ | 228 | 347 | 85 |  |
| *Aythya affinis* | Lesser Scaup | 1886 | 3.61 |  |
| *Anas americana* | American wigeon | 2559 | 3.87 | 223 | 200 | 281 | 5 |  |
| *Fulica americana* | American Coot | 109 | 3.67 | 529 |  |  |  |  |
| *Anas crecca* | Common teal | 1227 | 2.28 |  |  |  |  |  |
| *Aythya collaris* | Ring-necked duck | 644 | 2.02 | 278 |  |  |  |  |
| *Aythya valisineria* | Canvasback | 210 | 1.90 | 33 | 33 | 335 | 68 |  |
| *Anser albifrons* | Greater white-fronted goose | 627 | 1.75 | 128 | 710 | |  |  |
| *Anas strepera* | Gadwall | 1552 | 1.61 | 763 | 347 | 1,020 | 92 |  |
| *Anas carolinensis* | Green-winged teal | 16470 | 1.53 | 310 | 275 | 422 | 55 |  |
| *Arenaria interpres* | Ruddy turnstone | 1474 | 15.94 |  |  |  |  | 180 (65) |
| *Egretta thula* | Snowy egret | 143 | 3.50 |  |  |  |  | 215 |
| *Larus glaucescens* | Glaucous-winged gull | 2003 | 2.65 |  |  |  |  | 570 |
| *Larus atricilla* | Laughing gull | 278 | 2.52 |  |  |  |  | (30) |
| *Calidris minutilla* | Least sandpiper | 937 | 1.60 |  | 700 ^16^ | | | 102 |
| *Calidris canutus* | Red knot | 317 | 0.95 |  |  |  |  | 14^15^ |

°In thousands of birds

* Combined counts for these species

ǂ Combined counts for these species
