# Supplementary material for: Influenza A Virus Migration and Persistence in North American Wild Birds
Source: PLoS Pathog. 2013 Aug 29;9(8):e1003570. doi: 10.1371/journal.ppat.1003570 (PMC3757048; doi:10.1371/journal.ppat.1003570)
Supplement: Table S2 — GenBank Accession numbers, isolation date and location of virus sampling for additional sequences from public databases used in this study. (DOC) [file ppat.1003570.s019.doc]

**Table S2:** GenBank Accession numbers, isolation date and location of virus sampling for additional sequences from public databases used in this study. Blank fields indicate location was estimated as middle of state.

| **GenBank#** | **Name** | **Isolation Date** | **Latitude** | **Longitude** |
| --- | --- | --- | --- | --- |
| CY014807 | A/mallard/Tennessee/11457/1985 (H11N9) | 1/16/85 | 36.062422 | -86.476135 |
| CY003864 | A/laughing gull/New Jersey/75/1985 (H2N9) | 5/29/85 | 39.047 | -74.92 |
| CY004406 | A/ruddy turnstone/New Jersey/65/1985 (H7N3) | 5/29/85 | 39.047 | -74.92 |
| CY004818 | A/ruddy turnstone/New Jersey/47/1985 (H4N6) | 5/29/85 | 39.047 | -74.92 |
| CY004187 | A/mallard duck/ALB/10/1985 (H6N2) | 8/13/85 | 50.3 | -113.4 |
| CY004195 | A/blue-winged teal/ALB/69/1985 (H6N2) | 8/13/85 | 50.3 | -113.4 |
| CY004203 | A/mallard duck/ALB/76/1985 (H6N3) | 8/13/85 | 50.3 | -113.4 |
| CY004211 | A/pintail duck/ALB/111/1985 (H6N2) | 8/13/85 | 50.3 | -113.4 |
| CY004219 | A/shoveler/ALB/114/1985 (H6N2) | 8/13/85 | 50.3 | -113.4 |
| CY005107 | A/mallard duck/ALB/98/1985 (H6N2) | 8/13/85 | 50.3 | -113.4 |
| CY005051 | A/green-winged teal/ALB/228/1985 (H7N3) | 8/15/85 | 50.3 | -113.4 |
| CY003880 | A/mallard duck/Alberta/376/1985 (H2N3) | 8/18/85 | 50.3 | -113.4 |
| CY014549 | A/mallard duck/Alberta/331/1985 (H3N6) | 8/18/85 | 50.3 | -113.4 |
| CY005058 | A/mallard duck/Alberta/435/1985 (H7N3) | 8/20/85 | 50.3 | -113.4 |
| CY004663 | A/mallard duck/ALB/525/1985 (H3N8) | 8/22/85 | 50.3 | -113.4 |
| CY004671 | A/blue-winged teal/ALB/569/1985 (H3N8) | 8/22/85 | 50.3 | -113.4 |
| CY004854 | A/pintail duck/ALB/623/1985 (H4N6) | 8/27/85 | 50.3 | -113.4 |
| CY003888 | A/sanderling/New Jersey/766/1986 (H2N7) | 5/12/86 | 39.047 | -74.92 |
| CY003923 | A/laughing gull/New Jersey/798/1986 (H2N7) | 5/12/86 | 39.047 | -74.92 |
| CY004451 | A/herring gull/NJ/782/1986 (H13N2) | 5/12/86 | 39.047 | -74.92 |
| CY003895 | A/herring gull/DE/475/1986 (H13N2) | 6/1/86 | 39.047 | -74.92 |
| CY005376 | A/herring gull/Delaware/471/1986 (H2N7) | 6/1/86 | 38.85789 | -75.257721 |
| CY005421 | A/sanderling/Delaware/1258/1986 (H6N6) | 9/9/86 | 38.85789 | -75.257721 |
| CY014858 | A/mallard duck/New York/180/1986 (H4N9) | 10/4/86 | 43.205176 | -74.086304 |
| CY017694 | A/mallard/Ohio/30/1986 (H2N1) | 10/20/86 | 40.161034 | -82.888412 |
| CY020718 | A/mallard/Ohio/48/1986 (H3N2) | 10/20/86 | 40.161034 | -82.888412 |
| CY015468 | A/mallard/Ohio/83/1986 (H4N6) | 10/24/86 | 40.161034 | -82.888412 |
| CY018016 | A/mallard/Ohio/102/1986 (H11N3) | 10/24/86 | 40.161034 | -82.888412 |
| CY021574 | A/mallard/Ohio/81/1986 (mixed) | 10/24/86 | 40.161034 | -82.888412 |
| CY016165 | A/green-winged teal/Ohio/86/1986 (H6N2) | 10/26/86 | 40.161034 | -82.888412 |
| CY013249 | A/mallard/Ohio/338/1986 (H4N8) | 10/29/86 | 40.161034 | -82.888412 |
| CY015460 | A/green-winged teal/Ohio/344/1986 (H4N2) | 10/30/86 | 40.161034 | -82.888412 |
| CY081243 | A/mallard/Ohio/353/1986 (mixed) | 10/30/86 | 40.161034 | -82.888412 |
| CY021430 | A/mallard/Ohio/181/1986 (H3N1) | 11/6/86 | 40.161034 | -82.888412 |
| CY017766 | A/Black Duck/Ohio/194/1986 (H11N1) | 11/6/86 | 40.161034 | -82.888412 |
| CY018878 | A/green-winged teal/Ohio/175/1986 (H2N1) | 11/6/86 | 40.161034 | -82.888412 |
| CY021566 | A/mallard/Ohio/184/1986 (mixed) | 11/6/86 | 40.161034 | -82.888412 |
| CY021614 | A/Black Duck/Ohio/239/1986 (H11N9) | 11/7/86 | 40.161034 | -82.888412 |
| CY016396 | A/mallard/Ohio/264/1986 (H3N8) | 11/8/86 | 40.161034 | -82.888412 |
| CY005127 | A/ruddy turnstone/DE/2731/1987 (H9N1) | 5/27/87 | 39.047 | -74.92 |
| CY005114 | A/ruddy turnstone/DE/2576/1987 (H9N5) | 6/2/87 | 39.047 | -74.92 |
| CY005121 | A/laughing gull/Delaware/2718/1987 (H9N5) | 6/9/87 | 39.047 | -74.92 |
| CY005065 | A/laughing gull/DE/2838/1987 (H13N2) | 6/12/87 | 39.047 | -74.92 |
| CY004413 | A/knot/DE/2552/1987 (H9N5) | 7/7/87 | 39.047 | -74.92 |
| CY004997 | A/mallard/Alberta/7/1987 (H8N4) | 8/4/87 | 50.3 | -113.4 |
| CY004516 | A/coot/ALB/134/1987 (H6N2) | 8/13/87 | 53.555978 | -116.343541 |
| CY004524 | A/mallard duck/ALB/294/1987 (H6N2) | 8/26/87 | 50.3 | -113.4 |
| CY015452 | A/mallard/Ohio/344/1987 (H6N2) | 10/19/87 | 40.161034 | -82.888412 |
| CY011057 | A/mallard/Ohio/275/1987 (H4N2) | 10/19/87 | 40.161034 | -82.888412 |
| CY016125 | A/black duck/Ohio/307/1987 (H6N2) | 10/19/87 | 40.161034 | -82.888412 |
| CY016133 | A/pintail/Ohio/351/1987 (H6N2) | 10/19/87 | 40.161034 | -82.888412 |
| CY017276 | A/mallard/Ohio/265/1987 (H1N9) | 10/19/87 | 40.161034 | -82.888412 |
| CY019198 | A/pintail/Ohio/339/1987 (H3N8) | 10/19/87 | 40.161034 | -82.888412 |
| CY016141 | A/pintail/Ohio/454/1987 (H3N8) | 10/23/87 | 40.161034 | -82.888412 |
| CY021622 | A/mallard/Ohio/421/1987 (H7N8) | 10/23/87 | 40.161034 | -82.888412 |
| CY004861 | A/ruddy turnstone/DE/512/1988 (H4N6) | 5/15/88 | 39.047 | -74.92 |
| CY005134 | A/ruddy turnstone/DE/510/1988 (H9N6) | 5/15/88 | 39.047 | -74.92 |
| CY003915 | A/herring gull/DE/703/1988 (H2N8) | 5/16/88 | 39.047 | -74.92 |
| CY003908 | A/herring gull/DE/698/1988 (H2N1) | 5/16/88 | 39.047 | -74.92 |
| CY004555 | A/herring gull/DE/692/1988 (H2N8) | 5/16/88 | 39.047 | -74.92 |
| CY004562 | A/herring gull/Delaware/712/1988 (H16N3) | 5/16/88 | 39.047 | -74.92 |
| CY004568 | A/ruddy turnstone/DE/773/1988 (H9N6) | 5/17/88 | 39.047 | -74.92 |
| CY005072 | A/ruddy turnstone/DE/2378/1988 (H7N7) | 5/31/88 | 39.047 | -74.92 |
| CY003930 | A/mallard duck/Alberta/323/1988 (H2N1) | 9/3/88 | 50.3 | -113.4 |
| CY003937 | A/mallard/Alberta/353/1988 (H2N3) | 9/3/88 | 50.3 | -113.4 |
| CY004532 | A/mallard/Alberta/322/1988 (H1N1) | 9/3/88 | 50.3 | -113.4 |
| CY005141 | A/mallard duck/Alberta/321/1988 (H9N2) | 9/3/88 | 50.3 | -113.4 |
| CY004681 | A/mallard/ALB/394/1988 (H3N3) | 9/4/88 | 50.3 | -113.4 |
| CY012833 | A/pintail/Ohio/294/1988 (H6N2) | 10/13/88 | 40.161034 | -82.888412 |
| CY017702 | A/mallard/Ohio/324/1988 (H4N6) | 10/13/88 | 40.161034 | -82.888412 |
| CY011249 | A/wigeon/Ohio/379/1988 (H5N2) | 10/15/88 | 40.161034 | -82.888412 |
| CY016420 | A/mallard/Ohio/409/1988 (H12N5) | 10/15/88 | 40.161034 | -82.888412 |
| CY004095 | A/laughing gull/NJ/276/1989 (H6N8) | 4/22/89 | 39.047 | -74.92 |
| CY004392 | A/herring gull/NJ/402/1989 (H5N3) | 4/23/89 | 39.047 | -74.92 |
| CY005079 | A/red knot/NJ/325/1989 (H7N7) | 4/26/89 | 39.047 | -74.92 |
| CY012817 | A/mallard/Ohio/97/1989 (H4N2) | 10/19/89 | 40.161034 | -82.888412 |
| CY016173 | A/green-winged teal/Ohio/59/1989 (H6N8) | 10/19/89 | 40.161034 | -82.888412 |
| CY017782 | A/mallard/Ohio/99/1989 (H10N7) | 10/19/89 | 40.161034 | -82.888412 |
| CY020846 | A/pintail/Ohio/73/1989 (H6N2) | 10/19/89 | 40.161034 | -82.888412 |
| CY020926 | A/mallard/Ohio/122/1989 (H10N7) | 10/19/89 | 40.161034 | -82.888412 |
| CY081293 | A/mallard/Ohio/65/1989 (mixed) | 10/19/89 | 40.161034 | -82.888412 |
| CY089558 | A/mallard/Ohio/42/1989 (H10N6) | 10/19/89 | 40.161034 | -82.888412 |
| CY004235 | A/mallard duck/ALB/191/1990 (H6N3) | 8/1/90 | 50.3 | -113.4 |
| CY004227 | A/mallard duck/ALB/155/1990 (H6N3) | 8/4/90 | 50.3 | -113.4 |
| CY004897 | A/blue-winged teal/ALB/136/1990 (H4N3) | 8/22/90 | 50.3 | -113.4 |
| CY004243 | A/mallard duck/ALB/253/1990 (H6N3) | 8/29/90 | 50.3 | -113.4 |
| CY012801 | A/mallard/Ohio/171/1990 (H1N1) | 10/18/90 | 40.161034 | -82.888412 |
| CY017710 | A/mallard/Ohio/156/1990 (H3N6) | 10/18/90 | 40.161034 | -82.888412 |
| CY005148 | A/mallard/Alberta/11/1991 (H9N2) | 8/9/91 | 50.3 | -113.4 |
| CY005154 | A/mallard/Alberta/17/1991 (H9N2) | 8/9/91 | 50.3 | -113.4 |
| CY005305 | A/mallard/ALB/124/1991 (H11N2) | 8/26/91 | 50.3 | -113.4 |
| CY005351 | A/green-winged teal/ALB/199/1991 (H12N5) | 8/26/91 | 50.3 | -113.4 |
| CY004251 | A/mallard/ALB/199/1992 (H6N5) | 12/1/92 | 50.3 | -113.4 |
| CY004540 | A/blue-winged teal/Alberta/141/1992 (H1N1) | 12/1/92 | 50.3 | -113.4 |
| CY005002 | A/mallard/Alberta/194/1992 (H8N4) | 12/1/92 | 50.3 | -113.4 |
| CY015136 | A/ruddy turnstone/Delaware/34/1993 (H2N1) | 5/15/93 | 38.85789 | -75.257721 |
| CY015444 | A/black duck/Ohio/95/1993 (H1N1) | 10/15/93 | 40.161034 | -82.888412 |
| CY018886 | A/mallard/Ohio/118/1993 (H1N1) | 10/15/93 | 40.161034 | -82.888412 |
| CY004259 | A/pintail/ALB/179/1993 (H6N1) | 12/1/93 | 50.3 | -113.4 |
| CY005086 | A/pintail/Alberta/129/1993 (H10N7) | 12/1/93 | 50.3 | -113.4 |
| CY004904 | A/blue-winged teal/ALB/293/1994 (H4N6) | 7/1/94 | 50.3 | -113.4 |
| CY004912 | A/mallard/ALB/49/1995 (H4N6) | 8/1/95 | 50.3 | -113.4 |
| CY005225 | A/mallard/ALB/5/1995 (H10N1) | 8/1/95 | 50.3 | -113.4 |
| CY004693 | A/mallard/Alberta/118/1995 (H3N5) | 8/1/95 | 50.3 | -113.4 |
| CY003961 | A/blue-winged teal/Alberta/16/1997 (H2N9) | 10/24/97 | 50.3 | -113.4 |
| CY004380 | A/mallard/Alberta/52/1997 (H12N5) | 10/31/97 | 50.3 | -113.4 |
| CY016189 | A/Mallard/Ohio/322/1998 (H7N3) | 1/13/98 | 40.161034 | -82.888412 |
| CY015128 | A/ruddy turnstone/Delaware/105/1998 (H6N8) | 5/18/98 | 39.047 | -74.92 |
| CY004926 | A/mallard/Alberta/47/98 (H4N1) | 8/5/98 | 50.3 | -113.4 |
| CY004508 | A/mallard/ALB/201/1998 (H1N1) | 8/11/98 | 50.3 | -113.4 |
| CY003977 | A/mallard/Alberta/226/98 (H2N3) | 8/11/98 | 50.3 | -113.4 |
| CY003969 | A/mallard/Alberta/205/98 (H2N3) | 8/11/98 | 53.555978 | -116.343541 |
| CY015477 | A/mallard/Ohio/249/1998 (H6N1) | 10/17/98 | 40.161034 | -82.888412 |
| CY018008 | A/green-winged teal/Ohio/203/1998 (H6N2) | 10/17/98 | 40.161034 | -82.888412 |
| CY020870 | A/mallard/Ohio/217/1998 (H6N8) | 10/17/98 | 40.161034 | -82.888412 |
| CY014889 | A/duck/New York/16873/1999 (H6N2) | 5/25/99 | 43.205176 | -74.086304 |
| CY016157 | A/gadwall/Ohio/37/1999 (H6N2) | 10/16/99 | 40.161034 | -82.888412 |
| CY012825 | A/mallard/Ohio/56/1999 (H1N1) | 10/16/99 | 40.161034 | -82.888412 |
| CY017726 | A/pintail/Ohio/25/1999 (H1N1) | 10/16/99 | 40.161034 | -82.888412 |
| CY018000 | A/blue-winged teal/Ohio/31/1999 (H3N2) | 10/16/99 | 40.161034 | -82.888412 |
| CY081365 | A/green-winged teal/Ohio/196/1999 (mixed) | 10/23/99 | 40.161034 | -82.888412 |
| CY013864 | A/green-winged teal/Ohio/178/1999 (H6N2) | 10/23/99 | 40.161034 | -82.888412 |
| CY015485 | A/mallard/Ohio/170/1999 (H6N5) | 10/23/99 | 40.161034 | -82.888412 |
| CY016956 | A/mallard/Ohio/66/1999 (H1N1) | 10/23/99 | 40.161034 | -82.888412 |
| CY017718 | A/green-winged teal/Ohio/72/1999 (H1N1) | 10/23/99 | 40.161034 | -82.888412 |
| CY005358 | A/laughing gull/Delaware/94/2000 (H12N4) | 5/15/00 | 38.85789 | -75.257721 |
| CY005365 | A/ruddy turnstone/Delaware/97/2000 (H12N5) | 5/15/00 | 38.85789 | -75.257721 |
| CY005324 | A/pintail/Alberta/84/2000 (H11N9) | 8/7/00 | 50.3 | -113.4 |
| CY005243 | A/pintail/Alberta/202/2000 (H10N7) | 8/18/00 | 50.3 | -113.4 |
| CY005250 | A/mallard/Alberta/208/2000 (H10N7) | 8/19/00 | 50.3 | -113.4 |
| CY004940 | A/mallard/Alberta/30/2001 (H4N8) | 7/27/01 | 50.3 | -113.4 |
| CY005093 | A/mallard/Alberta/34/2001 (H7N1) | 7/27/01 | 50.3 | -113.4 |
| CY004703 | A/mallard/Alberta/156/2001 (H3N8) | 8/6/01 | 50.3 | -113.4 |
| CY004947 | A/pintail/Alberta/269/2001 (H4N6) | 8/14/01 | 50.3 | -113.4 |
| CY020886 | A/Black Duck/Ohio/415/2001 (H7N3) | 8/30/01 | 40.161034 | -82.888412 |
| CY020822 | A/mallard/Maryland/172/2002 (H6N2) | 6/26/02 | 39.076243 | -76.557541 |
| CY032206 | A/mallard/Maryland/350/2002 (H1N1) | 7/25/02 | 39.076243 | -76.557541 |
| CY003985 | A/mallard/Alberta/149/2002 (H2N4) | 8/2/02 | 50.3 | -113.4 |
| CY020814 | A/mallard/Maryland/470/2002 (H6N2) | 8/14/02 | 39.076243 | -76.557541 |
| CY004547 | A/pintail duck/Alberta/210/2002 (H1N1) | 8/16/02 | 50.3 | -113.4 |
| CY020758 | A/mallard/Ohio/649/2002 (H3N8) | 8/19/02 | 40.161034 | -82.888412 |
| CY020774 | A/mallard/Ohio/655/2002 (H4N6) | 8/19/02 | 40.161034 | -82.888412 |
| CY020782 | A/mallard/Ohio/666/2002 (H6N2) | 8/19/02 | 40.161034 | -82.888412 |
| CY020790 | A/mallard/Ohio/668/2002 (H4N6) | 8/19/02 | 40.161034 | -82.888412 |
| CY020838 | A/mallard/Ohio/653/2002 (H6N2) | 8/19/02 | 40.161034 | -82.888412 |
| CY021582 | A/mallard/Ohio/656/2002 (mixed) | 8/19/02 | 40.161034 | -82.888412 |
| CY053830 | A/mallard/Ohio/651/2002 (H3N8) | 8/19/02 | 40.161034 | -82.888412 |
| CY081251 | A/mallard/Ohio/662/2002 (mixed) | 8/19/02 | 40.161034 | -82.888412 |
| CY011037 | A/mallard/Ohio/657/2002 (H4N6) | 8/19/02 | 40.161034 | -82.888412 |
| CY020726 | A/mallard/Ohio/667/2002 (H4N6) | 8/19/02 | 40.161034 | -82.888412 |
| CY020742 | A/mallard/Ohio/654/2002 (H3N8) | 8/19/02 | 40.161034 | -82.888412 |
| CY020830 | A/mallard/Ohio/648/2002 (H6N2) | 8/19/02 | 40.161034 | -82.888412 |
| CY020854 | A/mallard/Ohio/664/2002 (H6N6) | 8/19/02 | 40.161034 | -82.888412 |
| CY096747 | A/mallard/Ohio/669/2002 (mixed) | 8/19/02 | 40.161034 | -82.888412 |
| CY020734 | A/mallard/Maryland/750/2002 (H4N6) | 8/27/02 | 39.076243 | -76.557541 |
| CY016612 | A/mallard/Maryland/789/2002 (H5N2) | 8/27/02 | 39.076243 | -76.557541 |
| CY020766 | A/black duck/Maryland/834/2002 (H4N8) | 8/28/02 | 39.076243 | -76.557541 |
| CY081235 | A/black duck/Maryland/839/2002 (mixed) | 8/28/02 | 39.076243 | -76.557541 |
| CY021590 | A/mallard/Maryland/899/2002 (mixed) | 8/29/02 | 39.076243 | -76.557541 |
| CY011113 | A/mallard/Maryland/881/2002 (H6N2) | 8/29/02 | 39.076243 | -76.557541 |
| CY011049 | A/blue-winged teal/Ohio/908/2002 (H3N2) | 9/7/02 | 40.161034 | -82.888412 |
| CY020750 | A/blue-winged teal/Ohio/989/2002 (H4N6) | 9/7/02 | 40.161034 | -82.888412 |
| CY020862 | A/blue-winged teal/Ohio/907/2002 (H1N6) | 9/7/02 | 40.161034 | -82.888412 |
| CY011029 | A/blue-winged teal/Ohio/926/2002 (H3N8) | 9/7/02 | 40.161034 | -82.888412 |
| CY053838 | A/green-winged teal/Ohio/960/2002 (H3N8) | 9/7/02 | 40.161034 | -82.888412 |
| CY081227 | A/mallard/Maryland/1027/2002 (mixed) | 9/17/02 | 39.076243 | -76.557541 |
| CY004421 | A/laughing gull/DE/5/2003 (H9N1) | 5/19/03 | 39.047 | -74.92 |
| CY005331 | A/shorebird/DE/236/2003 (H11N9) | 5/30/03 | 39.047 | -74.92 |
| CY004339 | A/pintail duck/Alberta/49/2003 (H12N5) | 7/25/03 | 50.3 | -113.4 |
| CY003993 | A/mallard/Alberta/79/2003 (H2N3) | 8/1/03 | 50.3 | -113.4 |
| CY004275 | A/mallard/Alberta/154/2003 (H6N5) | 8/3/03 | 50.3 | -113.4 |
| CY004353 | A/mallard/Alberta/209/2003 (H10N7) | 8/7/03 | 50.3 | -113.4 |
| CY004954 | A/blue-winged teal/Alberta/293/2003 (H4N6) | 8/13/03 | 50.3 | -113.4 |
| CY004283 | A/shorebird/DE/12/2004 (H6N8) | 5/15/04 | 39.047 | -74.92 |
| CY004399 | A/shorebird/DE/101/2004 (H5N7) | 5/17/04 | 39.047 | -74.92 |
| CY004444 | A/shorebird/DE/68/2004 (H13N9) | 5/17/04 | 39.047 | -74.92 |
| CY004977 | A/shorebird/DE/75/2004 (H5N7) | 5/17/04 | 39.047 | -74.92 |
| CY005257 | A/shorebird/DE/122/2004 (H10N7) | 5/17/04 | 39.047 | -74.92 |
| CY018902 | A/blue-winged teal/Ohio/658/2004 (H7N3) | 9/1/04 | 40.161034 | -82.888412 |
| CY034847 | A/Muscovy duck/New York/11646-4/2005 (H7N2) | 2/1/05 | 43.205176 | -74.086304 |
| CY029842 | A/muscovy duck/New York/21211-5/2005 (H1N1) | 3/1/05 | 43.205176 | -74.086304 |
| CY031708 | A/Muscovy duck/New York/23164-10/2005 (H7N2) | 3/4/05 | 43.205176 | -74.086304 |
| CY033226 | A/Muscovy duck/New York/23165-13/2005 (H7N2) | 3/4/05 | 43.205176 | -74.086304 |
| CY033266 | A/Muscovy duck/New York/30732-13/2005 (H7N2) | 3/23/05 | 43.205176 | -74.086304 |
| CY095053 | A/Muscovy duck/New York/31621-10/2005 (H7N2) | 3/24/05 | 43.205176 | -74.086304 |
| CY081309 | A/mallard/Maryland/1127/2005 (H3N8) | 6/27/05 | 39.076243 | -76.557541 |
| CY081389 | A/mallard/Maryland/715/2005 (mixed) | 6/27/05 | 39.076243 | -76.557541 |
| CY081301 | A/mallard/Maryland/1111/2005 (H11N9) | 6/27/05 | 39.076243 | -76.557541 |
| CY034791 | A/Muscovy duck/New York/87493-3/2005 (H7N2) | 7/14/05 | 43.205176 | -74.086304 |
| CY075926 | A/common murre/Oregon/19497-004/2005 (H9N5) | 7/25/05 | 43.952293 | -121.420898 |
| CY047537 | A/green-winged teal/Alberta/11383/2005 (H4N6) | 7/31/05 | 56.2129 | -118.6053 |
| CY047513 | A/mallard/Alberta/11527/2005 (H3N8) | 8/3/05 | 56.2313 | -117.9727 |
| CY047521 | A/blue-winged teal/Alberta/11646/2005 (H3N8) | 8/5/05 | 52.4343 | -112.9526 |
| CY047529 | A/northern pintail/Alberta/11701/2005 (H3N8) | 8/5/05 | 52.4343 | -112.9526 |
| CY047545 | A/redhead/Alberta/11817/2005 (H4N6) | 8/6/05 | 52.4343 | -112.9526 |
| CY013264 | A/pintail/Alaska/53/2005 (H3N6) | 8/8/05 | 64.98129 | -149.6911 |
| CY020878 | A/pintail/Alaska/49/2005 (H3N8) | 8/8/05 | 64.98129 | -149.6911 |
| CY039384 | A/mallard/Alberta/12017/2005 (H2N3) | 8/8/05 | 50.3 | -113.4 |
| CY012841 | A/pintail duck/Alaska/20/2005 (H12N5) | 8/9/05 | 64.98129 | -149.6911 |
| CY017734 | A/pintail duck/Alaska/102/2005 (H12N5) | 8/9/05 | 64.98129 | -149.6911 |
| CY096757 | A/pintail/Alaska/99/2005 (mixed) | 8/9/05 | 64.98129 | -149.6911 |
| CY016181 | A/pintail/Alaska/211/2005 (H3N8) | 8/10/05 | 64.98129 | -149.6911 |
| CY015493 | A/mallard/Alaska/256/2005 (H3N8) | 8/11/05 | 64.98129 | -149.6911 |
| CY015501 | A/pintail/Alaska/279/2005 (H3N8) | 8/11/05 | 64.98129 | -149.6911 |
| CY017742 | A/pintail/Alaska/310/2005 (H4N6) | 8/11/05 | 64.98129 | -149.6911 |
| CY081260 | A/mallard/Alaska/312/2005 (mixed) | 8/11/05 | 64.98129 | -149.6911 |
| CY081278 | A/pintail/Alaska/314/2005 (mixed) | 8/11/05 | 64.98129 | -149.6911 |
| CY089549 | A/pintail/Alaska/315/2005 (H3N8) | 8/11/05 | 64.98129 | -149.6911 |
| CY096766 | A/pintail/Alaska/246/2005 (mixed) | 8/11/05 | 64.98129 | -149.6911 |
| CY095229 | A/Muscovy duck/New York/97382-2/2005 (H1N1) | 8/12/05 | 43.205176 | -74.086304 |
| CY075934 | A/mallard/California/19524-001/2005 (H6N8) | 8/14/05 |  |  |
| CY095221 | A/Muscovy duck/New York/99159/2005 (H1N1) | 8/16/05 | 43.205176 | -74.086304 |
| CY017750 | A/mallard/Alaska/708/2005 (H8N4) | 8/19/05 | 64.98129 | -149.6911 |
| CY016412 | A/mallard/Alaska/715/2005 (H3N8) | 8/19/05 | 64.98129 | -149.6911 |
| CY095261 | A/mallard/British Columbia/07594/2005 (H5N2) | 8/19/05 | 50.17 | -120.51 |
| CY047553 | A/mallard/Quebec/16334/2005 (H5N3) | 8/19/05 | 46.15 | -72.7167 |
| CY045272 | A/mallard/Quebec/16485/2005 (H3N3) | 8/19/05 | 46.1323 | -72.9153 |
| CY045336 | A/mallard/California/GL30/2005 (H4N6) | 8/20/05 |  |  |
| CY056269 | A/mallard/California/39/2005 (H5N9) | 8/20/05 |  |  |
| CY047561 | A/mallard/Quebec/16566/2005 (H11N2) | 8/21/05 | 46.1323 | -72.9153 |
| CY047489 | A/mallard/British Columbia/07706/2005 (H3N8) | 8/22/05 | 50.0212 | -120.4109 |
| CY095316 | A/mallard/Ontario/15914/2005 (H6N2) | 8/28/05 | 42.81 | -80.95 |
| CY095292 | A/mallard/Ontario/15873/2005 (H3N2) | 8/29/05 | 42.59 | -80.46 |
| CY047497 | A/mallard/British Columbia/07826/2005 (H5N2) | 8/30/05 | 50.0212 | -120.4109 |
| CY037008 | A/blue-winged teal/Manitoba/13436/2005 (H3N8) | 8/30/05 | 50.156 | -95.4289 |
| CY095308 | A/mallard/Ontario/15741/2005 (H5N1) | 9/18/05 | 44.08 | -78.91 |
| CY047569 | A/green-winged teal/Nova Scotia/14687/2005 (H4N6) | 9/22/05 | 45.7833 | -64.25 |
| CY043809 | A/ring-necked duck/California/K90/2005 (H6N8) | 10/29/05 | 39.40443986 | -122.1736667 |
| CY045344 | A/northern shoveler/California/K138/2005 (H6N2) | 11/6/05 |  |  |
| CY053798 | A/northern shoveler/California/K168/2005 (H1N9) | 11/13/05 |  |  |
| CY045352 | A/green-winged teal/California/K218/2005 (H4N6) | 12/4/05 | 39.40443986 | -122.1736667 |
| CY017774 | A/longtail duck/Maryland/291/2005 (H3N8) | 12/5/05 | 39.076243 | -76.557541 |
| CY020910 | A/common scoter/Maryland/297/2005 (H10N8) | 12/5/05 | 39.076243 | -76.557541 |
| CY021894 | A/white-winged scoter/Maryland/301/2005 (H4N6) | 12/5/05 | 39.076243 | -76.557541 |
| CY096777 | A/common scoter/Maryland/299/2005 (mixed) | 12/5/05 | 39.076243 | -76.557541 |
| CY017790 | A/snow goose/Maryland/410/2005 (H6N1) | 12/13/05 | 39.076243 | -76.557541 |
| CY018918 | A/snow goose/Maryland/364/2005 (H6N1) | 12/13/05 | 39.076243 | -76.557541 |
| CY018910 | A/snow goose/Maryland/353/2005 (H6N1) | 12/13/05 | 39.076243 | -76.557541 |
| CY021638 | A/mallard/Delaware/418/2005 (H7N3) | 12/15/05 | 39.047 | -74.92 |
| CY053814 | A/green-winged teal/California/K481/2006 (H1N3) | 1/7/06 |  |  |
| CY053806 | A/mallard/California/K752/2006 (H10N7) | 2/4/06 |  |  |
| CY036003 | A/Muscovy duck/New York/19495-7/2006 (H7N2) | 2/21/06 | 43.205176 | -74.086304 |
| CY036035 | A/Muscovy duck/New York/62095-1/2006 (H5N2) | 5/15/06 | 43.205176 | -74.086304 |
| CY041283 | A/shorebird/Delaware/133/2006 (H6N8) | 5/22/06 | 38.85789 | -75.257721 |
| CY043889 | A/shorebird/Delaware/221/2006 (H13N9) | 5/22/06 | 39.047 | -74.92 |
| CY043897 | A/shorebird/Delaware/224/2006 (H13N9) | 5/22/06 | 39.047 | -74.92 |
| CY043905 | A/shorebird/Delaware/246/2006 (H1N1) | 5/22/06 | 39.047 | -74.92 |
| CY043913 | A/shorebird/Delaware/249/2006 (H9N2) | 5/22/06 | 39.047 | -74.92 |
| CY045384 | A/shorebird/Delaware/195/2006 (H16N3) | 5/22/06 | 39.047 | -74.92 |
| CY047020 | A/ruddy turnstone/Delaware/291/2006 (H6N1) | 5/22/06 | 39.047 | -74.92 |
| CY077119 | A/shorebird/Delaware/255/2006 (H6N1) | 5/22/06 | 39.047 | -74.92 |
| CY077127 | A/ruddy turnstone/Delaware/293/2006 (H6N2) | 5/22/06 | 39.047 | -74.92 |
| CY037080 | A/laughing gull/Delaware Bay/6/2006 (H7N3) | 5/22/06 | 39.047 | -74.92 |
| CY037088 | A/laughing gull/Delaware Bay/46/2006 (H7N3) | 5/22/06 | 39.047 | -74.92 |
| CY037104 | A/ruddy turnstone/Delaware Bay/283/2006 (H7N3) | 5/22/06 | 39.047 | -74.92 |
| CY041315 | A/ruddy turnstone/Delaware Bay/279/2006 (H7N3) | 5/22/06 | 39.047 | -74.92 |
| CY095649 | A/laughing gull/Delaware Bay/50/2006 (H7N3) | 5/22/06 | 39.047 | -74.92 |
| CY077135 | A/shorebird/Delaware/350/2006 (H1N1) | 5/23/06 | 38.85789 | -75.257721 |
| CY043921 | A/shorebird/Delaware/558/2006 (H1N1) | 5/23/06 | 39.047 | -74.92 |
| CY077143 | A/sanderling/Delaware/449/2006 (H9N2) | 5/23/06 | 39.047 | -74.92 |
| CY036768 | A/shorebird/Delaware Bay/560/2006 (H7N3) | 5/23/06 | 39.047 | -74.92 |
| HM060057 | A/thick-billed murre/Alaska/44086-095/2006 (H11N9) | 5/27/06 | 64.98129 | -149.6911 |
| HM060052 | A/thick-billed murre/Alaska/44088-059/2006 (H11N9) | 5/30/06 | 64.98129 | -149.6911 |
| HM060042 | A/thick-billed murre/Alaska/44085-090/2006 (H11N9) | 6/5/06 | 64.98129 | -149.6911 |
| HM060056 | A/thick-billed murre/Alaska/44085-108/2006 (H1N9) | 6/7/06 | 64.98129 | -149.6911 |
| HM060058 | A/thick-billed murre/Alaska/44145-199/2006 (H2N6) | 6/8/06 | 64.98129 | -149.6911 |
| CY076182 | A/mallard/Washington/44242-124/2006 (H3N2) | 7/23/06 | 48.224673 | -122.310791 |
| CY045392 | A/pintail/Alberta/21/2006 (H1N1) | 7/24/06 | 50.3 | -113.4 |
| CY077151 | A/mallard/Alberta/76/2006 (H1N3) | 7/26/06 | 53.555978 | -116.343541 |
| CY077103 | A/mallard/Alberta/128/2006 (H1N1) | 8/3/06 | 53.555978 | -116.343541 |
| CY078436 | A/green-winged teal/Interior Alaska/6MP0736/2006 (H3N8) | 8/7/06 | 64.8983 | -148.7645 |
| CY078708 | A/northern pintail/Interior Alaska/6MP0741R2/2006 (H4N6) | 8/8/06 | 64.8734 | -148.8381 |
| CY078460 | A/mallard/Interior Alaska/6MP0758/2006 (H10N8) | 8/8/06 | 64.9042 | -148.8141 |
| CY078444 | A/mallard/Interior Alaska/6MP0745/2006 (H4N6) | 8/8/06 | 64.98129 | -149.6911 |
| CY078452 | A/mallard/Interior Alaska/6MP0747/2006 (mixed) | 8/8/06 | 64.98129 | -149.6911 |
| CY077159 | A/mallard/Alberta/221/2006 (H12N6) | 8/9/06 | 53.555978 | -116.343541 |
| CY077167 | A/mallard/Alberta/224/2006 (H12N5) | 8/9/06 | 53.555978 | -116.343541 |
| CY079694 | A/mallard/Interior Alaska/6MP0017R1/2006 (mixed) | 8/9/06 | 64.9042 | -148.8141 |
| CY078876 | A/mallard/Interior Alaska/6MP0124/2006 (H3N8) | 8/10/06 | 64.8983 | -148.7645 |
| CY078580 | A/northern pintail/Interior Alaska/6MP0792/2006 (H2N3) | 8/10/06 | 64.98129 | -149.6911 |
| CY076198 | A/mallard/Washington/44242-264/2006 (H5N2) | 8/10/06 | 48.224673 | -122.310791 |
| CY078564 | A/mallard/Interior Alaska/6MP0050R1/2006 (H3N8) | 8/11/06 | 64.8983 | -148.7645 |
| CY078692 | A/mallard/Interior Alaska/6MP0038BR2/2006 (H2N3) | 8/11/06 | 64.9042 | -148.8141 |
| CY078428 | A/mallard/Interior Alaska/6MP0155/2006 (H3N8) | 8/11/06 | 64.9259 | -148.8151 |
| CY079725 | A/northern pintail/Interior Alaska/6MP0804/2006 (mixed) | 8/11/06 | 64.98129 | -149.6911 |
| CY078532 | A/northern pintail/Interior Alaska/6MP0814/2006 (H3N8) | 8/12/06 | 64.873 | -148.842 |
| CY078468 | A/northern pintail/Interior Alaska/6MP0817/2006 (mixed) | 8/12/06 | 64.8983 | -148.7645 |
| CY078700 | A/mallard/Interior Alaska/6MP0163AR2/2006 (H3N8) | 8/12/06 | 64.9042 | -148.8141 |
| CY078676 | A/mallard/Interior Alaska/6MP0160AR1/2006 (H3N8) | 8/12/06 | 64.98129 | -149.6911 |
| CY076190 | A/mallard/Washington/44242-144/2006 (H4N6) | 8/14/06 | 48.224673 | -122.310791 |
| CY078572 | A/mallard/Interior Alaska/6MP0272/2006 (H3N8) | 8/15/06 | 64.8641 | -148.7724 |
| CY095459 | A/American black duck/Prince Edward Island/14230/2006 (H3N8) | 8/15/06 | 46.33 | -62.85 |
| CY095451 | A/American black duck/Prince Edward Island/14228/2006 (H3N8) | 8/15/06 | 46.33 | -62.85 |
| CY076206 | A/mallard/Washington/44242-271/2006 (H4N6) | 8/15/06 | 48.224673 | -122.310791 |
| CY078588 | A/mallard/Interior Alaska/6MP0878/2006 (H3N8) | 8/16/06 | 64.8983 | -148.7645 |
| CY078596 | A/mallard/Interior Alaska/6MP0891/2006 (H4N6) | 8/16/06 | 64.8983 | -148.7645 |
| CY076222 | A/mallard/Washington/44242-290/2006 (H4N6) | 8/16/06 | 48.224673 | -122.310791 |
| CY078476 | A/green-winged teal/Interior Alaska/6MP0909/2006 (H3N8) | 8/17/06 | 64.8983 | -148.7645 |
| CY078484 | A/mallard/Interior Alaska/6MP0915/2006 (H3N8) | 8/17/06 | 64.8983 | -148.7645 |
| CY078652 | A/green-winged teal/Interior Alaska/6MP0936R1/2006 (H3N8) | 8/17/06 | 64.8983 | -148.7645 |
| CY078668 | A/mallard/Interior Alaska/6MP0972R1/2006 (H3N8) | 8/17/06 | 64.9152 | -148.8071 |
| CY078644 | A/mallard/Interior Alaska/6MP0935R1/2006 (H3N8) | 8/17/06 | 64.9225 | -148.7696 |
| CY078636 | A/northern pintail/Interior Alaska/6MP0964/2006 (H3N8) | 8/17/06 | 64.98129 | -149.6911 |
| CY095475 | A/mallard/British Columbia/18311/2006 (H5N2) | 8/18/06 | 50.17 | -120.51 |
| CY078660 | A/mallard/Interior Alaska/6MP0956R1/2006 (H4N6) | 8/18/06 | 64.903 | -148.745 |
| CY078492 | A/mallard/Interior Alaska/6MP0975/2006 (H3N8) | 8/18/06 | 64.98129 | -149.6911 |
| CY078604 | A/mallard/Interior Alaska/6MP0951/2006 (H3N8) | 8/18/06 | 64.98129 | -149.6911 |
| CY078612 | A/mallard/Interior Alaska/6MP0952/2006 (H3N6) | 8/18/06 | 64.98129 | -149.6911 |
| CY079741 | A/mallard/Interior Alaska/6MP0983R1/2006 (H3N8) | 8/18/06 | 64.98129 | -149.6911 |
| CY045280 | A/mallard/Quebec/10969/2006 (H2N3) | 8/18/06 | 46.13 | -72.9 |
| CY076214 | A/mallard/Washington/44242-288/2006 (H4N6) | 8/18/06 | 48.224673 | -122.310791 |
| CY078500 | A/mallard/Interior Alaska/6MP0991/2006 (H3N2) | 8/19/06 | 64.9152 | -148.8071 |
| CY078508 | A/mallard/Interior Alaska/6MP0992/2006 (H3N2) | 8/19/06 | 64.9152 | -148.8071 |
| CY078620 | A/mallard/Interior Alaska/6MP0988/2006 (mixed) | 8/19/06 | 64.9152 | -148.8071 |
| CY078884 | A/mallard/Interior Alaska/6MP0984/2006 (H7N3) | 8/19/06 | 64.9152 | -148.8071 |
| CY045288 | A/mallard/Quebec/11063/2006 (H2N3) | 8/19/06 | 46.13 | -72.92 |
| CY047585 | A/mallard/Quebec/11002/2006 (H4N6) | 8/19/06 | 46.13 | -72.92 |
| CY047593 | A/mallard/Quebec/11040/2006 (H3N2) | 8/19/06 | 46.13 | -72.92 |
| CY047601 | A/mallard/Quebec/11045/2006 (H3N2) | 8/19/06 | 46.13 | -72.92 |
| CY047609 | A/mallard/Quebec/11082/2006 (H3N8) | 8/19/06 | 46.13 | -72.92 |
| CY078540 | A/green-winged teal/Interior Alaska/6MP1077/2006 (H3N8) | 8/20/06 | 64.903 | -148.745 |
| CY078684 | A/northern pintail/Interior Alaska/6MP1080R1/2006 (H3N8) | 8/20/06 | 64.9225 | -148.7696 |
| CY045296 | A/mallard/Quebec/11111/2006 (H11N9) | 8/20/06 | 46.13 | -72.92 |
| CY047577 | A/mallard/Quebec/11102/2006 (H4N6) | 8/20/06 | 46.13 | -72.92 |
| CY047617 | A/mallard/Quebec/11103/2006 (H4N6) | 8/20/06 | 46.13 | -72.92 |
| CY047625 | A/mallard/Quebec/11106/2006 (H4N6) | 8/20/06 | 46.13 | -72.92 |
| CY095395 | A/mallard/Quebec/11093/2006 (H3N2) | 8/20/06 | 46.13 | -72.92 |
| CY047633 | A/mallard/Quebec/11121/2006 (H3N2) | 8/20/06 | 46.13 | -72.9 |
| CY076238 | A/northern pintail/Oregon/44249-559/2006 (H3N8) | 8/22/06 | 43.952293 | -121.420898 |
| CY047641 | A/mallard/Quebec/11182/2006 (H4N6) | 8/24/06 | 46.15 | -72.72 |
| CY047649 | A/American black duck/Quebec/11235/2006 (H3N2) | 8/24/06 | 46.15 | -72.72 |
| CY047657 | A/mallard/Quebec/11247/2006 (H3N2) | 8/24/06 | 46.15 | -72.72 |
| CY047673 | A/mallard/Quebec/11194/2006 (H3N2) | 8/24/06 | 46.15 | -72.72 |
| CY095379 | A/mallard/Quebec/11200/2006 (H5N2) | 8/24/06 | 46.15 | -72.72 |
| CY095371 | A/mallard/Quebec/11189/2006 (H4N6) | 8/24/06 | 46.15 | -72.72 |
| CY047665 | A/mallard/Quebec/11281/2006 (H2N3) | 8/25/06 | 46.15 | -72.72 |
| CY078516 | A/northern shoveler/Interior Alaska/6MP1287/2006 (H3N8) | 9/2/06 | 64.98129 | -149.6911 |
| CY079717 | A/northern shoveler/Interior Alaska/6MP1283/2006 (H3N6) | 9/2/06 | 64.98129 | -149.6911 |
| CY080272 | A/mallard/Interior Alaska/6MP1096R2/2006 (H4N6) | 9/2/06 | 64.98129 | -149.6911 |
| CY078524 | A/green-winged teal/Interior Alaska/6MP1312/2006 (H3N8) | 9/3/06 | 64.98129 | -149.6911 |
| JF323768 | A/Steller's eider/Alaska/44222-192/2006 (H3N3) | 9/6/06 | 64.98129 | -149.6911 |
| CY078548 | A/green-winged teal/Interior Alaska/6MP1330/2006 (H3N8) | 9/8/06 | 64.98129 | -149.6911 |
| CY079757 | A/mallard/Interior Alaska/6MP1107R1/2006 (H3N8) | 9/8/06 | 64.98129 | -149.6911 |
| CY078556 | A/northern shoveler/Interior Alaska/6MP1339/2006 (H3N8) | 9/9/06 | 64.98129 | -149.6911 |
| CY045304 | A/American black duck/New Brunswick/19347/2006 (H4N6) | 9/14/06 | 47.6167 | -65.6167 |
| CY095427 | A/American black duck/New Brunswick/19389/2006 (H4N8) | 9/17/06 | 47.62 | -65.62 |
| CY095435 | A/American black duck/New Brunswick/19392/2006 (H4N8) | 9/17/06 | 47.62 | -65.62 |
| CY095443 | A/American black duck/New Brunswick/19497/2006 (H4N6) | 9/18/06 | 47.65 | -65.62 |
| CY045312 | A/American black duck/New Brunswick/19502/2006 (H4N6) | 9/18/06 | 47.65 | -65.6167 |
| CY078628 | A/mallard/Interior Alaska/6MP1142/2006 (H3N6) | 9/22/06 | 64.98129 | -149.6911 |
| CY079733 | A/green-winged teal/Interior Alaska/6MP1140/2006 (H3N8) | 9/22/06 | 64.98129 | -149.6911 |
| HM060049 | A/glaucous gull/Alaska/44201-161/2006 (H3N8) | 9/24/06 | 64.98129 | -149.6911 |
| HM060047 | A/lesser snow goose/Alaska/44199-115/2006 (H13N9) | 9/24/06 | 64.98129 | -149.6911 |
| HM060043 | A/glaucous gull/Alaska/44199-104/2006 (H13N9) | 9/25/06 | 64.98129 | -149.6911 |
| CY045368 | A/mallard/Washington/20010-002/2006 (H3N8) | 10/1/06 | 48.224673 | -122.310791 |
| GQ168613 | A/Pacific golden plover/Alaska/44201-109/2006 (H3N8) | 10/9/06 | 64.98129 | -149.6911 |
| CY076174 | A/mallard/Oregon/44221-105/2006 (H3N6) | 10/14/06 | 43.952293 | -121.420898 |
| CY076246 | A/northern shoveler/Washington/44249-603/2006 (H6N1) | 10/26/06 | 48.224673 | -122.310791 |
| CY076254 | A/northern shoveler/Washington/44249-645/2006 (H5N2) | 11/4/06 | 48.224673 | -122.310791 |
| CY076262 | A/northern shoveler/Washington/44249-664/2006 (H7N3) | 11/7/06 | 48.224673 | -122.310791 |
| CY076270 | A/northern shoveler/Washington/44249-675/2006 (H10N2) | 11/7/06 | 48.224673 | -122.310791 |
| CY076326 | A/mallard/Washington/44256-522/2006 (H11N3) | 11/8/06 | 48.224673 | -122.310791 |
| CY076334 | A/mallard/Washington/44256-527/2006 (H11N9) | 11/8/06 | 48.224673 | -122.310791 |
| CY076278 | A/northern shoveler/Washington/44249-700/2006 (H10N1) | 11/11/06 | 48.224673 | -122.310791 |
| CY076286 | A/northern shoveler/Washington/44249-731/2006 (H10N7) | 11/30/06 | 48.224673 | -122.310791 |
| CY075958 | A/northern shoveler/California/44241-862/2006 (H11N9) | 12/2/06 | 39.40443986 | -122.1736667 |
| CY076817 | A/cinnamon teal/California/JN611/2006 (H7N3) | 12/6/06 | 39.40443986 | -122.1736667 |
| CY053790 | A/northern shoveler/California/JN587/2006 (H10N3) | 12/6/06 |  |  |
| CY076809 | A/northern shoveler/California/JN770/2006 (H10N3) | 12/6/06 |  |  |
| CY076833 | A/northern shoveler/California/JN950/2006 (H10N7) | 12/6/06 |  |  |
| CY076841 | A/bufflehead/California/JN1016/2006 (H2N9) | 12/6/06 |  |  |
| CY076294 | A/northern shoveler/Washington/44249-749/2006 (H7N3) | 12/7/06 | 48.224673 | -122.310791 |
| CY076302 | A/northern shoveler/Washington/44249-752/2006 (H7N3) | 12/7/06 | 48.224673 | -122.310791 |
| CY076310 | A/northern shoveler/Washington/44249-765/2006 (H10N2) | 12/9/06 | 48.224673 | -122.310791 |
| CY076318 | A/northern shoveler/Washington/44249-783/2006 (H7N3) | 12/14/06 | 48.224673 | -122.310791 |
| CY076849 | A/green-winged teal/California/JN1114/2006 (H4N6) | 12/30/06 |  |  |
| CY076230 | A/American green-winged teal/California/44242-906/2007 (H7N3) | 1/3/07 | 39.40443986 | -122.1736667 |
| CY076865 | A/green-winged teal/California/JN1323/2007 (H10N7) | 1/20/07 |  |  |
| CY075950 | A/double-crested cormorant/California/20119-001/2007 (H3N8) | 1/22/07 |  |  |
| CY075982 | A/northern shoveler/California/27820/2007 (H7N3) | 1/24/07 |  |  |
| CY075990 | A/northern shoveler/California/44287-088/2007 (H10N7) | 1/24/07 |  |  |
| CY075998 | A/gadwall/California/44287-137/2007 (H5N3) | 1/24/07 |  |  |
| CY076342 | A/American green-winged teal/California/44287-066/2007 (H11N9) | 1/24/07 |  |  |
| CY076350 | A/American green-winged teal/California/27790/2007 (H11N9) | 1/24/07 |  |  |
| CY076358 | A/American green-winged teal/California/44287-084/2007 (H7N3) | 1/24/07 |  |  |
| CY076366 | A/northern shoveler/California/27943/2007 (H10N7) | 1/24/07 |  |  |
| CY076374 | A/northern shoveler/California/44287-162/2007 (H10N7) | 1/24/07 |  |  |
| CY076382 | A/northern shoveler/California/44287-164/2007 (H7N7) | 1/24/07 |  |  |
| CY076390 | A/northern shoveler/California/27985/2007 (H7N6) | 1/24/07 |  |  |
| CY076398 | A/northern shoveler/California/44287-179/2007 (H7N6) | 1/24/07 |  |  |
| CY076006 | A/American green-winged teal/California/28228/2007 (H7N6) | 1/27/07 |  |  |
| CY076014 | A/cinnamon teal/California/44287-325/2007 (H3N8) | 1/27/07 |  |  |
| CY076022 | A/American green-winged teal/California/44287-395/2007 (H8N3) | 1/27/07 |  |  |
| CY076406 | A/cinnamon teal/California/44287-234/2007 (H11N9) | 1/27/07 |  |  |
| CY076414 | A/American green-winged teal/California/44287-305/2007 (H7N6) | 1/27/07 |  |  |
| CY076422 | A/northern shoveler/California/28327/2007 (H7N3) | 1/27/07 |  |  |
| CY076430 | A/northern shoveler/California/44287-364/2007 (H7N3) | 1/27/07 |  |  |
| CY076438 | A/American green-winged teal/California/44287-373/2007 (H8N4) | 1/27/07 |  |  |
| CY076446 | A/gadwall/California/29595/2007 (H10N7) | 1/28/07 |  |  |
| CY076454 | A/gadwall/California/44287-543/2007 (H10N7) | 1/28/07 |  |  |
| CY045376 | A/American green-winged teal/California/44287-713/2007 (H7N3) | 2/3/07 |  |  |
| CY047012 | A/American green-winged teal/California/28855/2007 (H7N3) | 2/3/07 |  |  |
| CY076462 | A/cinnamon teal/California/44287-659/2007 (H10N3) | 2/3/07 |  |  |
| CY076873 | A/northern shoveler/California/JN1447/2007 (H7N2) | 2/3/07 |  |  |
| CY076881 | A/cinnamon teal/California/JN1504/2007 (H5N3) | 2/3/07 |  |  |
| CY076094 | A/American coot/California/20181-006/2007 (H10N3) | 3/2/07 |  |  |
| CY035786 | A/least sandpiper/South Central Alaska/2/2007 (H4N8) | 5/9/07 | 60.4662 | -145.9675 |
| CY035794 | A/least sandpiper/South Central Alaska/3/2007 (H4N8) | 5/9/07 | 60.4662 | -145.9675 |
| CY035872 | A/least sandpiper/South Central Alaska/1/2007 (H4N8) | 5/9/07 | 60.4662 | -145.9675 |
| CY044001 | A/least sandpiper/Alaska/7KW0411/2007 (H4N8) | 5/10/07 | 60.4662 | -145.9675 |
| CY077326 | A/least sandpiper/South Central Alaska/7KW0434/2007 (H4N8) | 5/10/07 | 60.4662 | -145.9675 |
| CY079686 | A/avian/Southcentral Alaska/7KW0388R1/2007 (H4N8) | 5/10/07 | 60.4662 | -145.9675 |
| CY077111 | A/ruddy turnstone/Delaware/103/2007 (H5N1) | 5/22/07 | 39.047 | -74.92 |
| CY036776 | A/ruddy turnstone/Delaware Bay/108/2007 (H7N3) | 5/22/07 | 39.047 | -74.92 |
| CY036784 | A/ruddy turnstone/Delaware Bay/121/2007 (H7N3) | 5/22/07 | 39.047 | -74.92 |
| CY036792 | A/ruddy turnstone/Delaware Bay/123/2007 (H7N3) | 5/22/07 | 39.047 | -74.92 |
| CY045400 | A/shorebird/Delaware/189/2007 (H5N1) | 5/23/07 | 39.047 | -74.92 |
| CY077175 | A/shorebird/Delaware/554/2007 (H9N1) | 5/24/07 | 38.85789 | -75.257721 |
| CY043929 | A/shorebird/Delaware/472/2007 (H5N1) | 5/24/07 | 39.047 | -74.92 |
| CY076102 | A/common murre/Oregon/20361-001/2007 (H10N7) | 7/24/07 | 43.952293 | -121.420898 |
| CY076110 | A/common murre/Oregon/20361-002/2007 (H12N5) | 7/24/07 | 43.952293 | -121.420898 |
| CY076118 | A/California gull/Washington/20371-003/2007 (H10N7) | 7/24/07 | 48.224673 | -122.310791 |
| CY063744 | A/blue-winged teal/Minnesota/Sg-00028/2007 (H4N6) | 8/2/07 | 47.002734 | -94.251709 |
| CY064066 | A/mallard/Minnesota/Sg-00055/2007 (H12N5) | 8/2/07 | 47.002734 | -94.251709 |
| CY077183 | A/mallard/Alberta/162/2007 (H12N5) | 8/3/07 | 53.555978 | -116.343541 |
| CY077191 | A/mallard/Alberta/234/2007 (H12N5) | 8/3/07 | 53.555978 | -116.343541 |
| CY063866 | A/blue-winged teal/Minnesota/Sg-00029/2007 (H4N6) | 8/3/07 | 47.002734 | -94.251709 |
| CY063874 | A/blue-winged teal/Minnesota/Sg-00030/2007 (H4N6) | 8/3/07 | 47.002734 | -94.251709 |
| CY063882 | A/blue-winged teal/Minnesota/Sg-00031/2007 (H4N6) | 8/3/07 | 47.002734 | -94.251709 |
| CY063890 | A/blue-winged teal/Minnesota/Sg-00032/2007 (H4N6) | 8/3/07 | 47.002734 | -94.251709 |
| CY063898 | A/blue-winged teal/Minnesota/Sg-00033/2007 (mixed) | 8/3/07 | 47.002734 | -94.251709 |
| CY063906 | A/blue-winged teal/Minnesota/Sg-00034/2007 (H4N6) | 8/3/07 | 47.002734 | -94.251709 |
| CY063914 | A/blue-winged teal/Minnesota/Sg-00035/2007 (H4N6) | 8/3/07 | 47.002734 | -94.251709 |
| CY063930 | A/blue-winged teal/Minnesota/Sg-00037/2007 (H3N8) | 8/3/07 | 47.002734 | -94.251709 |
| CY063938 | A/blue-winged teal/Minnesota/Sg-00038/2007 (H4N6) | 8/3/07 | 47.002734 | -94.251709 |
| CY063946 | A/blue-winged teal/Minnesota/Sg-00039/2007 (H4N6) | 8/3/07 | 47.002734 | -94.251709 |
| CY063954 | A/blue-winged teal/Minnesota/Sg-00040/2007 (H4N6) | 8/3/07 | 47.002734 | -94.251709 |
| CY063962 | A/blue-winged teal/Minnesota/Sg-00041/2007 (mixed) | 8/3/07 | 47.002734 | -94.251709 |
| CY063970 | A/blue-winged teal/Minnesota/Sg-00042/2007 (mixed) | 8/3/07 | 47.002734 | -94.251709 |
| CY063978 | A/blue-winged teal/Minnesota/Sg-00043/2007 (H4N6) | 8/3/07 | 47.002734 | -94.251709 |
| CY063986 | A/gadwall/Minnesota/Sg-00044/2007 (mixed) | 8/3/07 | 47.002734 | -94.251709 |
| CY063994 | A/mallard/Minnesota/Sg-00045/2007 (H4N6) | 8/3/07 | 47.002734 | -94.251709 |
| CY064002 | A/mallard/Minnesota/Sg-00046/2007 (mixed) | 8/3/07 | 47.002734 | -94.251709 |
| CY064010 | A/mallard/Minnesota/Sg-00047/2007 (H3N8) | 8/3/07 | 47.002734 | -94.251709 |
| CY064018 | A/mallard/Minnesota/Sg-00048/2007 (H3N8) | 8/3/07 | 47.002734 | -94.251709 |
| CY064026 | A/mallard/Minnesota/Sg-00049/2007 (mixed) | 8/3/07 | 47.002734 | -94.251709 |
| CY064034 | A/mallard/Minnesota/Sg-00050/2007 (H4N6) | 8/3/07 | 47.002734 | -94.251709 |
| CY064042 | A/mallard/Minnesota/Sg-00051/2007 (mixed) | 8/3/07 | 47.002734 | -94.251709 |
| CY064050 | A/mallard/Minnesota/Sg-00052/2007 (H4N6) | 8/3/07 | 47.002734 | -94.251709 |
| CY064058 | A/mallard/Minnesota/Sg-00053/2007 (H4N6) | 8/3/07 | 47.002734 | -94.251709 |
| CY064074 | A/mallard/Minnesota/Sg-00056/2007 (H10N7) | 8/3/07 | 47.002734 | -94.251709 |
| CY064082 | A/mallard/Minnesota/Sg-00057/2007 (H10N7) | 8/3/07 | 47.002734 | -94.251709 |
| CY064090 | A/mallard/Minnesota/Sg-00058/2007 (H4N6) | 8/3/07 | 47.002734 | -94.251709 |
| CY064098 | A/mallard/Minnesota/Sg-00059/2007 (H3N8) | 8/3/07 | 47.002734 | -94.251709 |
| CY064106 | A/mallard/Minnesota/Sg-00060/2007 (mixed) | 8/3/07 | 47.002734 | -94.251709 |
| CY064114 | A/mallard/Minnesota/Sg-00061/2007 (H3N8) | 8/3/07 | 47.002734 | -94.251709 |
| CY064122 | A/mallard/Minnesota/Sg-00062/2007 (H8N4) | 8/3/07 | 47.002734 | -94.251709 |
| CY064130 | A/mallard/Minnesota/Sg-00063/2007 (H4N6) | 8/3/07 | 47.002734 | -94.251709 |
| CY064138 | A/mallard/Minnesota/Sg-00064/2007 (H3N8) | 8/3/07 | 47.002734 | -94.251709 |
| CY064146 | A/mallard/Minnesota/Sg-00065/2007 (H10N7) | 8/3/07 | 47.002734 | -94.251709 |
| CY064154 | A/ring-necked duck/Minnesota/Sg-00066/2007 (mixed) | 8/3/07 | 47.002734 | -94.251709 |
| CY064162 | A/ring-necked duck/Minnesota/Sg-00067/2007 (H4N6) | 8/3/07 | 47.002734 | -94.251709 |
| CY064170 | A/ring-necked duck/Minnesota/Sg-00068/2007 (H10N7) | 8/3/07 | 47.002734 | -94.251709 |
| CY064178 | A/ring-necked duck/Minnesota/Sg-00069/2007 (mixed) | 8/3/07 | 47.002734 | -94.251709 |
| CY063922 | A/blue-winged teal/Minnesota/Sg-00036/2007 (H4N6) | 8/3/07 | 47.002734 | -94.251709 |
| CY076126 | A/mallard/California/20385-002/2007 (H1N1) | 8/5/07 |  |  |
| CY076134 | A/mallard/California/20385-004/2007 (H1N1) | 8/5/07 |  |  |
| CY043945 | A/northern pintail/Alaska/7MP0608/2007 (H3N8) | 8/6/07 | 64.914 | -148.809 |
| CY039781 | A/northern pintail/Interior Alaska/1/2007 (H3N8) | 8/7/07 | 64.878 | -148.824 |
| CY036654 | A/northern pintail/Interior Alaska/1/2007 (H4N6) | 8/8/07 | 64.873 | -148.842 |
| CY077215 | A/northern pintail/Interior Alaska/7MP1246/2007 (H4N6) | 8/8/07 | 64.873 | -148.842 |
| CY077318 | A/northern pintail/Interior Alaska/7MP0692/2007 (H1N1) | 8/8/07 | 64.903 | -148.816 |
| CY045432 | A/mallard/Interior Alaska/7MP0709/2007 (H3N8) | 8/9/07 | 64.924 | -148.817 |
| CY078956 | A/mallard/Interior Alaska/7MP0747/2007 (H1N1) | 8/10/07 | 64.878 | -148.824 |
| CY045320 | A/northern pintail/Saskatchewan/22910/2007 (H3N2) | 8/10/07 | 51.83 | -104.9119 |
| CY039750 | A/northern pintail/Interior Alaska/1/2007 (H1N1) | 8/11/07 | 64.898 | -148.765 |
| CY036662 | A/green-winged teal/Interior Alaska/1/2007 (H3N8) | 8/11/07 | 64.903 | -148.816 |
| CY077199 | A/mallard/Interior Alaska/7MP0167/2007 (H12N5) | 8/13/07 | 64.898 | -148.765 |
| CY078932 | A/mallard/Interior Alaska/7MP0172/2007 (H1N1) | 8/13/07 | 64.905 | -148.746 |
| CY043993 | A/northern pintail/Alaska/7MP1393/2007 (H4N6) | 8/14/07 | 64.873 | -148.842 |
| CY043937 | A/northern pintail/Alaska/7MP0344/2007 (H3N8) | 8/16/07 | 64.873 | -148.842 |
| CY045408 | A/northern pintail/Interior Alaska/7MP0343/2007 (H3N8) | 8/16/07 | 64.873 | -148.842 |
| CY078900 | A/northern pintail/Interior Alaska/7MP0345BR2/2007 (H3N8) | 8/16/07 | 64.873 | -148.842 |
| CY079044 | A/northern pintail/Interior Alaska/7MP1460R2/2007 (H3N8) | 8/16/07 | 64.873 | -148.842 |
| CY076030 | A/teal/Oregon/44336-122/2007 (H3N8) | 8/16/07 | 43.952293 | -121.420898 |
| CY076038 | A/American green-winged teal/Oregon/44336-124/2007 (H3N8) | 8/16/07 | 43.952293 | -121.420898 |
| CY076046 | A/teal/Oregon/44336-130/2007 (H3N8) | 8/16/07 | 43.952293 | -121.420898 |
| CY045416 | A/mallard/Interior Alaska/7MP0372/2007 (H4N6) | 8/17/07 | 64.898 | -148.765 |
| CY077311 | A/northern pintail/Interior Alaska/7MP0278/2007 (H1N1) | 8/17/07 | 64.873 | -148.842 |
| CY039758 | A/mallard/Interior Alaska/6/2007 (H3N8) | 8/17/07 | 64.924 | -148.817 |
| CY078940 | A/northern pintail/Interior Alaska/7MP0408/2007 (H3N8) | 8/18/07 | 64.873 | -148.842 |
| CY047705 | A/blue-winged teal/Saskatchewan/22542/2007 (H5N2) | 8/18/07 | 51.315 | -105.2866 |
| CY047681 | A/American green-winged teal/Manitoba/23884/2007 (H4N6) | 8/21/07 | 50.154 | -98.431 |
| CY047689 | A/mallard/Manitoba/23912/2007 (H4N7) | 8/24/07 | 50.472 | -98.862 |
| CY076486 | A/mallard/Washington/44338-009/2007 (H6N1) | 8/29/07 | 48.224673 | -122.310791 |
| CY076494 | A/mallard/Washington/44338-011/2007 (H4N6) | 8/29/07 | 48.224673 | -122.310791 |
| CY076502 | A/mallard/Washington/44338-012/2007 (H4N6) | 8/29/07 | 48.224673 | -122.310791 |
| CY076518 | A/mallard/Washington/44338-015/2007 (H4N6) | 8/29/07 | 48.224673 | -122.310791 |
| CY076526 | A/mallard/Washington/44338-016/2007 (H4N6) | 8/29/07 | 48.224673 | -122.310791 |
| CY076534 | A/mallard/Washington/44338-017/2007 (H4N6) | 8/29/07 | 48.224673 | -122.310791 |
| CY076542 | A/mallard/Washington/44338-018/2007 (H4N6) | 8/29/07 | 48.224673 | -122.310791 |
| CY076550 | A/mallard/Washington/44338-019/2007 (H6N1) | 8/29/07 | 48.224673 | -122.310791 |
| CY076558 | A/mallard/Washington/44338-029/2007 (H6N1) | 8/29/07 | 48.224673 | -122.310791 |
| CY076510 | A/mallard/Washington/44338-014/2007 (H4N6) | 8/29/07 | 48.224673 | -122.310791 |
| CY039837 | A/northern shoveler/Interior Alaska/1/2007 (H4N6) | 9/1/07 | 64.98129 | -149.6911 |
| CY078948 | A/duck/Interior Alaska/7MP1591R1/2007 (H3N8) | 9/1/07 | 64.98129 | -149.6911 |
| CY078980 | A/duck/Interior Alaska/7MP1570/2007 (H4N6) | 9/1/07 | 64.98129 | -149.6911 |
| CY078988 | A/duck/Interior Alaska/7MP1582/2007 (H1N1) | 9/1/07 | 64.98129 | -149.6911 |
| CY078996 | A/duck/Interior Alaska/7MP1598/2007 (H3N8) | 9/1/07 | 64.98129 | -149.6911 |
| CY080192 | A/duck/Interior Alaska/7MP1550/2007 (H4N6) | 9/1/07 | 64.98129 | -149.6911 |
| CY077342 | A/northern shoveler/Alaska/7MP1669B/2007 (H3N8) | 9/2/07 | 64.98129 | -149.6911 |
| CY077239 | A/American green-winged teal/Interior Alaska/7MP1651/2007 (H3N8) | 9/2/07 | 64.98129 | -149.6911 |
| CY077334 | A/northern shoveler/Interior Alaska/7MP1649/2007 (H3N8) | 9/2/07 | 64.98129 | -149.6911 |
| CY077365 | A/northern pintail/Interior Alaska/7MP1644/2007 (H4N6) | 9/2/07 | 64.98129 | -149.6911 |
| CY077413 | A/northern shoveler/Interior Alaska/7MP1670/2007 (H3N8) | 9/2/07 | 64.98129 | -149.6911 |
| CY079036 | A/green-winged teal/Interior Alaska/7MP1672R1/2007 (mixed) | 9/2/07 | 64.98129 | -149.6911 |
| CY079551 | A/mallard/Interior Alaska/7MP1643/2007 (mixed) | 9/2/07 | 64.98129 | -149.6911 |
| CY079909 | A/northern shoveler/Interior Alaska/7MP1667/2007 (H3N8) | 9/2/07 | 64.98129 | -149.6911 |
| CY045440 | A/northern shoveler/Alaska/7MP1708/2007 (H3N8) | 9/3/07 | 64.98129 | -149.6911 |
| CY039845 | A/mallard/Interior Alaska/1/2007 (H4N5) | 9/3/07 | 64.98129 | -149.6911 |
| CY047036 | A/mallard/Interior Alaska/7MP1757/2007 (H3N8) | 9/3/07 | 64.98129 | -149.6911 |
| CY077357 | A/American widgeon/Interior Alaska/7MP1707/2007 (H3N8) | 9/3/07 | 64.98129 | -149.6911 |
| CY078916 | A/American wigeon/Interior Alaska/7MP1726/2007 (H3N8) | 9/3/07 | 64.98129 | -149.6911 |
| CY079204 | A/mallard/Interior Alaska/7MP1701/2007 (mixed) | 9/3/07 | 64.98129 | -149.6911 |
| CY044009 | A/northern shoveler/Alaska/7MP1606/2007 (H3N8) | 9/4/07 | 64.98129 | -149.6911 |
| CY077271 | A/northern shoveler/Interior Alaska/7MP1765/2007 (H3N8) | 9/4/07 | 64.98129 | -149.6911 |
| CY080200 | A/northern shoveler/Interior Alaska/7MP1766/2007 (H3N8) | 9/4/07 | 64.98129 | -149.6911 |
| CY076566 | A/mallard/Washington/44338-034/2007 (H6N1) | 9/4/07 | 48.224673 | -122.310791 |
| CY076574 | A/mallard/Washington/44338-037/2007 (H6N1) | 9/4/07 | 48.224673 | -122.310791 |
| CY076582 | A/mallard/Washington/44338-039/2007 (H6N1) | 9/4/07 | 48.224673 | -122.310791 |
| CY076590 | A/mallard/Washington/44338-045/2007 (H6N1) | 9/4/07 | 48.224673 | -122.310791 |
| CY076598 | A/mallard/Washington/44338-047/2007 (H6N1) | 9/4/07 | 48.224673 | -122.310791 |
| CY076606 | A/mallard/Washington/44338-049/2007 (H6N1) | 9/4/07 | 48.224673 | -122.310791 |
| CY039853 | A/mallard/Interior Alaska/7/2007 (H3N8) | 9/5/07 | 64.98129 | -149.6911 |
| CY077373 | A/mallard/Interior Alaska/7MP1700/2007 (H3N8) | 9/5/07 | 64.98129 | -149.6911 |
| CY078892 | A/mallard/Interior Alaska/7MP1771R1/2007 (H4N6) | 9/5/07 | 64.98129 | -149.6911 |
| CY078924 | A/northern shoveler/Interior Alaska/7MP1796/2007 (mixed) | 9/5/07 | 64.98129 | -149.6911 |
| CY079004 | A/northern pintail/Interior Alaska/7MP1822/2007 (H3N8) | 9/5/07 | 64.98129 | -149.6911 |
| CY079012 | A/mallard/Interior Alaska/7MP1835/2007 (H3N8) | 9/5/07 | 64.98129 | -149.6911 |
| CY079028 | A/northern shoveler/Interior Alaska/7MP1800/2007 (mixed) | 9/5/07 | 64.98129 | -149.6911 |
| CY039829 | A/northern shoveler/Interior Alaska/4/2007 (H3N8) | 9/5/07 | 64.98129 | -149.6911 |
| CY044017 | A/northern shoveler/Alaska/7MP0954/2007 (H4N6) | 9/6/07 | 64.98129 | -149.6911 |
| CY035802 | A/mallard/Interior Alaska/2/2007 (H3N8) | 9/6/07 | 64.98129 | -149.6911 |
| CY035810 | A/American widgeon/Interior Alaska/1/2007 (H3N8) | 9/6/07 | 64.98129 | -149.6911 |
| CY035818 | A/northern shoveler/Interior Alaska/1/2007 (H3N8) | 9/6/07 | 64.98129 | -149.6911 |
| CY035880 | A/mallard/Interior Alaska/1/2007 (H3N8) | 9/6/07 | 64.98129 | -149.6911 |
| CY039766 | A/northern shoveler/Interior Alaska/2/2007 (H3N8) | 9/6/07 | 64.98129 | -149.6911 |
| CY078908 | A/northern pintail/Interior Alaska/7MP0509/2007 (H3N8) | 9/6/07 | 64.98129 | -149.6911 |
| CY095483 | A/mallard/Ontario/26078/2007 (H5N1) | 9/6/07 | 42.39 | -82.41 |
| CY043961 | A/northern shoveler/Alaska/7MP1026/2007 (H3N8) | 9/7/07 | 64.98129 | -149.6911 |
| CY043969 | A/mallard/Alaska/7MP1028/2007 (H4N8) | 9/7/07 | 64.98129 | -149.6911 |
| CY044025 | A/northern shoveler/Alaska/7MP1668/2007 (H3N8) | 9/7/07 | 64.98129 | -149.6911 |
| CY078964 | A/northern shoveler/Interior Alaska/7MP0953/2007 (H3N8) | 9/7/07 | 64.98129 | -149.6911 |
| CY038365 | A/mallard/Interior Alaska/3/2007 (H3N8) | 9/7/07 | 64.98129 | -149.6911 |
| CY038373 | A/northern pintail/Interior Alaska/2/2007 (H4N8) | 9/7/07 | 64.98129 | -149.6911 |
| CY039789 | A/mallard/Interior Alaska/4/2007 (H4N6) | 9/7/07 | 64.98129 | -149.6911 |
| CY039797 | A/northern shoveler/Interior Alaska/3/2007 (H3N8) | 9/7/07 | 64.98129 | -149.6911 |
| CY039805 | A/American green-winged teal/Interior Alaska/4/2007 (H3N8) | 9/7/07 | 64.98129 | -149.6911 |
| CY077231 | A/mallard/Interior Alaska/7MP0510/2007 (H3N6) | 9/7/07 | 64.98129 | -149.6911 |
| CY077263 | A/mallard/Interior Alaska/7MP0512/2007 (H4N6) | 9/7/07 | 64.98129 | -149.6911 |
| CY077279 | A/northern shoveler/Interior Alaska/7MP0944/2007 (H3N8) | 9/7/07 | 64.98129 | -149.6911 |
| CY078972 | A/mallard/Interior Alaska/7MP0956/2007 (mixed) | 9/7/07 | 64.98129 | -149.6911 |
| CY079300 | A/mallard/Interior Alaska/7MP0901R1/2007 (H4N6) | 9/8/07 | 64.98129 | -149.6911 |
| CY045448 | A/northern pintail/Alaska/7MP0508/2007 (H3N8) | 9/9/07 | 64.98129 | -149.6911 |
| CY043977 | A/American widgeon/Alaska/7MP1061/2007 (H3N8) | 9/10/07 | 64.98129 | -149.6911 |
| CY043985 | A/northern pintail/Alaska/7MP1092/2007 (H3N8) | 9/10/07 | 64.98129 | -149.6911 |
| CY039813 | A/mallard/Interior Alaska/2/2007 (H4N6) | 9/10/07 | 64.98129 | -149.6911 |
| CY039821 | A/mallard/Interior Alaska/5/2007 (H3N8) | 9/10/07 | 64.98129 | -149.6911 |
| CY045424 | A/mallard/Interior Alaska/7MP1050R1/2007 (H4N6) | 9/10/07 | 64.98129 | -149.6911 |
| CY077223 | A/northern shoveler/Interior Alaska/7MP1081/2007 (H1N1) | 9/10/07 | 64.98129 | -149.6911 |
| CY077287 | A/mallard/Interior Alaska/7MP1056/2007 (H3N8) | 9/10/07 | 64.98129 | -149.6911 |
| CY077295 | A/northern shoveler/Interior Alaska/7MP1077/2007 (H1N1) | 9/10/07 | 64.98129 | -149.6911 |
| CY077303 | A/northern shoveler/Interior Alaska/7MP1080/2007 (H3N8) | 9/10/07 | 64.98129 | -149.6911 |
| CY077381 | A/American green-winged teal/Interior Alaska/7MP1049/2007 (H3N8) | 9/10/07 | 64.98129 | -149.6911 |
| CY076470 | A/northern shoveler/Oregon/44336-179/2007 (H4N6) | 9/10/07 | 43.952293 | -121.420898 |
| CY076478 | A/American green-winged teal/Oregon/44336-183/2007 (H3N8) | 9/10/07 | 43.952293 | -121.420898 |
| CY076622 | A/mallard/Washington/44338-112/2007 (H6N2) | 9/10/07 | 48.224673 | -122.310791 |
| CY076630 | A/mallard/Washington/44338-120/2007 (H6N2) | 9/10/07 | 48.224673 | -122.310791 |
| CY043953 | A/northern shoveler/Alaska/7MP1113/2007 (H4N6) | 9/11/07 | 64.98129 | -149.6911 |
| CY045456 | A/northern shoveler/Interior Alaska/7MP1601/2007 (H4N6) | 9/11/07 | 64.98129 | -149.6911 |
| CY077389 | A/northern shoveler/Interior Alaska/7MP1033/2007 (H3N8) | 9/12/07 | 64.98129 | -149.6911 |
| CY076614 | A/mallard/Washington/44338-052/2007 (H3N1) | 9/12/07 | 48.224673 | -122.310791 |
| CY077397 | A/mallard/Interior Alaska/7MP1094/2007 (H3N8) | 9/13/07 | 64.98129 | -149.6911 |
| JF323775 | A/Steller's eider/Alaska/44354-356/2007 (H3N6) | 9/14/07 | 64.98129 | -149.6911 |
| CY078107 | A/mallard/Minnesota/Sg-00164/2007 (H3N8) | 9/14/07 | 47.002734 | -94.251709 |
| CY078115 | A/mallard/Minnesota/Sg-00167/2007 (H6N1) | 9/14/07 | 47.002734 | -94.251709 |
| CY078298 | A/green-winged teal/Minnesota/Sg-00180/2007 (H6N2) | 9/14/07 | 47.002734 | -94.251709 |
| CY078314 | A/green-winged teal/Minnesota/Sg-00199/2007 (H6N2) | 9/14/07 | 47.002734 | -94.251709 |
| CY078338 | A/mallard/Minnesota/Sg-00220/2007 (H6N1) | 9/14/07 | 47.002734 | -94.251709 |
| CY077405 | A/northern pintail/Interior Alaska/7MP1067R1/2007 (H4N5) | 9/15/07 | 64.98129 | -149.6911 |
| CY078354 | A/blue-winged teal/Louisiana/Sg-00224/2007 (H3N8) | 9/15/07 | 31.449753 | -92.084656 |
| CY064186 | A/blue-winged teal/Louisiana/Sg-00073/2007 (H10N7) | 9/15/07 | 31.449753 | -92.084656 |
| CY078266 | A/green-winged teal/Louisiana/Sg-00092/2007 (H3N8) | 9/15/07 | 31.449753 | -92.084656 |
| CY064210 | A/blue-winged teal/Texas/Sg-00077/2007 (H4N6) | 9/15/07 | 31.872893 | -99.036255 |
| CY064218 | A/blue-winged teal/Texas/Sg-00078/2007 (H3N8) | 9/15/07 | 31.872893 | -99.036255 |
| CY064234 | A/blue-winged teal/Texas/Sg-00080/2007 (H4N6) | 9/15/07 | 31.872893 | -99.036255 |
| CY064242 | A/blue-winged teal/Texas/Sg-00081/2007 (H4N6) | 9/15/07 | 31.872893 | -99.036255 |
| CY077702 | A/blue-winged teal/Texas/Sg-00074/2007 (H4N8) | 9/15/07 | 31.872893 | -99.036255 |
| CY078067 | A/blue-winged teal/Texas/Sg-00155/2007 (H4N8) | 9/15/07 | 31.872893 | -99.036255 |
| CY078075 | A/blue-winged teal/Texas/Sg-00157/2007 (H4N6) | 9/15/07 | 31.872893 | -99.036255 |
| CY078187 | A/blue-winged teal/Texas/Sg-00207/2007 (H4N6) | 9/15/07 | 31.872893 | -99.036255 |
| CY078203 | A/blue-winged teal/Texas/Sg-00212/2007 (mixed) | 9/15/07 | 31.872893 | -99.036255 |
| CY078282 | A/blue-winged teal/Texas/Sg-00158/2007 (H4N6) | 9/15/07 | 31.872893 | -99.036255 |
| CY078306 | A/blue-winged teal/Texas/Sg-00188/2007 (H4N8) | 9/15/07 | 31.872893 | -99.036255 |
| CY078330 | A/blue-winged teal/Texas/Sg-00206/2007 (H4N6) | 9/15/07 | 31.872893 | -99.036255 |
| CY064226 | A/blue-winged teal/Texas/Sg-00079/2007 (H3N8) | 9/15/07 | 31.872893 | -99.036255 |
| CY039869 | A/mallard/Interior Alaska/3/2007 (H4N6) | 9/16/07 | 64.98129 | -149.6911 |
| CY045464 | A/mallard/Interior Alaska/7MP1718/2007 (H4N5) | 9/16/07 | 64.98129 | -149.6911 |
| CY047028 | A/American green-winged teal/Interior Alaska/7MP2225/2007 (H3N8) | 9/16/07 | 64.98129 | -149.6911 |
| CY077255 | A/mallard/Interior Alaska/7MP2230/2007 (H1N6) | 9/16/07 | 64.98129 | -149.6911 |
| CY079020 | A/northern pintail/Interior Alaska/7MP2228/2007 (H3N8) | 9/16/07 | 64.98129 | -149.6911 |
| CY078091 | A/mallard/Minnesota/Sg-00161/2007 (H3N8) | 9/16/07 | 47.002734 | -94.251709 |
| CY078226 | A/mallard/Minnesota/Sg-00217/2007 (H1N1) | 9/16/07 | 47.002734 | -94.251709 |
| CY078274 | A/mallard/Minnesota/Sg-00106/2007 (H6N2) | 9/16/07 | 47.002734 | -94.251709 |
| CY078346 | A/green-winged teal/Minnesota/Sg-00222/2007 (H6N2) | 9/16/07 | 47.002734 | -94.251709 |
| CY064194 | A/blue-winged teal/Texas/Sg-00075/2007 (mixed) | 9/16/07 | 31.872893 | -99.036255 |
| CY064202 | A/blue-winged teal/Texas/Sg-00076/2007 (H4N6) | 9/16/07 | 31.872893 | -99.036255 |
| CY078195 | A/blue-winged teal/Texas/Sg-00208/2007 (mixed) | 9/16/07 | 31.872893 | -99.036255 |
| CY078258 | A/blue-winged teal/Texas/Sg-00085/2007 (H3N6) | 9/16/07 | 31.872893 | -99.036255 |
| CY090854 | A/blue-winged teal/Texas/Sg-00173/2007 (H4N8) | 9/16/07 | 31.872893 | -99.036255 |
| CY078083 | A/blue-winged teal/Texas/Sg-00159/2007 (H4N8) | 9/16/07 | 31.872893 | -99.036255 |
| CY079559 | A/northern pintail/Interior Alaska/7MP2248/2007 (mixed) | 9/17/07 | 64.98129 | -149.6911 |
| CY041858 | A/mallard/South Dakota/Sg-00125/2007 (H3N2) | 9/19/07 | 44.715514 | -100.59082 |
| CY041882 | A/mallard/South Dakota/Sg-00128/2007 (H3N2) | 9/19/07 | 44.715514 | -100.59082 |
| CY041866 | A/northern pintail/South Dakota/Sg-00126/2007 (H3N2) | 9/19/07 | 44.715514 | -100.59082 |
| CY041874 | A/mallard/South Dakota/Sg-00127/2007 (H3N2) | 9/19/07 | 44.715514 | -100.59082 |
| CY047697 | A/American black duck/New Brunswick/25182/2007 (H3N6) | 9/21/07 | 47.6167 | -65.6167 |
| CY078163 | A/mallard/Minnesota/Sg-00200/2007 (H3N8) | 9/22/07 | 47.002734 | -94.251709 |
| CY078290 | A/mallard/Minnesota/Sg-00166/2007 (H6N2) | 9/22/07 | 47.002734 | -94.251709 |
| CY080288 | A/mallard/Minnesota/Sg-00195/2007 (H10N3) | 9/22/07 | 47.002734 | -94.251709 |
| CY041898 | A/mallard/Minnesota/Sg-00133/2007 (H4N6) | 9/29/07 | 47.002734 | -94.251709 |
| CY078322 | A/blue-winged teal/Minnesota/Sg-00201/2007 (H4N6) | 9/29/07 | 47.002734 | -94.251709 |
| CY041890 | A/green-winged teal/Minnesota/Sg-00131/2007 (H3N2) | 9/29/07 | 47.002734 | -94.251709 |
| CY032707 | A/American wigeon/California/HKWF371/2007 (H6N5) | 10/7/07 | 39.40443986 | -122.1736667 |
| CY038389 | A/American green-winged teal/Interior Alaska/3/2007 (H3N8) | 10/17/07 | 64.98129 | -149.6911 |
| CY076678 | A/American green-winged teal/California/44363-002/2007 (H11N5) | 10/20/07 |  |  |
| CY033423 | A/American wigeon/California/HKWF42/2007 (H6N1) | 10/21/07 |  |  |
| CY094422 | A/American wigeon/California/HKWF041C/2007 (H6N1) | 10/21/07 |  |  |
| CY032667 | A/gadwall/California/HKWF100/2007 (H6N1) | 10/24/07 |  |  |
| CY032887 | A/northern shoveler/California/HKWF115/2007 (H6N1) | 10/24/07 |  |  |
| CY035852 | A/northern pintail/California/HKWF151/2007 (H6N1) | 10/24/07 |  |  |
| CY039575 | A/northern shoveler/California/HKWF96/2007 (H10N7) | 10/24/07 |  |  |
| CY076078 | A/greater white-fronted goose/California/44358-077/2007 (H6N1) | 10/27/07 |  |  |
| CY076646 | A/greater white-fronted goose/California/44358-076/2007 (H6N1) | 10/27/07 |  |  |
| CY076654 | A/greater white-fronted goose/California/44358-089/2007 (H6N1) | 10/27/07 |  |  |
| CY076662 | A/greater white-fronted goose/California/44358-095/2007 (H6N1) | 10/27/07 |  |  |
| CY032699 | A/northern shoveler/California/HKWF268/2007 (H6N2) | 10/28/07 |  |  |
| CY032895 | A/bufflehead/California/HKWF205/2007 (H4N8) | 10/28/07 |  |  |
| CY035860 | A/northern shoveler/California/HKWF216/2007 (H6N1) | 10/28/07 |  |  |
| CY032903 | A/American wigeon/California/HKWF295/2007 (H6N5) | 10/31/07 |  |  |
| CY093855 | A/American wigeon/California/HKWF296C/2007 (H6N1) | 10/31/07 |  |  |
| CY032659 | A/northern shoveler/California/HKWF1005/2007 (H10N3) | 11/2/07 |  |  |
| CY032715 | A/northern shoveler/California/HKWF383/2007 (H6N1) | 11/3/07 |  |  |
| CY033399 | A/American wigeon/California/HKWF353/2007 (H6N1) | 11/3/07 |  |  |
| CY033407 | A/ring necked duck/California/HKWF402/2007 (H6N1) | 11/3/07 |  |  |
| CY039607 | A/northern shoveler/California/HKWF392sm/2007 (H10N7) | 11/3/07 |  |  |
| CY076086 | A/northern shoveler/California/44363-062/2007 (H9N2) | 11/3/07 |  |  |
| CY076686 | A/American green-winged teal/California/44363-067/2007 (H11N9) | 11/3/07 |  |  |
| CY032911 | A/American wigeon/California/HKWF450/2007 (H4N7) | 11/4/07 |  |  |
| CY034168 | A/greater white fronted goose/California/HKWF446/2007 (H10N7) | 11/4/07 |  |  |
| CY076670 | A/greater white-fronted goose/California/44358-112/2007 (H6N1) | 11/4/07 |  |  |
| CY076694 | A/northern shoveler/California/44363-082/2007 (H11N9) | 11/4/07 |  |  |
| CY094006 | A/northern pintail/California/HKWF440C/2007 (H6N1) | 11/4/07 |  |  |
| CY094430 | A/American wigeon/California/HKWF541C/2007 (H6N5) | 11/4/07 |  |  |
| CY094502 | A/greater white-fronted goose/California/HKWF446C/2007 (H10N7) | 11/4/07 |  |  |
| CY076142 | A/rock dove/Oregon/20547-003/2007 (H1N2) | 11/5/07 | 43.952293 | -121.420898 |
| CY076150 | A/rock dove/Oregon/20547-004/2007 (H1N2) | 11/5/07 | 43.952293 | -121.420898 |
| CY038357 | A/northern shoveler/Interior Alaska/1/2007 (H12N5) | 11/6/07 | 64.98129 | -149.6911 |
| CY033431 | A/ring necked duck/California/HKWF515/2007 (H6N1) | 11/7/07 |  |  |
| CY033439 | A/American wigeon/California/HKWF541/2007 (H6N5) | 11/7/07 |  |  |
| CY032919 | A/northern shoveler/California/HKWF611/2007 (H11N9) | 11/10/07 |  |  |
| CY033447 | A/American green-winged teal/California/HKWF609/2007 (H5N2) | 11/10/07 |  |  |
| CY039623 | A/northern shoveler/California/HKWF569/2007 (H3N1) | 11/10/07 |  |  |
| CY039631 | A/northern shoveler/California/HKWF608/2007 (H10N7) | 11/10/07 |  |  |
| CY094350 | A/northern shoveler/California/HKWF592C/2007 (H10N7) | 11/10/07 |  |  |
| CY032927 | A/ring-necked duck/California/HKWF662/2007 (H6N1) | 11/11/07 |  |  |
| CY076638 | A/mallard/Washington/44338-218/2007 (H1N1) | 11/15/07 | 48.224673 | -122.310791 |
| CY076062 | A/mallard/Washington/44338-195/2007 (H12N5) | 11/20/07 | 48.224673 | -122.310791 |
| CY076158 | A/western grebe/Washington/20569-004/2007 (H1N2) | 11/26/07 | 48.224673 | -122.310791 |
| CY032723 | A/northern pintail/California/HKWF792/2007 (H3N8) | 11/28/07 |  |  |
| CY039551 | A/northern shoveler/California/AKS273/2007 (H8N4) | 12/1/07 |  |  |
| CY032731 | A/northern shoveler/California/HKWF979/2007 (H3N3) | 12/2/07 |  |  |
| CY033327 | A/northern shoveler/California/HKWF1021/2007 (H3N7) | 12/2/07 |  |  |
| CY033335 | A/northern shoveler/California/HKWF1046/2007 (H3N5) | 12/2/07 |  |  |
| CY033351 | A/northern shoveler/California/HKWF1131/2007 (H3N5) | 12/2/07 |  |  |
| CY039583 | A/northern shoveler/California/HKWF1026/2007 (H7N3) | 12/2/07 |  |  |
| CY094358 | A/northern shoveler/California/HKWF1046C/2007 (H3N5) | 12/2/07 |  |  |
| CY094366 | A/northern shoveler/California/HKWF1128C/2007 (H2N7) | 12/2/07 |  |  |
| CY094438 | A/cinnamon teal/California/HKWF1111C/2007 (H5N7) | 12/2/07 |  |  |
| CY038381 | A/mallard/Interior Alaska/4/2007 (H3N8) | 12/2/07 | 64.98129 | -149.6911 |
| CY032675 | A/northern shoveler/California/HKWF1201/2007 (H3N5) | 12/5/07 |  |  |
| CY033359 | A/American wigeon/California/HKWF1174/2007 (H6N1) | 12/5/07 |  |  |
| CY033367 | A/northern shoveler/California/HKWF1199/2007 (H3N5) | 12/5/07 |  |  |
| CY034154 | A/northern shoveler/California/HKWF1203/2007 (H8N4) | 12/5/07 |  |  |
| CY039591 | A/northern shoveler/California/HKWF1204/2007 (H8N4) | 12/5/07 |  |  |
| CY033343 | A/northern shoveler/California/HKWF1128/2007 (H2N7) | 12/7/07 |  |  |
| CY094262 | A/mallard/California/AKS478/2007 (H10N7) | 12/8/07 |  |  |
| CY034161 | A/northern shoveler/California/HKWF1325/2007 (H8N4) | 12/9/07 |  |  |
| CY093950 | A/gadwall/California/AKS-514/2007 (H6N1) | 12/9/07 |  |  |
| CY032691 | A/northern shoveler/California/HKWF1370/2007 (H10N3) | 12/12/07 |  |  |
| CY039567 | A/greater white-fronted goose/California/AKS617/2007 (H6N1) | 12/12/07 |  |  |
| CY094374 | A/northern shoveler/California/HKWF1372C/2007 (H7N3) | 12/12/07 |  |  |
| CY076166 | A/American coot/Oregon/20589-007/2007 (H3N8) | 12/17/07 | 43.952293 | -121.420898 |
| CY039599 | A/northern shoveler/California/HKWF2031/2008 (H7N3) | 1/23/08 |  |  |
| CY039535 | A/green winged teal/California/AKS1305/2008 (H11N9) | 1/26/08 |  |  |
| CY039543 | A/green winged teal/California/AKS1370/2008 (H7N3) | 1/27/08 |  |  |
| CY080131 | A/northern pintail/Interior Alaska/8MP0213R2/2008 (H8N4) | 8/7/08 | 64.89787 | -148.76671 |
| CY079972 | A/mallard/Interior Alaska/8BM1966R1/2008 (H8N4) | 8/8/08 | 64.54522 | -148.51852 |
| CY080208 | A/northern pintail/Interior Alaska/8MP0262R2/2008 (H7N3) | 8/8/08 | 64.903 | -148.745 |
| CY079615 | A/northern pintail/Interior Alaska/8BM1995R1/2008 (H12N5) | 8/9/08 | 64.56281 | -148.48598 |
| CY079980 | A/northern pintail/Interior Alaska/8BM2011R1/2008 (H8N4) | 8/9/08 | 64.56281 | -148.48598 |
| CY080108 | A/northern pintail/Interior Alaska/8BM2082/2008 (H8N4) | 8/9/08 | 64.5741 | -148.45982 |
| CY080116 | A/mallard/Interior Alaska/8BM2102/2008 (H1N1) | 8/9/08 | 64.5741 | -148.45982 |
| CY079381 | A/northern pintail/Interior Alaska/8BM2046R1/2008 (H8N4) | 8/9/08 | 64.57618 | -148.45913 |
| CY079964 | A/mallard/Interior Alaska/8MP0457R1/2008 (H8N4) | 8/10/08 | 64.98129 | -149.6911 |
| CY080162 | A/mallard/Interior Alaska/8BM2312/2008 (H3N8) | 8/11/08 | 64.56281 | -148.48598 |
| CY079373 | A/mallard/Interior Alaska/8MP0547/2008 (H8N4) | 8/11/08 | 64.98129 | -149.6911 |
| CY079060 | A/northern pintail/Interior Alaska/8BM2528R1/2008 (H3N8) | 8/13/08 | 64.56281 | -148.48598 |
| CY079052 | A/northern pintail/Interior Alaska/8MP0689/2008 (H8N4) | 8/13/08 | 64.873 | -148.842 |
| CY079623 | A/northern pintail/Interior Alaska/8BM2621R1/2008 (H8N4) | 8/14/08 | 64.56281 | -148.48598 |
| CY079940 | A/mallard/Interior Alaska/8BM2714/2008 (H8N4) | 8/14/08 | 64.57618 | -148.45913 |
| CY079068 | A/mallard/Interior Alaska/8BM2967/2008 (H12N5) | 8/16/08 | 64.9603 | -148.76522 |
| CY079076 | A/northern pintail/Interior Alaska/8BM2974/2008 (H12N5) | 8/17/08 | 64.9603 | -148.76522 |
| CY079092 | A/northern pintail/Interior Alaska/8BM3041/2008 (H8N4) | 8/17/08 | 64.9603 | -148.76522 |
| CY079084 | A/northern pintail/Interior Alaska/8BM2987/2008 (H8N4) | 8/17/08 | 64.9603 | -148.76522 |
| CY079100 | A/mallard/Interior Alaska/8BM3061/2008 (H8N4) | 8/18/08 | 64.93198 | -148.80967 |
| CY079108 | A/northern pintail/Interior Alaska/8BM3088/2008 (H8N4) | 8/18/08 | 64.9603 | -148.76522 |
| CY079631 | A/northern pintail/Interior Alaska/8BM3091/2008 (H8N4) | 8/18/08 | 64.9603 | -148.76522 |
| CY079639 | A/northern pintail/Interior Alaska/8BM3137/2008 (H8N4) | 8/18/08 | 64.9603 | -148.76522 |
| CY080004 | A/mallard/Interior Alaska/8BM3092/2008 (H12N5) | 8/18/08 | 64.9603 | -148.76522 |
| CY079188 | A/mallard/Interior Alaska/8MP0792R1/2008 (H12N5) | 8/19/08 | 64.98129 | -149.6911 |
| CY080026 | A/mallard/Interior Alaska/8BM3327/2008 (H8N4) | 8/20/08 | 64.9603 | -148.76522 |
| CY079647 | A/northern shoveler/Interior Alaska/8BM3470/2008 (H9N2) | 9/1/08 | 64.98129 | -149.6911 |
| CY080048 | A/mallard/Interior Alaska/8BM3519/2008 (H12N5) | 9/7/08 | 64.98129 | -149.6911 |
| CY079116 | A/northern pintail/Interior Alaska/8BM3582/2008 (H3N8) | 9/14/08 | 64.89891 | -148.83503 |
| CY079124 | A/mallard/Interior Alaska/8BM3586/2008 (H3N8) | 9/14/08 | 64.98129 | -149.6911 |
| CY079678 | A/mallard/Interior Alaska/8BM3584R1/2008 (H8N4) | 9/14/08 | 64.98129 | -149.6911 |
| CY079662 | A/northern pintail/Interior Alaska/8BM3608/2008 (H4N6) | 9/15/08 | 64.98129 | -149.6911 |
| CY079132 | A/mallard/Interior Alaska/8BM3614/2008 (H3N8) | 9/16/08 | 64.98129 | -149.6911 |
| CY079196 | A/mallard/Interior Alaska/8BM3627R1/2008 (H4N6) | 9/16/08 | 64.98129 | -149.6911 |
| CY079140 | A/northern pintail/Interior Alaska/8BM3658/2008 (H4N6) | 9/24/08 | 64.97226 | -149.53921 |
| CY080078 | A/northern pintail/Interior Alaska/8BM3669/2008 (H4N6) | 9/25/08 | 64.97226 | -149.53921 |
| CY079148 | A/northern shoveler/Interior Alaska/8BM3684/2008 (mixed) | 9/26/08 | 64.97226 | -149.53921 |
| CY079156 | A/northern pintail/Interior Alaska/8BM3700/2008 (H3N8) | 9/26/08 | 64.97638 | -149.72532 |
| CY079164 | A/green-winged teal/Interior Alaska/8BM3706/2008 (mixed) | 9/26/08 | 64.97638 | -149.72532 |
| CY080093 | A/northern pintail/Interior Alaska/8BM3696/2008 (H3N8) | 9/26/08 | 64.97638 | -149.72532 |
| CY079172 | A/northern pintail/Interior Alaska/8BM3731/2008 (H3N8) | 9/27/08 | 64.97226 | -149.53921 |
| CY079180 | A/northern pintail/Interior Alaska/8BM3736/2008 (H12N5) | 9/27/08 | 64.97226 | -149.53921 |
| CY079670 | A/northern pintail/Interior Alaska/8BM3723/2008 (H4N1) | 9/27/08 | 64.97226 | -149.53921 |
| CY080184 | A/northern pintail/Interior Alaska/8BM3739R1/2008 (H8N4) | 9/27/08 | 64.97226 | -149.53921 |
| CY039742 | A/mallard/California/7766/2008 (H4N6) | 10/8/08 |  |  |
| CY094150 | A/mallard/California/8028/2008 (H11N2) | 10/18/08 |  |  |
| CY094158 | A/mallard/California/8035/2008 (H5N2) | 10/18/08 |  |  |
| CY094166 | A/mallard/California/8212/2008 (H6N1) | 10/18/08 |  |  |
| CY094174 | A/mallard/California/8293/2008 (H6N1) | 10/18/08 |  |  |
| CY094470 | A/gadwall/California/8203/2008 (mixed) | 10/18/08 |  |  |
| CY093608 | A/mallard/California/8118/2008 (H11N9) | 10/19/08 |  |  |
| CY093616 | A/mallard/California/8322/2008 (H6N1) | 10/19/08 |  |  |
| CY093727 | A/northern shoveler/California/8355/2008 (H6N1) | 10/19/08 |  |  |
| CY093807 | A/American wigeon/California/8121/2008 (H6N1) | 10/19/08 |  |  |
| CY093919 | A/gadwall/California/8340/2008 (H6N1) | 10/19/08 |  |  |
| CY093998 | A/northern pintail/California/8105/2008 (H6N1) | 10/19/08 |  |  |
| CY094182 | A/mallard/California/8834/2008 (H5N9) | 10/19/08 |  |  |
| CY094382 | A/American wigeon/California/8352/2008 (H12N5) | 10/19/08 |  |  |
| CY094390 | A/American wigeon/California/8363/2008 (H6N1) | 10/19/08 |  |  |
| CY094446 | A/green-winged teal/California/8326/2008 (H1N2) | 10/19/08 |  |  |
| CY079325 | A/mallard/Wisconsin/08OS2261/2008 (H4N6) | 10/19/08 | 43.550539 | -88.670654 |
| CY079333 | A/mallard/Wisconsin/08OS2271/2008 (H11N9) | 10/19/08 | 43.550539 | -88.670654 |
| CY079421 | A/American green-winged teal/Wisconsin/08OS2270/2008 (H3N8) | 10/19/08 | 43.550539 | -88.670654 |
| CY079567 | A/mallard/Wisconsin/08OS2254/2008 (mixed) | 10/19/08 | 43.550539 | -88.670654 |
| CY079429 | A/American green-winged teal/Wisconsin/08OS2291/2008 (H3N2) | 10/20/08 | 43.550539 | -88.670654 |
| CY079437 | A/gadwall/Wisconsin/08OS2296/2008 (H6N2) | 10/20/08 | 43.550539 | -88.670654 |
| CY079341 | A/American green-winged teal/Wisconsin/08OS2292/2008 (H3N2) | 10/20/08 | 43.550539 | -88.670654 |
| CY079349 | A/gadwall/Wisconsin/08OS2293/2008 (H3N2) | 10/20/08 | 43.550539 | -88.670654 |
| CY093640 | A/mallard/California/8462/2008 (H6N1) | 10/22/08 | 39.40443986 | -122.1736667 |
| CY093624 | A/mallard/California/8399/2008 (H6N1) | 10/22/08 |  |  |
| CY093632 | A/mallard/California/8457/2008 (H6N2) | 10/22/08 |  |  |
| CY093815 | A/American wigeon/California/8141/2008 (H6N1) | 10/22/08 |  |  |
| CY094190 | A/mallard/California/8416/2008 (H6N1) | 10/22/08 |  |  |
| CY094198 | A/mallard/California/8427/2008 (H6N1) | 10/22/08 |  |  |
| CY094206 | A/mallard/California/8429/2008 (H6N1) | 10/22/08 |  |  |
| CY094214 | A/mallard/California/8432/2008 (H6N1) | 10/22/08 |  |  |
| CY094518 | A/northern pintail/California/8152/2008 (H6N1) | 10/22/08 |  |  |
| CY094526 | A/northern pintail/California/8470/2008 (H6N1) | 10/22/08 |  |  |
| CY093648 | A/mallard/California/8519/2008 (H6N1) | 10/25/08 |  |  |
| CY093823 | A/American wigeon/California/8529/2008 (H6N1) | 10/25/08 |  |  |
| CY093831 | A/American wigeon/California/8547/2008 (H6N1) | 10/25/08 |  |  |
| CY093863 | A/bufflehead/California/8522/2008 (H4N8) | 10/25/08 |  |  |
| CY093879 | A/green-winged teal/California/7972/2008 (H6N2) | 10/25/08 |  |  |
| CY093927 | A/gadwall/California/8504/2008 (H6N1) | 10/25/08 |  |  |
| CY094222 | A/mallard/California/8518/2008 (H6N1) | 10/25/08 |  |  |
| CY094270 | A/northern shoveler/California/8528/2008 (H6N1) | 10/25/08 |  |  |
| CY094278 | A/northern shoveler/California/8536/2008 (H6N1) | 10/25/08 |  |  |
| CY094478 | A/gadwall/California/8535/2008 (H6N1) | 10/25/08 |  |  |
| CY079445 | A/mallard/Illinois/08OS2315/2008 (H4N6) | 10/25/08 | 40.75662 | -89.533081 |
| CY079575 | A/American green-winged teal/Illinois/08OS2311/2008 (mixed) | 10/25/08 |  |  |
| CY079469 | A/American green-winged teal/Illinois/08OS2713/2008 (H10N7) | 10/26/08 | 40.75662 | -89.533081 |
| CY079357 | A/mallard/Illinois/08OS2710/2008 (H10N7) | 10/26/08 |  |  |
| CY079453 | A/American black duck/Illinois/08OS2688/2008 (H5N2) | 10/26/08 |  |  |
| CY079461 | A/mallard/Illinois/08OS2711/2008 (H10N7) | 10/26/08 |  |  |
| CY093735 | A/northern shoveler/California/8673/2008 (H6N1) | 10/29/08 |  |  |
| CY093839 | A/American wigeon/California/8670/2008 (H6N1) | 10/29/08 |  |  |
| CY094286 | A/northern shoveler/California/8629/2008 (H6N1) | 10/29/08 |  |  |
| CY094294 | A/northern shoveler/California/8675/2008 (H11N9) | 10/29/08 |  |  |
| CY094398 | A/American wigeon/California/8658/2008 (H6N1) | 10/29/08 |  |  |
| CY094454 | A/green-winged teal/California/8612/2008 (H6N1) | 10/29/08 |  |  |
| CY094494 | A/greater white-fronted goose/California/6365/2008 (H6N1) | 10/29/08 |  |  |
| CY079477 | A/mallard/Wisconsin/08OS2841/2008 (H2N3) | 10/31/08 | 43.550539 | -88.670654 |
| CY079493 | A/mallard/Wisconsin/08OS2844/2008 (H2N3) | 10/31/08 | 43.550539 | -88.670654 |
| CY093847 | A/American wigeon/California/8763/2008 (H6N1) | 11/1/08 |  |  |
| CY094086 | A/mallard/California/6404/2008 (H6N1) | 11/1/08 |  |  |
| CY094094 | A/mallard/California/6420/2008 (H6N2) | 11/1/08 |  |  |
| CY094486 | A/gadwall/California/8708/2008 (H6N1) | 11/1/08 |  |  |
| CY094534 | A/northern pintail/California/8764/2008 (H6N1) | 11/1/08 |  |  |
| CY093592 | A/mallard/California/6469/2008 (H6N2) | 11/2/08 |  |  |
| CY093600 | A/mallard/California/6471/2008 (H3N8) | 11/2/08 |  |  |
| CY093958 | A/greater white-fronted goose/California/6461/2008 (H6N1) | 11/2/08 |  |  |
| CY093982 | A/northern pintail/California/6495/2008 (H6N1) | 11/2/08 |  |  |
| CY094102 | A/mallard/California/6490/2008 (H12N5) | 11/2/08 |  |  |
| CY093966 | A/greater white-fronted goose/California/6548/2008 (H6N1) | 11/5/08 | 39.40443986 | -122.1736667 |
| CY093656 | A/mallard/California/8843/2008 (H1N1) | 11/5/08 |  |  |
| CY094110 | A/mallard/California/6517/2008 (H11N9) | 11/5/08 |  |  |
| CY094118 | A/mallard/California/6524/2008 (H12N5) | 11/5/08 |  |  |
| CY093791 | A/American wigeon/California/6588/2008 (H6N1) | 11/8/08 |  |  |
| CY093799 | A/American wigeon/California/6610/2008 (H12N5) | 11/8/08 |  |  |
| CY094406 | A/American wigeon/California/8910/2008 (H6N1) | 11/8/08 |  |  |
| CY094126 | A/mallard/California/6634/2008 (H11N9) | 11/9/08 |  |  |
| CY094302 | A/northern shoveler/California/9017/2008 (H11N2) | 11/12/08 |  |  |
| CY093990 | A/northern pintail/California/6763/2008 (H6N1) | 11/15/08 |  |  |
| CY094134 | A/mallard/California/6772/2008 (H4N6) | 11/15/08 |  |  |
| CY094414 | A/American wigeon/California/9044/2008 (H6N1) | 11/15/08 |  |  |
| CY079509 | A/northern shoveler/Illinois/08OS3331/2008 (H4N8) | 11/15/08 | 40.75662 | -89.533081 |
| CY094510 | A/northern pintail/California/6791/2008 (H11N2) | 11/16/08 |  |  |
| CY093759 | A/northern shoveler/California/9140/2008 (H1N9) | 11/19/08 |  |  |
| CY093942 | A/gadwall/California/9155/2008 (H6N1) | 11/19/08 |  |  |
| CY093767 | A/northern shoveler/California/9235/2008 (H10N8) | 11/22/08 |  |  |
| CY094142 | A/mallard/California/6957/2008 (H10N7) | 11/22/08 |  |  |
| CY094310 | A/northern shoveler/California/9187/2008 (H6N2) | 11/22/08 |  |  |
| CY094318 | A/northern shoveler/California/9228/2008 (H4N6) | 11/22/08 |  |  |
| CY093871 | A/green-winged teal/California/6990/2008 (H6N1) | 11/23/08 |  |  |
| CY094326 | A/northern shoveler/California/9267/2008 (H4N6) | 11/23/08 |  |  |
| CY093974 | A/greater white-fronted goose/California/10936/2008 (H1N1) | 11/29/08 |  |  |
| CY094230 | A/mallard/California/9573/2008 (H4N6) | 11/30/08 |  |  |
| CY093688 | A/mallard/California/10985/2008 (H3N8) | 12/3/08 |  |  |
| CY093703 | A/mallard/California/11100/2008 (H11N2) | 12/6/08 |  |  |
| CY093711 | A/mallard/California/11119/2008 (H11N9) | 12/6/08 |  |  |
| CY094246 | A/mallard/California/11095/2008 (H4N6) | 12/6/08 |  |  |
| CY093664 | A/mallard/California/9704/2008 (H10N7) | 12/7/08 |  |  |
| CY093775 | A/northern shoveler/California/9680/2008 (H6N2) | 12/7/08 |  |  |
| CY093783 | A/northern shoveler/California/9710/2008 (H10N7) | 12/7/08 |  |  |
| CY093672 | A/mallard/California/10125/2008 (H11N9) | 12/13/08 | 39.40443986 | -122.1736667 |
| CY094462 | A/green-winged teal/California/11275/2008 (H7N3) | 12/13/08 | 39.40443986 | -122.1736667 |
| CY093680 | A/mallard/California/10126/2008 (H10N7) | 12/13/08 |  |  |
| CY093903 | A/green-winged teal/California/11285/2008 (mixed) | 12/13/08 |  |  |
| CY094254 | A/mallard/California/11353/2008 (H10N2) | 12/13/08 |  |  |
| CY093887 | A/green-winged teal/California/10197/2008 (H10N7) | 12/14/08 |  |  |
| CY094334 | A/northern shoveler/California/9781/2008 (H1N3) | 12/14/08 |  |  |
| CY094238 | A/mallard/California/10064/2008 (H1N6) | 12/21/08 |  |  |
| CY094342 | A/northern shoveler/California/10024/2008 (H4N4) | 12/21/08 |  |  |
| CY093911 | A/green-winged teal/California/00083/2009 (H10N7) | 1/3/09 |  |  |
| CY079389 | A/northern shoveler/Mississippi/09OS025/2009 (H12N5) | 1/9/09 | 32.529447 | -89.954681 |
| CY079309 | A/American green-winged teal/Mississippi/09OS046/2009 (H7N7) | 1/9/09 | 32.529447 | -89.954681 |
| CY079397 | A/northern shoveler/Mississippi/09OS168/2009 (H10N6) | 1/11/09 | 32.529447 | -89.954681 |
| CY079413 | A/northern shoveler/Mississippi/09OS643/2009 (H7N7) | 1/15/09 | 32.529447 | -89.954681 |
| CY094574 | A/green-winged teal/California/1841/2009 (H7N3) | 1/18/09 | 39.40443986 | -122.1736667 |
| CY079405 | A/American coot/Mississippi/09OS615/2009 (H10N3) | 1/24/09 | 32.529447 | -89.954681 |
| CY080147 | A/mallard/Interior Alaska/9BM1358/2009 (H8N4) | 6/16/09 | 64.98129 | -149.6911 |
| CY078868 | A/mallard/Interior Alaska/9BM1585R1/2009 (H12N5) | 6/24/09 | 64.91269 | -148.77545 |
| CY079779 | A/mallard/Interior Alaska/9BM1799/2009 (H12N5) | 7/1/09 | 64.91269 | -148.77545 |
| CY078748 | A/mallard/Interior Alaska/9BM1869/2009 (H4N6) | 7/1/09 | 64.98129 | -149.6911 |
| CY079583 | A/mallard/Interior Alaska/9BM1864/2009 (H4N6) | 7/1/09 | 64.98129 | -149.6911 |
| CY079801 | A/mallard/Interior Alaska/9BM1870/2009 (H4N6) | 7/1/09 | 64.98129 | -149.6911 |
| CY078716 | A/mallard/Interior Alaska/9BM1812/2009 (H4N6) | 7/2/09 | 64.98129 | -149.6911 |
| CY078732 | A/mallard/Interior Alaska/9BM1856/2009 (H4N6) | 7/2/09 | 64.98129 | -149.6911 |
| CY078740 | A/mallard/Interior Alaska/9BM1807/2009 (H4N6) | 7/2/09 | 64.98129 | -149.6911 |
| CY079793 | A/mallard/Interior Alaska/9BM1809/2009 (H4N6) | 7/2/09 | 64.98129 | -149.6911 |
| CY078780 | A/mallard/Interior Alaska/9BM1962/2009 (H4N6) | 7/4/09 | 64.873 | -148.842 |
| CY078756 | A/mallard/Interior Alaska/9BM1947/2009 (H4N6) | 7/4/09 | 64.98129 | -149.6911 |
| CY078764 | A/mallard/Interior Alaska/9BM1949/2009 (H4N6) | 7/4/09 | 64.98129 | -149.6911 |
| CY078772 | A/mallard/Interior Alaska/9BM1958/2009 (H4N6) | 7/4/09 | 64.98129 | -149.6911 |
| CY079591 | A/mallard/Interior Alaska/9BM1957/2009 (H4N6) | 7/4/09 | 64.98129 | -149.6911 |
| CY079816 | A/mallard/Interior Alaska/9BM1959/2009 (H4N6) | 7/4/09 | 64.98129 | -149.6911 |
| CY078724 | A/mallard/Interior Alaska/9BM1852/2009 (H10N5) | 7/6/09 | 64.9143 | -148.75865 |
| CY078796 | A/mallard/Interior Alaska/9BM1967/2009 (H4N6) | 7/6/09 | 64.873 | -148.842 |
| CY078788 | A/mallard/Interior Alaska/9BM1965/2009 (H4N6) | 7/6/09 | 64.98129 | -149.6911 |
| CY078804 | A/mallard/Interior Alaska/9BM1974/2009 (H4N6) | 7/6/09 | 64.98129 | -149.6911 |
| CY078812 | A/mallard/Interior Alaska/9BM1975/2009 (H4N6) | 7/6/09 | 64.98129 | -149.6911 |
| CY079599 | A/mallard/Interior Alaska/9BM1964/2009 (H4N6) | 7/6/09 | 64.98129 | -149.6911 |
| CY079824 | A/mallard/Interior Alaska/9BM1968/2009 (H4N6) | 7/6/09 | 64.98129 | -149.6911 |
| CY079832 | A/mallard/Interior Alaska/9BM1969/2009 (H4N6) | 7/6/09 | 64.98129 | -149.6911 |
| CY078820 | A/mallard/Interior Alaska/9BM2057/2009 (H4N6) | 7/8/09 | 64.98129 | -149.6911 |
| CY078828 | A/mallard/Interior Alaska/9BM2077/2009 (H4N6) | 7/9/09 | 64.98129 | -149.6911 |
| CY079848 | A/mallard/Interior Alaska/9BM2073/2009 (H4N6) | 7/9/09 | 64.98129 | -149.6911 |
| CY078836 | A/mallard/Interior Alaska/9BM2168/2009 (H4N6) | 7/10/09 | 64.98129 | -149.6911 |
| CY078844 | A/mallard/Interior Alaska/9BM2170/2009 (H4N6) | 7/10/09 | 64.98129 | -149.6911 |
| CY079863 | A/mallard/Interior Alaska/9BM2164/2009 (H4N6) | 7/10/09 | 64.98129 | -149.6911 |
| CY078852 | A/mallard/Interior Alaska/9BM2254/2009 (H4N6) | 7/11/09 | 64.98129 | -149.6911 |
| CY078860 | A/mallard/Interior Alaska/9BM2256/2009 (H4N6) | 7/11/09 | 64.98129 | -149.6911 |
| CY079607 | A/mallard/Interior Alaska/9BM2259/2009 (H4N6) | 7/11/09 | 64.98129 | -149.6911 |
| CY079871 | A/mallard/Interior Alaska/9BM2239/2009 (H4N6) | 7/11/09 | 64.98129 | -149.6911 |
| CY079879 | A/mallard/Interior Alaska/9BM2243/2009 (H4N6) | 7/11/09 | 64.98129 | -149.6911 |
| CY079887 | A/mallard/Interior Alaska/9BM2252/2009 (H4N6) | 7/11/09 | 64.98129 | -149.6911 |
| CY094638 | A/mallard/California/5495/2009 (H4N2) | 7/23/09 |  |  |
| CY094646 | A/mallard/California/5502/2009 (H5N2) | 7/23/09 |  |  |
| CY094694 | A/mallard/California/5319/2009 (H5N2) | 7/23/09 |  |  |
| CY094726 | A/mallard/California/5491/2009 (H5N2) | 7/23/09 |  |  |
| CY094654 | A/mallard/California/5149/2009 (H5N2) | 7/25/09 |  |  |
| CY094662 | A/mallard/California/5174/2009 (H5N2) | 7/25/09 |  |  |
| CY094014 | A/mallard/California/5219/2009 (H5N2) | 7/27/09 |  |  |
| CY094582 | A/mallard/California/5212/2009 (H5N2) | 7/27/09 |  |  |
| CY094590 | A/mallard/California/5222/2009 (H5N2) | 7/27/09 |  |  |
| CY094670 | A/mallard/California/5192/2009 (H4N2) | 7/27/09 |  |  |
| CY094678 | A/mallard/California/5205/2009 (mixed) | 7/27/09 |  |  |
| CY094734 | A/mallard/California/5191/2009 (H5N2) | 7/27/09 |  |  |
| CY094686 | A/mallard/California/5255/2009 (H3N8) | 7/28/09 | 39.40443986 | -122.1736667 |
| CY094598 | A/mallard/California/5250/2009 (H5N2) | 7/28/09 |  |  |
| CY094606 | A/mallard/California/5271/2009 (H4N6) | 7/29/09 |  |  |
| CY094614 | A/mallard/California/5276/2009 (H5N2) | 7/29/09 |  |  |
| CY094622 | A/mallard/California/5282/2009 (mixed) | 7/29/09 |  |  |
| CY094630 | A/mallard/California/5296/2009 (H5N2) | 7/29/09 |  |  |
| CY097126 | A/mallard/Ohio/2039/2009 (mixed) | 8/3/09 | 40.161034 | -82.888412 |
| CY097136 | A/mallard/Ohio/2043/2009 (H6N1) | 8/3/09 | 40.161034 | -82.888412 |
| CY097655 | A/mallard/Ohio/2031/2009 (H4N9) | 8/3/09 | 40.161034 | -82.888412 |
| CY097118 | A/mallard/Ohio/2033/2009 (H4N9) | 8/3/09 | 40.161034 | -82.888412 |
| CY094702 | A/mallard/California/5351/2009 (H1N1) | 8/4/09 |  |  |
| CY094710 | A/mallard/California/5359/2009 (H5N2) | 8/4/09 |  |  |
| CY094718 | A/mallard/California/5386/2009 (H5N2) | 8/4/09 |  |  |
| CY097102 | A/mallard/Ohio/1686/2009 (H6N9) | 8/10/09 | 40.161034 | -82.888412 |
| CY097647 | A/mallard/Ohio/1688/2009 (H12N5) | 8/10/09 | 40.161034 | -82.888412 |
| CY097110 | A/mallard/Ohio/1690/2009 (H6N1) | 8/10/09 | 40.161034 | -82.888412 |
| CY070885 | A/glaucous-winged gull/Southcentral Alaska/9JR0781R0/2009 (H13N6) | 8/18/09 | 60.55 | -145.77 |
| CY070869 | A/glaucous-winged gull/Southcentral Alaska/9JR0747R0/2009 (H13N6) | 8/18/09 | 60.55 | -145.77 |
| CY097278 | A/mallard/Wisconsin/1534/2009 (H6N8) | 8/18/09 | 43.550539 | -88.670654 |
| CY097286 | A/mallard/Wisconsin/1538/2009 (H4N6) | 8/18/09 | 43.550539 | -88.670654 |
| CY097663 | A/mallard/Ohio/1695/2009 (H4N6) | 8/25/09 | 40.161034 | -82.888412 |
| CY097431 | A/northern shoveler/Wisconsin/2508/2009 (H4N2) | 10/3/09 | 43.550539 | -88.670654 |
| CY097439 | A/blue-winged teal/Wisconsin/2509/2009 (H1N2) | 10/3/09 | 43.550539 | -88.670654 |
| CY097511 | A/blue-winged teal/Wisconsin/2818/2009 (mixed) | 10/3/09 | 43.550539 | -88.670654 |
| CY097447 | A/mallard/Wisconsin/2530/2009 (H4N2) | 10/4/09 | 43.550539 | -88.670654 |
| CY096979 | A/American black duck/Wisconsin/2542/2009 (H4N2) | 10/5/09 | 43.550539 | -88.670654 |
| CY096987 | A/mallard/Wisconsin/2543/2009 (H3N2) | 10/5/09 | 43.550539 | -88.670654 |
| CY097327 | A/mallard/Wisconsin/2549/2009 (H3N2) | 10/5/09 | 43.550539 | -88.670654 |
| CY097335 | A/mallard/Wisconsin/2560/2009 (H2N3) | 10/5/09 | 43.550539 | -88.670654 |
| CY096995 | A/blue-winged teal/Wisconsin/2572/2009 (H2N3) | 10/6/09 | 43.550539 | -88.670654 |
| CY097343 | A/mallard/Wisconsin/2575/2009 (H3N2) | 10/6/09 | 43.550539 | -88.670654 |
| CY097351 | A/mallard/Wisconsin/2576/2009 (H5N1) | 10/6/09 | 43.550539 | -88.670654 |
| CY097035 | A/mallard/Wisconsin/2712/2009 (H3N6) | 10/11/09 | 43.550539 | -88.670654 |
| CY097196 | A/American green-winged teal/Illinois/3054/2009 (H1N2) | 10/17/09 | 40.75662 | -89.533081 |
| CY096925 | A/American green-winged teal/Illinois/2975/2009 (mixed) | 10/17/09 |  |  |
| CY096935 | A/mallard/Illinois/3048/2009 (H11N2) | 10/17/09 |  |  |
| CY096943 | A/mallard/Illinois/3051/2009 (H11N3) | 10/17/09 |  |  |
| CY097168 | A/mallard/Illinois/2956/2009 (mixed) | 10/17/09 |  |  |
| CY097177 | A/mallard/Illinois/3974/2009 (H5N2) | 10/17/09 |  |  |
| CY097186 | A/American green-winged teal/Illinois/3053/2009 (mixed) | 10/17/09 |  |  |
| CY097204 | A/American green-winged teal/Illinois/2479/2009 (H2N3) | 10/17/09 |  |  |
| CY097011 | A/mallard/Wisconsin/2653/2009 (H4N6) | 10/17/09 | 43.550539 | -88.670654 |
| CY097019 | A/blue-winged teal/Wisconsin/2713/2009 (H4N2) | 10/17/09 | 43.550539 | -88.670654 |
| CY097027 | A/blue-winged teal/Wisconsin/2741/2009 (H4N6) | 10/17/09 | 43.550539 | -88.670654 |
| CY097212 | A/blue-winged teal/Wisconsin/3060/2009 (H3N2) | 10/17/09 | 43.550539 | -88.670654 |
| CY097220 | A/blue-winged teal/Wisconsin/3061/2009 (mixed) | 10/17/09 | 43.550539 | -88.670654 |
| CY097463 | A/blue-winged teal/Wisconsin/2649/2009 (H6N1) | 10/17/09 | 43.550539 | -88.670654 |
| CY097471 | A/blue-winged teal/Wisconsin/2665/2009 (H4N2) | 10/17/09 | 43.550539 | -88.670654 |
| CY097479 | A/mallard/Wisconsin/2719/2009 (H4N2) | 10/17/09 | 43.550539 | -88.670654 |
| CY097487 | A/blue-winged teal/Wisconsin/2720/2009 (H3N2) | 10/17/09 | 43.550539 | -88.670654 |
| CY097495 | A/northern pintail/Wisconsin/2737/2009 (H3N2) | 10/17/09 | 43.550539 | -88.670654 |
| CY097503 | A/American green-winged teal/Wisconsin/2743/2009 (H1N1) | 10/17/09 | 43.550539 | -88.670654 |
| CY097455 | A/American green-winged teal/Wisconsin/2530/2009 (H6N2) | 10/17/09 | 43.550539 | -88.670654 |
| CY097359 | A/American green-winged teal/Wisconsin/2690/2009 (H3N8) | 10/20/09 | 43.550539 | -88.670654 |
| CY097043 | A/blue-winged teal/Wisconsin/2753/2009 (H4N6) | 10/24/09 | 43.550539 | -88.670654 |
| CY094542 | A/mallard/California/6744/2009 (H6N1) | 10/25/09 |  |  |
| CY094550 | A/mallard/California/6768/2009 (H1N1) | 10/25/09 |  |  |
| CY094558 | A/American wigeon/California/6712/2009 (mixed) | 10/25/09 |  |  |
| CY094566 | A/mallard/California/6695/2009 (H6N1) | 10/25/09 |  |  |
| CY097003 | A/mallard/Wisconsin/2755/2009 (H1N1) | 10/30/09 | 43.550539 | -88.670654 |
| CY097367 | A/mallard/Wisconsin/2756/2009 (H1N1) | 10/30/09 | 43.550539 | -88.670654 |
| CY096962 | A/American green-winged teal/Illinois/3443/2009 (mixed) | 10/31/09 |  |  |
| CY097160 | A/American coot/Illinois/3405/2009 (H10N3) | 10/31/09 |  |  |
| CY097375 | A/mallard/Wisconsin/2785/2009 (H2N3) | 10/31/09 | 43.550539 | -88.670654 |
| CY097383 | A/mallard/Wisconsin/3165/2009 (H1N1) | 10/31/09 | 43.550539 | -88.670654 |
| CY097059 | A/ruddy duck/Illinois/3471/2009 (H3N8) | 11/1/09 |  |  |
| CY097076 | A/mallard/Iowa/3195/2009 (mixed) | 11/7/09 | 41.479776 | -93.218994 |
| CY097583 | A/mallard/Iowa/3193/2009 (H11N9) | 11/7/09 | 41.479776 | -93.218994 |
| CY097067 | A/common goldeneye/Iowa/3192/2009 (H11N9) | 11/7/09 | 41.479776 | -93.218994 |
| CY097085 | A/mallard/Iowa/3205/2009 (mixed) | 11/8/09 | 41.479776 | -93.218994 |
| CY096951 | A/northern shoveler/Illinois/3767/2009 (mixed) | 11/14/09 |  |  |
| CY097230 | A/mallard/Illinois/3747/2009 (H6N1) | 11/14/09 |  |  |
| CY097051 | A/American black duck/Illinois/3854/2009 (H11N9) | 11/15/09 |  |  |
| CY097519 | A/gadwall/Illinois/3860/2009 (H6N1) | 11/15/09 |  |  |
| CY097294 | A/lesser scaup/Wisconsin/3964/2009 (H10N3) | 11/23/09 | 43.550539 | -88.670654 |
| CY096971 | A/bufflehead/Illinois/4016/2009 (H4N8) | 11/28/09 |  |  |
| CY097535 | A/American black duck/Illinois/4119/2009 (H8N4) | 11/29/09 | 40.75662 | -89.533081 |
| CY097527 | A/lesser scaup/Illinois/4115/2009 (H10N7) | 11/29/09 |  |  |
| CY097543 | A/msllard/Illinois/4124/2009 (H4N8) | 11/29/09 |  |  |
| CY097551 | A/ring-necked duck/Illinois/4125/2009 (H4N8) | 11/29/09 |  |  |
| CY097559 | A/mallard/Illinois/4162/2009 (H11N2) | 11/30/09 |  |  |
| CY097567 | A/mallard/Illinois/4179/2009 (H11N9) | 11/30/09 |  |  |
| CY097575 | A/mallard/Illinois/4180/2009 (H6N1) | 11/30/09 |  |  |
| CY097391 | A/mallard/Wisconsin/4194/2009 (H1N1) | 12/3/09 | 43.550539 | -88.670654 |
| CY097399 | A/mallard/Wisconsin/4196/2009 (mixed) | 12/3/09 | 43.550539 | -88.670654 |
| CY097407 | A/mallard/Wisconsin/4197/2009 (H1N1) | 12/3/09 | 43.550539 | -88.670654 |
| CY097415 | A/northern pintail/Wisconsin/4198/2009 (H11N9) | 12/3/09 | 43.550539 | -88.670654 |
| CY097423 | A/mallard/Wisconsin/4203/2009 (H11N9) | 12/3/09 | 43.550539 | -88.670654 |
| CY097270 | A/mallard/Wisconsin/4218/2009 (H12N5) | 12/5/09 | 43.550539 | -88.670654 |
| CY097094 | A/mallard/Missouri/350/2009 (H11N9) | 12/6/09 | 38.212288 | -92.790527 |
| CY097631 | A/northern shoveler/Missouri/298/2009 (H9N2) | 12/6/09 | 38.212288 | -92.790527 |
| CY097639 | A/northern pintail/Missouri/319/2009 (H12N5) | 12/6/09 | 38.212288 | -92.790527 |
| CY097302 | A/mallard/Wisconsin/4230/2009 (H10N1) | 12/6/09 | 43.550539 | -88.670654 |
| CY097310 | A/greater scaup/Wisconsin/4234/2009 (H11N9) | 12/6/09 | 43.550539 | -88.670654 |
| CY097318 | A/mallard/Wisconsin/4236/2009 (mixed) | 12/6/09 | 43.550539 | -88.670654 |
| CY097591 | A/mallard/Missouri/129/2009 (H6N2) | 12/7/09 | 38.212288 | -92.790527 |
| CY097615 | A/northern shoveler/Missouri/196/2009 (H10N3) | 12/7/09 | 38.212288 | -92.790527 |
| CY097623 | A/mallard/Missouri/220/2009 (H7N3) | 12/7/09 | 38.212288 | -92.790527 |
| CY097599 | A/mallard/Missouri/132/2009 (H2N3) | 12/7/09 | 38.212288 | -92.790527 |
| CY097671 | A/northern shoveler/Mississippi/252/2010 (H10N7) | 1/10/10 | 32.529447 | -89.954681 |
| CY097144 | A/northern shoveler/Mississippi/236/2010 (H10N7) | 1/12/10 | 32.529447 | -89.954681 |
| CY097679 | A/American green-winged teal/Mississippi/285/2010 (H3N8) | 1/14/10 | 32.529447 | -89.954681 |
| CY097687 | A/American green-winged teal/Mississippi/300/2010 (H11N9) | 1/16/10 | 32.529447 | -89.954681 |
| CY097695 | A/mallard/Mississippi/329/2010 (H10N7) | 1/16/10 | 32.529447 | -89.954681 |
| CY097703 | A/mallard/Mississippi/354/2010 (H3N8) | 1/16/10 | 32.529447 | -89.954681 |
| CY097711 | A/mallard/Mississippi/360/2010 (H3N8) | 1/16/10 | 32.529447 | -89.954681 |
| CY097743 | A/American green-winged teal/Mississippi/404/2010 (mixed) | 1/16/10 | 32.529447 | -89.954681 |
| CY097753 | A/mallard/Mississippi/407/2010 (mixed) | 1/16/10 | 32.529447 | -89.954681 |
| CY097719 | A/mallard/Mississippi/386/2010 (H3N8) | 1/17/10 | 32.529447 | -89.954681 |
| CY097727 | A/mallard/Mississippi/390/2010 (H3N8) | 1/17/10 | 32.529447 | -89.954681 |
| CY097735 | A/northern shoveler/Mississippi/397/2010 (H1N3) | 1/17/10 | 32.529447 | -89.954681 |
| CY097763 | A/mallard/Mississippi/413/2010 (H1N1) | 1/17/10 | 32.529447 | -89.954681 |
| CY097771 | A/mallard/Mississippi/442/2010 (H1N1) | 1/19/10 | 32.529447 | -89.954681 |
| CY094742 | A/mallard/California/1154/2010 (mixed) | 7/27/10 | 39.40443986 | -122.1736667 |
| CY094750 | A/mallard/California/1156/2010 (H4N6) | 7/27/10 | 39.40443986 | -122.1736667 |
| CY094758 | A/mallard/California/1210/2010 (H4N6) | 7/29/10 | 39.40443986 | -122.1736667 |
| CY094774 | A/mallard/California/1188/2010 (H4N6) | 7/29/10 | 39.40443986 | -122.1736667 |
| CY094782 | A/mallard/California/1289/2010 (H4N6) | 7/30/10 | 39.40443986 | -122.1736667 |
| CY094766 | A/mallard/California/1305/2010 (H10N7) | 8/3/10 | 39.40443986 | -122.1736667 |
| CY094790 | A/mallard/California/1353/2010 (H10N7) | 8/6/10 | 39.40443986 | -122.1736667 |
