# Supplementary material for: Influenza A Virus Migration and Persistence in North American Wild Birds
Source: PLoS Pathog. 2013 Aug 29;9(8):e1003570. doi: 10.1371/journal.ppat.1003570 (PMC3757048; doi:10.1371/journal.ppat.1003570)
Supplement: Table S3 — Associated geographic metadata and exact date of sampling of newly sequenced avian influenza A viruses. (DOC) [file ppat.1003570.s020.doc]

**Table S3:** Associated geographic metadata and exact date of sampling of newly sequenced avian influenza A viruses

| **Isolate Name** | **Date of isolation** | **Latitude** | **Longitude** |
| --- | --- | --- | --- |
| A/laughing gull/New Jersey/798/1986 (H2N7) | 12/5/86 | 39.11833 | -74.89105 |
| A/blue-winged teal/Alberta/427/1986 (H4N6) | 8/15/86 | 53.3501 | -113.01867 |
| A/mallard/Alberta/575/1986 (H4N6) | 8/20/86 | 53.0168 | -113.05197 |
| A/mallard/Alberta/772/1986 (H3N8) | 8/26/86 | 53.0168 | -113.05197 |
| A/mallard/Alberta/782/1986 (H3N8) | 8/26/86 | 53.0168 | -113.05197 |
| A/laughing gull/Delaware Bay/2718/1987 (H9N5) | 5/19/87 | 39.11833 | -74.89105 |
| A/ruddy turnstone/Delaware Bay/2795/1987 (H9N5) | 5/19/87 | 39.11833 | -74.89105 |
| A/ruddy turnstone/Delaware Bay/2830/1987 (H9N5) | 5/19/87 | 39.11833 | -74.89105 |
| A/laughing gull/Delaware Bay/2838/1987 (H13N2) | 5/19/87 | 39.11833 | -74.89105 |
| A/ruddy turnstone/Delaware Bay/2774/1987 (H9N5) | 5/20/87 | 39.11833 | -74.89105 |
| A/mallard/Alberta/16/1987 (H3N8) | 4/8/87 | 53.1645 | -112.6223 |
| A/pintail/Alberta/128/1987 (H3N8) | 12/8/87 | 53.0168 | -113.05197 |
| A/blue-winged teal/Alberta/130/1987 (H3N8) | 12/8/87 | 53.0168 | -113.05197 |
| A/mallard/Alberta/140/1987 (H3N8) | 8/13/87 | 53.1168 | -112.9519 |
| A/mallard/Alberta/144/1987 (mixed) | 8/13/87 | 53.1168 | -112.9519 |
| A/red knot/Delaware/546/1988 (H4N6) | 5/15/88 | 38.945 | -75.314 |
| A/ruddy turnstone/Delaware/519/1988 (H2N1) | 5/15/88 | 38.945 | -75.314 |
| A/red knot/Delaware/541/1988 (H4N6) | 5/15/88 | 38.945 | -75.314 |
| A/shorebird/Delaware Bay/718/1988 (H4N6) | 5/17/88 | 39.11833 | -74.89105 |
| A/ruddy turnstone/Virginia/2297/1988 (H9N9) | 5/28/88 | 37.92167 | -76.04 |
| A/ruddy turnstone/Delaware/2368/1988 (H4N9) | 5/31/88 | 38.945 | -75.314 |
| A/ruddy turnstone/Delaware/2373/1988 (H4N6) | 5/31/88 | 38.945 | -75.314 |
| A/ruddy turnstone/Delaware/2354/1988 (H4N6) | 5/31/88 | 38.945 | -75.314 |
| A/laughing gull/Delaware/2971/1988 (H9N9) | 8/22/88 | 38.945 | -75.314 |
| A/mallard/Alberta/323/1988 (H2N1) | 3/9/88 | 53.4507 | -112.53 |
| A/mallard/Alberta/353/1988 (H2N3) | 3/9/88 | 53.3501 | -113.01867 |
| A/mallard/Alberta/378/1988 (H3N8) | 4/9/88 | 53.4507 | -112.53 |
| A/mallard/Alberta/20/1989 (H3N8) | 2/8/89 | 53.1168 | -112.9519 |
| A/blue-winged teal/Alberta/134/1989 (H3N6) | 8/22/89 | 53.4507 | -112.53 |
| A/mallard/Alberta/195/1989 (H7N3) | 3/9/89 | 53.3501 | -113.01867 |
| A/ruddy turnstone/Delaware/24/1990 (mixed) | 9/5/90 | 38.945 | -75.314 |
| A/ruddy turnstone/New Jersey/138/1990 (H3N6) | 5/26/90 | 39.11833 | -74.89105 |
| A/herring gull/New Jersey/159/1990 (H3N6) | 5/26/90 | 39.11833 | -74.89105 |
| A/laughing gull/New Jersey/180/1990 (H3N6) | 5/26/90 | 39.11833 | -74.89105 |
| A/semi-palmated sandpiper/New Jersey/237/1990 (H3N6) | 5/27/90 | 39.1875 | -74.95056 |
| A/semi-palmated sandpiper/New Jersey/253/1990 (H3N6) | 5/27/90 | 39.1875 | -74.95056 |
| A/ruddy turnstone/New Jersey/260/1990 (H3N6) | 5/27/90 | 39.1875 | -74.95056 |
| A/blue-winged teal/Alberta/121/1990 (H4N6) | 8/27/90 | 53.1168 | -112.9519 |
| A/mallard/Alberta/220/1990 (H3N8) | 8/28/90 | 53.3501 | -113.01867 |
| A/ruddy turnstone/Delaware Bay/231/1991 (H6N8) | 5/24/91 | 39.11833 | -74.89105 |
| A/mallard/Alberta/12/1991 (H4N6) | 9/8/91 | 53.4507 | -112.53 |
| A/mallard/Alberta/18/1991 (H4N6) | 9/8/91 | 53.4507 | -112.53 |
| A/mallard/Alberta/32/1991 (H3N8) | 9/8/91 | 53.4507 | -112.53 |
| A/mallard/Alberta/60/1991 (H3N8) | 9/8/91 | 53.41684 | -112.9687 |
| A/mallard/Alberta/61/1991 (H3N8) | 9/8/91 | 53.41684 | -112.9687 |
| A/mallard/Alberta/62/1991 (H3N8) | 9/8/91 | 53.41684 | -112.9687 |
| A/mallard/Alberta/64/1991 (H3N8) | 9/8/91 | 53.41684 | -112.9687 |
| A/pintail/Alberta/81/1991 (H3N8) | 9/8/91 | 53.3501 | -113.01867 |
| A/mallard/Alberta/54/1991 (H3N8) | 9/8/91 | 52.5833 | -99.3333 |
| A/pintail/Alberta/105/1991 (H3N8) | 10/8/91 | 53.0168 | -113.05197 |
| A/mallard/Alberta/107/1991 (mixed) | 10/8/91 | 52.7334 | -110.5849 |
| A/mallard/Alberta/109/1991 (H3N8) | 10/8/91 | 52.7334 | -110.5849 |
| A/mallard/Alberta/113/1991 (mixed) | 10/8/91 | 52.7334 | -110.5849 |
| A/mallard/Alberta/115/1991 (H3N8) | 10/8/91 | 52.7334 | -110.5849 |
| A/pintail/Alberta/118/1991 (H3N8) | 10/8/91 | 52.7334 | -110.5849 |
| A/mallard/Alberta/129/1991 (H3N6) | 8/26/91 | 53.4507 | -112.53 |
| A/mallard/Alberta/35/1992 (H3N6) | 11/8/92 | 52.48843 | -112.93053 |
| A/mallard/Alberta/196/1992 (mixed) | 8/24/92 | 50.65 | -114.28 |
| A/American wigeon/Alberta/215/1992 (H3N8) | 8/24/92 | 56.25871 | -117.80523 |
| A/pintail/Alberta/232/1992 (H3N8) | 8/24/92 | 56.25871 | -117.80523 |
| A/mallard/Alberta/54/1993 (H3N9) | 8/17/93 | 50.3 | -113.4 |
| A/mallard/Alberta/72/1993 (H3N8) | 8/17/93 | 50.3 | -113.4 |
| A/pintail/Alberta/129/1993 (H10N7) | 8/19/93 | 50.44 | -111.91 |
| A/mallard/Alberta/12/1993 (mixed) | 8/20/93 | 50.65 | -114.28 |
| A/mallard/Alberta/192/1993 (H3N8) | 8/22/93 | 55.87506 | -116.75127 |
| A/ruddy turnstone/Delaware Bay/78/1994 (mixed) | 5/22/94 | 39.11833 | -74.89105 |
| A/ruddy turnstone/Delaware Bay/81/1994 (H3N2) | 5/22/94 | 39.11833 | -74.89105 |
| A/ruddy turnstone/Delaware Bay/86/1994 (H3N8) | 5/22/94 | 39.11833 | -74.89105 |
| A/ruddy turnstone/Delaware Bay/123/1994 (H1N1) | 5/22/94 | 39.11833 | -74.89105 |
| A/ruddy turnstone/Delaware Bay/125/1994 (H3N8) | 5/22/94 | 39.11833 | -74.89105 |
| A/ruddy turnstone/Delaware Bay/143/1994 (mixed) | 5/22/94 | 39.11833 | -74.89105 |
| A/ruddy turnstone/Delaware Bay/144/1994 (mixed) | 5/22/94 | 39.11833 | -74.89105 |
| A/ruddy turnstone/Delaware Bay/158/1994 (H3N8) | 5/22/94 | 39.11833 | -74.89105 |
| A/ruddy turnstone/Delaware Bay/167/1994 (H3N8) | 5/22/94 | 39.11833 | -74.89105 |
| A/ruddy turnstone/Delaware Bay/168/1994 (mixed) | 5/22/94 | 39.11833 | -74.89105 |
| A/ruddy turnstone/Delaware Bay/180/1994 (H3N8) | 5/22/94 | 39.11833 | -74.89105 |
| A/shorebird/Delaware Bay/3/1994 (H3N2) | 5/22/94 | 39.047 | -74.92 |
| A/shorebird/Delaware Bay/28/1994 (H3N8) | 5/22/94 | 39.047 | -74.92 |
| A/shorebird/Delaware Bay/64/1994 (H3N2) | 5/22/94 | 39.047 | -74.92 |
| A/ruddy turnstone/Delaware Bay/87/1994 (H3N8) | 5/22/94 | 39.11833 | -74.89105 |
| A/ruddy turnstone/Delaware Bay/88/1994 (H3N8) | 5/22/94 | 39.11833 | -74.89105 |
| A/ruddy turnstone/Delaware Bay/118/1994 (H3N8) | 5/22/94 | 39.11833 | -74.89105 |
| A/ruddy turnstone/Delaware Bay/170/1994 (H3N2) | 5/22/94 | 39.11833 | -74.89105 |
| A/ruddy turnstone/Delaware Bay/176/1994 (H3N8) | 5/22/94 | 39.11833 | -74.89105 |
| A/ruddy turnstone/Delaware Bay/184/1994 (mixed) | 5/22/94 | 39.11833 | -74.89105 |
| A/ruddy turnstone/Delaware Bay/185/1994 (mixed) | 5/22/94 | 39.11833 | -74.89105 |
| A/ruddy turnstone/Delaware Bay/189/1994 (H3N8) | 5/22/94 | 39.11833 | -74.89105 |
| A/ruddy turnstone/Delaware Bay/203/1994 (H3N3) | 5/22/94 | 39.11833 | -74.89105 |
| A/shorebird/Delaware Bay/207/1994 (H3N8) | 5/23/94 | 39.11833 | -74.89105 |
| A/red knot/Delaware Bay/227/1994 (mixed) | 5/23/94 | 39.11833 | -74.89105 |
| A/shorebird/Delaware Bay/286/1994 (H3N8) | 5/23/94 | 39.11833 | -74.89105 |
| A/shorebird/Delaware Bay/206/1994 (H3N8) | 5/23/94 | 39.11833 | -74.89105 |
| A/ruddy turnstone/Delaware Bay/209/1994 (mixed) | 5/23/94 | 39.11833 | -74.89105 |
| A/red knot/Delaware Bay/218/1994 (H3N8) | 5/23/94 | 39.11833 | -74.89105 |
| A/red knot/Delaware Bay/236/1994 (H3N8) | 5/23/94 | 39.11833 | -74.89105 |
| A/red knot/Delaware Bay/252/1994 (H3N2) | 5/23/94 | 39.11833 | -74.89105 |
| A/shorebird/Delaware Bay/274/1994 (H7N5) | 5/23/94 | 39.11833 | -74.89105 |
| A/shorebird/Delaware Bay/275/1994 (mixed) | 5/23/94 | 39.11833 | -74.89105 |
| A/shorebird/Delaware Bay/277/1994 (H3N8) | 5/23/94 | 39.11833 | -74.89105 |
| A/mallard/Alberta/31/1994 (H3N8) | 9/8/94 | 56.22059 | -118.60448 |
| A/mallard/Alberta/72/1994 (H3N6) | 9/8/94 | 56.25871 | -117.80523 |
| A/mallard/Alberta/85/1994 (H3N8) | 12/8/94 | 50.3 | -113.4 |
| A/mallard/Alberta/142/1994 (H3N6) | 8/13/94 | 50.3 | -113.4 |
| A/mallard/Alberta/193/1994 (H3N8) | 8/14/94 | 50.46 | -111.74 |
| A/mallard/Alberta/43/1995 (H4N6) | 5/8/95 | 52.48843 | -112.93053 |
| A/mallard/Alberta/49/1995 (mixed) | 5/8/95 | 52.48843 | -112.93053 |
| A/pintail/Alberta/114/1995 (H3N8) | 7/8/95 | 56.22059 | -118.60448 |
| A/lesser scaup/Alberta/206/1995 (H3N8) | 12/8/95 | 52.48843 | -112.93053 |
| A/shorebird/Delaware Bay/31/1996 (H9N7) | 5/14/96 | 39.1875 | -74.95056 |
| A/ruddy turnstone/Delaware Bay/135/1996 (H7N3) | 5/15/96 | 39.11833 | -74.89105 |
| A/ruddy turnstone/Delaware Bay/215/1996 (mixed) | 5/15/96 | 39.11833 | -74.89105 |
| A/shorebird/Delaware Bay/260/1996 (H9N9) | 5/15/96 | 39.11833 | -74.89105 |
| A/pintail/Alberta/275/1996 (H4N6) | 8/26/96 | 56.22059 | -118.60448 |
| A/mallard/Alberta/296/1996 (H4N6) | 8/27/96 | 56.16094 | -118.51514 |
| A/shorebird/Delaware Bay/111/1997 (H2N1) | 5/14/97 | 39.1875 | -74.95056 |
| A/blue-winged teal/Alberta/16/1997 (H2N9) | 7/29/97 | 55.87506 | -116.75127 |
| A/mallard/Alberta/114/1997 (H3N8) | 5/8/97 | 50.3 | -113.4 |
| A/mallard/Alberta/117/1997 (H3N8) | 5/8/97 | 50.3 | -113.4 |
| A/shorebird/Delaware Bay/11/1998 (H3N8) | 5/18/98 | 39.1875 | -74.95056 |
| A/ruddy turnstone/Delaware Bay/142/1998 (H2N8) | 5/18/98 | 39.11833 | -74.89105 |
| A/shorebird/Delaware Bay/207/1998 (H3N8) | 5/19/98 | 39.11833 | -74.89105 |
| A/shorebird/Delaware Bay/210/1998 (H3N8) | 5/19/98 | 39.11833 | -74.89105 |
| A/shorebird/Delaware Bay/239/1998 (H3N8) | 5/19/98 | 39.11833 | -74.89105 |
| A/ruddy turnstone/Delaware Bay/250/1998 (H3N8) | 5/19/98 | 39.11833 | -74.89105 |
| A/ruddy turnstone/Delaware Bay/267/1998 (H3N8) | 5/19/98 | 39.11833 | -74.89105 |
| A/shorebird/Delaware Bay/237/1998 (H3N8) | 5/19/98 | 39.11833 | -74.89105 |
| A/mallard/Alberta/167/1998 (H3N8) | 7/8/98 | 55.87506 | -116.75127 |
| A/mallard/Alberta/169/1998 (H3N8) | 7/8/98 | 55.87506 | -116.75127 |
| A/mallard/Alberta/205/1998 (H2N3) | 11/8/98 | 56.16094 | -118.51514 |
| A/mallard/Alberta/211/1998 (H1N1) | 11/8/98 | 56.16094 | -118.51514 |
| A/mallard/Alberta/242/1998 (H3N8) | 11/8/98 | 56.22059 | -118.60448 |
| A/sanderling/Delaware Bay/65/1999 (mixed) | 5/23/99 | 39.0181 | -74.9473 |
| A/sanderling/Delaware Bay/70/1999 (H3N8) | 5/23/99 | 39.0181 | -74.9473 |
| A/shorebird/Delaware Bay/74/1999 (H3N2) | 5/24/99 | 39.10889 | -74.89444 |
| A/shorebird/Delaware Bay/88/1999 (H3N2) | 5/24/99 | 39.10889 | -74.89444 |
| A/shorebird/Delaware Bay/106/1999 (H3N2) | 5/24/99 | 39.10889 | -74.89444 |
| A/shorebird/Delaware Bay/110/1999 (H3N8) | 5/24/99 | 39.10889 | -74.89444 |
| A/shorebird/Delaware Bay/120/1999 (H3N2) | 5/24/99 | 39.10889 | -74.89444 |
| A/shorebird/Delaware Bay/122/1999 (H3N8) | 5/24/99 | 39.10889 | -74.89444 |
| A/shorebird/Delaware Bay/123/1999 (H3N2) | 5/24/99 | 39.10889 | -74.89444 |
| A/ruddy turnstone/Delaware Bay/136/1999 (H3N2) | 5/24/99 | 39.10889 | -74.89444 |
| A/ruddy turnstone/Delaware Bay/142/1999 (H3N2) | 5/24/99 | 39.10889 | -74.89444 |
| A/ruddy turnstone/Delaware Bay/144/1999 (mixed) | 5/24/99 | 39.10889 | -74.89444 |
| A/ruddy turnstone/Delaware Bay/147/1999 (mixed) | 5/24/99 | 39.10889 | -74.89444 |
| A/ruddy turnstone/Delaware Bay/150/1999 (H3N8) | 5/24/99 | 39.10889 | -74.89444 |
| A/shorebird/Delaware Bay/159/1999 (H3N8) | 5/24/99 | 39.10889 | -74.89444 |
| A/shorebird/Delaware Bay/189/1999 (H3N2) | 5/24/99 | 39.10889 | -74.89444 |
| A/shorebird/Delaware Bay/195/1999 (H3N2) | 5/24/99 | 39.10889 | -74.89444 |
| A/shorebird/Delaware Bay/198/1999 (mixed) | 5/24/99 | 39.10889 | -74.89444 |
| A/shorebird/Delaware Bay/200/1999 (H3N2) | 5/24/99 | 39.10889 | -74.89444 |
| A/shorebird/Delaware Bay/204/1999 (mixed) | 5/24/99 | 39.10889 | -74.89444 |
| A/shorebird/Delaware Bay/205/1999 (mixed) | 5/24/99 | 39.10889 | -74.89444 |
| A/shorebird/Delaware Bay/206/1999 (H3N2) | 5/24/99 | 39.10889 | -74.89444 |
| A/shorebird/Delaware Bay/208/1999 (H3N2) | 5/24/99 | 39.10889 | -74.89444 |
| A/shorebird/Delaware Bay/210/1999 (H3N2) | 5/24/99 | 39.10889 | -74.89444 |
| A/shorebird/Delaware Bay/212/1999 (H3N2) | 5/24/99 | 39.10889 | -74.89444 |
| A/shorebird/Delaware Bay/214/1999 (H3N2) | 5/24/99 | 39.10889 | -74.89444 |
| A/shorebird/Delaware Bay/215/1999 (H3N2) | 5/24/99 | 39.10889 | -74.89444 |
| A/shorebird/Delaware Bay/267/1999 (mixed) | 5/24/99 | 39.10889 | -74.89444 |
| A/shorebird/Delaware Bay/268/1999 (H3N8) | 5/24/99 | 39.10889 | -74.89444 |
| A/shorebird/Delaware Bay/270/1999 (H3N8) | 5/24/99 | 39.10889 | -74.89444 |
| A/shorebird/Delaware Bay/272/1999 (H3N8) | 5/24/99 | 39.10889 | -74.89444 |
| A/shorebird/Delaware Bay/280/1999 (H3N2) | 5/24/99 | 39.10889 | -74.89444 |
| A/shorebird/Delaware Bay/282/1999 (H3N8) | 5/24/99 | 39.10889 | -74.89444 |
| A/shorebird/Delaware Bay/287/1999 (H6N2) | 5/24/99 | 39.10889 | -74.89444 |
| A/ruddy turnstone/Delaware Bay/244/1999 (H3N8) | 5/24/99 | 39.21825 | -74.96042 |
| A/ruddy turnstone/Delaware Bay/261/1999 (H9N7) | 5/24/99 | 39.21825 | -74.96042 |
| A/pintail/Alberta/37/1999 (H3N8) | 7/31/99 | 55.87506 | -116.75127 |
| A/mallard/Alberta/199/1999 (H3N6) | 7/8/99 | 50.65 | -114.28 |
| A/pintail/Alberta/207/1999 (mixed) | 11/8/99 | 52.3389 | -116.8572 |
| A/mallard/Alberta/235/1999 (H4N6) | 11/8/99 | 52.48843 | -112.93053 |
| A/shorebird/Delaware Bay/277/2000 (H9N7) | 5/15/00 | 39.05 | -74.928 |
| A/mallard/Alberta/127/2000 (mixed) | 8/15/00 | 56.22059 | -118.60448 |
| A/shorebird/Delaware Bay/77/2001 (H6N2) | 5/17/01 | 39.10556 | -74.89611 |
| A/shorebird/Delaware Bay/113/2001 (mixed) | 5/17/01 | 39.10556 | -74.89611 |
| A/mallard/Alberta/22/2001 (H7N3) | 7/26/01 | 53.41684 | -112.9687 |
| A/mallard/Alberta/27/2001 (H7N3) | 7/26/01 | 53.41684 | -112.9687 |
| A/mallard/Alberta/35/2001 (H4N6) | 7/28/01 | 53.41684 | -112.9687 |
| A/mallard/Alberta/160/2001 (H3N8) | 6/8/01 | 56.2333 | -117.9667 |
| A/mallard/Alberta/38/2001 (H4N6) | 7/8/01 | 53.41684 | -112.9687 |
| A/shorebird/Delaware Bay/18/2002 (H1N9) | 5/20/02 | 39.11833 | -74.89105 |
| A/shorebird/Delaware Bay/53/2002 (H7N3) | 5/20/02 | 39.11833 | -74.89105 |
| A/shorebird/Delaware Bay/133/2002 (H9N1) | 5/21/02 | 39.10889 | -74.89444 |
| A/laughing gull/Delaware Bay/5/2003 (H9N1) | 5/19/03 | 39.11833 | -74.89105 |
| A/shorebird/Delaware Bay/73/2003 (H9N2) | 5/19/03 | 39.10556 | -74.89611 |
| A/shorebird/Delaware Bay/127/2003 (H9N2) | 5/19/03 | 39.10556 | -74.89611 |
| A/shorebird/Delaware Bay/246/2003 (H9N5) | 5/20/03 | 39.23106 | -75.16873 |
| A/shorebird/Delaware Bay/283/2003 (H9N1) | 5/20/03 | 39.23106 | -75.16873 |
| A/pintail/Alberta/166/2003 (H3N6) | 3/8/03 | 52.48843 | -112.93053 |
| A/mallard/Alberta/233/2003 (H4N6) | 8/8/03 | 56.16094 | -118.51514 |
| A/mallard/Alberta/237/2003 (H4N6) | 8/8/03 | 56.22059 | -118.60448 |
| A/mallard/Alberta/254/2003 (H3N3) | 8/8/03 | 50.3 | -113.4 |
| A/redhead/Alberta/192/2002 (H3N6) | 8/16/03 | 55.87506 | -116.75127 |
| A/mallard/Alberta/192/2004 (H3N1) | 8/14/04 | 52.48843 | -112.93053 |
| A/mallard/Alberta/226/2004 (H4N6) | 8/15/04 | 56.25871 | -117.80523 |
| A/mallard/Alberta/241/2004 (H3N8) | 8/15/04 | 56.25871 | -117.80523 |
| A/mallard/Alberta/251/2004 (H4N6) | 8/15/04 | 56.25871 | -117.80523 |
| A/shorebird/Delaware Bay/117/2005 (mixed) | 5/19/05 | 39.00452 | -75.00385 |
| A/shorebird/Delaware Bay/140/2005 (H3N6) | 5/19/05 | 39.00452 | -75.00385 |
| A/shorebird/Delaware Bay/148/2005 (H3N6) | 5/19/05 | 39.00452 | -75.00385 |
| A/shorebird/Delaware Bay/153/2005 (H3N6) | 5/19/05 | 39.00452 | -75.00385 |
| A/shorebird/Delaware Bay/161/2005 (H3N6) | 5/19/05 | 39.00452 | -75.00385 |
| A/shorebird/Delaware Bay/167/2005 (H3N8) | 5/19/05 | 39.00452 | -75.00385 |
| A/shorebird/Delaware Bay/170/2005 (H3N6) | 5/19/05 | 39.00452 | -75.00385 |
| A/shorebird/Delaware Bay/179/2005 (H3N6) | 5/19/05 | 39.00452 | -75.00385 |
| A/shorebird/Delaware Bay/201/2005 (mixed) | 5/19/05 | 39.00452 | -75.00385 |
| A/shorebird/Delaware Bay/289/2005 (H3N6) | 5/19/05 | 39.29447 | -75.16457 |
| A/shorebird/Delaware Bay/274/2005 (H3N8) | 5/19/05 | 39.29447 | -75.16457 |
| A/shorebird/Delaware Bay/435/2005 (H3N8) | 5/21/05 | 39.05 | -74.928 |
| A/shorebird/Delaware Bay/450/2005 (H3N8) | 5/21/05 | 39.05 | -74.928 |
| A/shorebird/Delaware Bay/477/2005 (H3N8) | 5/21/05 | 39.05 | -74.928 |
| A/shorebird/Delaware Bay/443/2005 (mixed) | 5/21/05 | 39.05 | -74.928 |
| A/shorebird/Delaware Bay/518/2005 (H3N6) | 5/21/05 | 39.29447 | -75.16457 |
| A/shorebird/Delaware Bay/519/2005 (H3N6) | 5/21/05 | 39.29447 | -75.16457 |
| A/mallard/Alberta/66/2005 (H4N6) | 7/27/05 | 56.22059 | -118.60448 |
| A/blue-winged teal/Alberta/222/2005 (H3N8) | 10/8/05 | 52.48843 | -112.93053 |
| A/laughing gull/Delaware Bay/42/2006 (H7N3) | 5/22/06 | 39.0181 | -74.9473 |
| A/ruddy turnstone/Delaware Bay/281/2006 (H7N3) | 5/22/06 | 39.11833 | -74.89105 |
| A/ruddy turnstone/Delaware Bay/282/2006 (H7N3) | 5/22/06 | 39.11833 | -74.89105 |
| A/sanderling/Delaware Bay/449/2006 (H9N2) | 5/23/06 | 39.00452 | -75.00385 |
| A/shorebird/Delaware Bay/552/2006 (H7N3) | 5/23/06 | 39.11833 | -74.89105 |
| A/shorebird/Delaware Bay/513/2006 (H7N3) | 5/23/06 | 39.21825 | -74.96042 |
| A/shorebird/Delaware Bay/332/2006 (H7N3) | 5/23/06 | 39.29447 | -75.16457 |
| A/mallard/Alberta/190/2006 (mixed) | 4/8/06 | 55.87506 | -116.75127 |
| A/mallard/Alberta/297/2006 (H4N6) | 9/8/06 | 56.25871 | -117.80523 |
| A/mallard/Alberta/212/2006 (H3N8) | 9/8/06 | 56.16094 | -118.51514 |
| A/mallard/Alberta/214/2006 (H3N8) | 9/8/06 | 56.16094 | -118.51514 |
| A/mallard/Alberta/219/2006 (H3N8) | 9/8/06 | 56.16094 | -118.51514 |
| A/mallard/Alberta/220/2006 (mixed) | 9/8/06 | 56.16094 | -118.51514 |
| A/mallard/Alberta/221/2006 (H12N6) | 9/8/06 | 56.16094 | -118.51514 |
| A/mallard/Alberta/224/2006 (H12N5) | 9/8/06 | 56.16094 | -118.51514 |
| A/mallard/Alberta/225/2006 (H3N8) | 9/8/06 | 56.16094 | -118.51514 |
| A/mallard/Alberta/228/2006 (H3N8) | 9/8/06 | 56.16094 | -118.51514 |
| A/mallard/Alberta/238/2006 (H3N8) | 9/8/06 | 56.16094 | -118.51514 |
| A/mallard/Alberta/243/2006 (H7N3) | 9/8/06 | 56.16094 | -118.51514 |
| A/mallard/Alberta/246/2006 (H4N6) | 9/8/06 | 56.16094 | -118.51514 |
| A/mallard/Alberta/254/2006 (H4N6) | 9/8/06 | 56.16094 | -118.51514 |
| A/mallard/Alberta/256/2006 (mixed) | 9/8/06 | 56.16094 | -118.51514 |
| A/mallard/Alberta/258/2006 (H4N6) | 9/8/06 | 56.16094 | -118.51514 |
| A/mallard/Alberta/274/2006 (H3N8) | 9/8/06 | 56.22059 | -118.60448 |
| A/mallard/Alberta/279/2006 (H3N8) | 9/8/06 | 53.555978 | -116.343541 |
| A/ruddy turnstone/Delaware Bay/124/2007 (H7N3) | 5/22/07 | 39.114 | -74.8918 |
| A/mallard/Alberta/234/2007 (H12N5) | 3/8/07 | 52.48843 | -112.93053 |
| A/mallard/Alberta/284/2007 (mixed) | 4/8/07 | 52.48843 | -112.93053 |
| A/northern pintail/Alberta/265/2007 (H4N6) | 4/8/07 | 52.48843 | -112.93053 |
| A/mallard/Alberta/27/2007 (H3N8) | 8/18/07 | 55.87506 | -116.75127 |
| A/mallard/Alberta/28/2007 (H3N8) | 8/18/07 | 55.87506 | -116.75127 |
| A/mallard/Alberta/34/2007 (H3N8) | 8/18/07 | 55.87506 | -116.75127 |
| A/duck/Alberta/126/2007 (H3N8) | 8/18/07 | 56.16094 | -118.51514 |
| A/mallard/Alberta/106/2007 (mixed) | 8/22/07 | 56.16094 | -118.51514 |
| A/mallard/Alberta/107/2007 (H3N8) | 8/22/07 | 56.16094 | -118.51514 |
| A/mallard/Alberta/108/2007 (mixed) | 8/22/07 | 56.16094 | -118.51514 |
| A/mallard/Alberta/112/2007 (mixed) | 8/22/07 | 56.16094 | -118.51514 |
| A/mallard/Alberta/114/2007 (H4N6) | 8/22/07 | 56.16094 | -118.51514 |
| A/mallard/Alberta/115/2007 (mixed) | 8/22/07 | 56.16094 | -118.51514 |
| A/mallard/Alberta/116/2007 (H3N8) | 8/22/07 | 56.16094 | -118.51514 |
| A/mallard/Alberta/122/2007 (H3N8) | 8/22/07 | 56.16094 | -118.51514 |
| A/mallard/Alberta/130/2007 (H3N8) | 8/22/07 | 56.16094 | -118.51514 |
| A/mallard/Alberta/134/2007 (H3N8) | 8/22/07 | 56.16094 | -118.51514 |
| A/mallard/Alberta/137/2007 (H3N8) | 8/22/07 | 56.16094 | -118.51514 |
| A/mallard/Alberta/145/2007 (H4N6) | 8/22/07 | 56.16094 | -118.51514 |
| A/mallard/Alberta/156/2007 (mixed) | 8/22/07 | 56.16094 | -118.51514 |
| A/mallard/Alberta/160/2007 (H4N6) | 8/22/07 | 56.16094 | -118.51514 |
| A/redhead/Alberta/458/2007 (H3N8) | 8/28/07 | 52.48843 | -112.93053 |
| A/mallard/Alberta/312/2007 (H3N8) | 8/28/07 | 52.48843 | -112.93053 |
| A/mallard/Alberta/318/2007 (H3N8) | 8/28/07 | 52.48843 | -112.93053 |
| A/mallard/Alberta/330/2007 (H4N6) | 8/28/07 | 52.48843 | -112.93053 |
| A/redhead/Alberta/337/2007 (mixed) | 8/28/07 | 52.48843 | -112.93053 |
| A/blue-winged teal/Alberta/340/2007 (mixed) | 8/28/07 | 52.48843 | -112.93053 |
| A/blue-winged teal/Alberta/346/2007 (H4N3) | 8/28/07 | 52.48843 | -112.93053 |
| A/blue-winged teal/Alberta/366/2007 (H3N8) | 8/28/07 | 52.48843 | -112.93053 |
| A/blue-winged teal/Alberta/376/2007 (H3N6) | 8/28/07 | 52.48843 | -112.93053 |
| A/blue-winged teal/Alberta/378/2007 (mixed) | 8/28/07 | 52.48843 | -112.93053 |
| A/blue-winged teal/Alberta/380/2007 (mixed) | 8/28/07 | 52.48843 | -112.93053 |
| A/blue-winged teal/Alberta/387/2007 (H3N8) | 8/28/07 | 52.48843 | -112.93053 |
| A/bufflehead/Alberta/399/2007 (mixed) | 8/28/07 | 52.48843 | -112.93053 |
| A/bufflehead/Alberta/400/2007 (H3N8) | 8/28/07 | 52.48843 | -112.93053 |
| A/shorebird/Delaware Bay/485/2008 (H3N2) | 5/17/08 | 39.00181 | -74.014878 |
| A/mallard/Alberta/121/2008 (H4N6) | 6/8/08 | 56.25871 | -117.80523 |
| A/mallard/Alberta/270/2008 (H4N6) | 7/8/08 | 56.16094 | -118.51514 |
| A/mallard/Alberta/507/2008 (H3N8) | 8/8/08 | 56.16094 | -118.51514 |
| A/mallard/Alberta/527/2008 (H3N8) | 8/8/08 | 56.16094 | -118.51514 |
| A/shorebird/Delaware Bay/275/2009 (mixed) | 5/20/09 | 39.10872 | -74.89317 |
| A/northern pintail/Alberta/8/2009 (H3N8) | 8/19/09 | 52.35 | -112.9168 |
| A/mallard/Alberta/421/2009 (H2N3) | 8/22/09 | 56.160944 | -118.515139 |
| A/mallard/Alberta/417/2009 (H2N3) | 8/22/09 | 56.160944 | -118.515139 |
| A/mallard/Alberta/551/2009 (H3N8) | 8/23/09 | 56.258714 | -117.805228 |
