# Supplementary material for: Influenza A Virus Migration and Persistence in North American Wild Birds
Source: PLoS Pathog. 2013 Aug 29;9(8):e1003570. doi: 10.1371/journal.ppat.1003570 (PMC3757048; doi:10.1371/journal.ppat.1003570)
Supplement: Table S4 — Number of taxa included per protein coding region to estimate average migration dynamics between discrete regions. (DOC) [file ppat.1003570.s021.doc]

**Table S4:** Number of taxa included per protein coding region to estimate average migration dynamics between discrete regions.

|  | Number of sequences per gene | | | | |
| --- | --- | --- | --- | --- | --- |
| Discrete Location | PA | PB1 | PB2 | NP | M |
| Alaska | 251 | 247 | 251 | 252 | 254 |
| NWAlberta | 71 | 71 | 69 | 73 | 68 |
| BritishColumbia | 46 | 46 | 46 | 46 | 46 |
| SWAlberta | 10 | 10 | 10 | 10 | 10 |
| SEAlberta | 139 | 141 | 142 | 139 | 135 |
| Saskatchewan* | 2 | 2 | 2 | 2 | 2 |
| Manitoba* | 4 | 4 | 4 | 4 | 4 |
| Oregon | 13 | 13 | 13 | 13 | 13 |
| California | 238 | 236 | 238 | 238 | 238 |
| SouthDakota | 4 | 0 | 4 | 4 | 4 |
| Minnesota | 56 | 56 | 57 | 56 | 56 |
| Quebec-NY | 45 | 45 | 45 | 45 | 45 |
| NewBrunswick | 9 | 9 | 9 | 9 | 9 |
| Iowa-Missouri | 11 | 11 | 11 | 11 | 11 |
| Wisconsin-Illinois | 82 | 82 | 82 | 82 | 82 |
| Ontario-Ohio | 84 | 84 | 84 | 84 | 84 |
| Tennessee* | 1 | 1 | 1 | 1 | 1 |
| DELB-NJ-Maryland | 238 | 219 | 231 | 234 | 226 |
| Texas | 19 | 19 | 19 | 19 | 19 |
| Mississippi-Louisiana | 22 | 22 | 22 | 22 | 22 |
| Total | 1345 | 1318 | 1340 | 1344 | 1329 |

*All isolates sampled before 1998 and locations with fewer than 5 isolates were grouped as location state “Other.” These were included to maintain phylogenetic structure for estimation of migration patterns between all other states.
