# Supplementary material for: Influenza A Virus Migration and Persistence in North American Wild Birds
Source: PLoS Pathog. 2013 Aug 29;9(8):e1003570. doi: 10.1371/journal.ppat.1003570 (PMC3757048; doi:10.1371/journal.ppat.1003570)
Supplement: Text S1 — Supplementary information describing flyways and bird behavior. (DOC) [file ppat.1003570.s022.doc]

**Influenza A virus migration and persistence in North American wild birds**

Justin Bahl^1,2*^, Scott Krauss^3*^, Denise Kühnert^4,5^, Mathieu Fourment^1^, Garnet Raven^6^, S. Paul Pryor^6^, Lawrence J. Niles^7^, Angela Danner^3^, David Walker^3^, Ian H. Mendenhall^1^, Yvonne C.F. Su^1^, Vivien G. Dugan^8,9^, Rebecca A. Halpin^8^, Timothy B. Stockwell^8^, Richard J. Webby^3^, David E. Wentworth^8^, Alexei J. Drummond^4,5^, Gavin J.D. Smith^1,10‡^, Robert G. Webster^3‡^

^1^Laboratory of Virus Evolution, Program in Emerging Infectious Diseases, Duke-NUS Graduate Medical School, 8 College Rd, Singapore, 169857; ^2^Center for Infectious Diseases, The University of Texas School of Public Health, Houston USA, 77030; ^3^Department of Infectious Diseases, St. Jude Children’s Research Hospital, Memphis, TN 38105-3678; ^4^Department of Computer Science, University of Auckland, Private Bag 92019, Auckland, New Zealand; ^5^Allan Wilson Centre for Molecular Ecology and Evolution, University of Auckland, Auckland, New Zealand; ^6^Environment Canada, Canadian Wildlife Service, Edmonton, Alberta, Canada; ^7^Conserve Wildlife Foundation of New Jersey, 516 Farnsworth Avenue, Bordentown, NJ 08505 USA; ^8^J. Craig Venter Institute, 9704 Medical Center Drive, Rockville, Maryland 20850; ^9^Division of Microbiology and Infectious Diseases/NIAID/NIH/DHHS, Bethesda Maryland 20892;  ^10^Duke Global Health Institute, Duke University, Durham, North Carolina 27708;

* These authors contributed equally to this work

‡ To whom correspondence should be addressed:

[gavin.smith@duke-nus.edu.sg](mailto:gavin.smith@duke-nus.edu.sg) or [robert.webster@stjude.org](mailto:Robert.webster@stjude.org)

**1. Supplementary information describing flyways and bird behavior**

Migration is a necessary undertaking for a number of bird species. This is usually over large geographic distances resulting from differential availability of food across latitudinal gradients due to seasonal climate changes. It is estimated that over 50 billion birds migrate annually between breeding and non-breeding areas [1]. The US Fish and Wildlife list 1,006 migratory birds protected under the Migratory Bird Treaty Act [2]. Approximately two hundred of these are neotropical migrants [3]. Avian influenza virus has been detected in over 100 species of wild birds, though ducks (Order: *Anseriformes*), gulls and wading birds (Order: *Charadriiformes*) are the primary natural reservoirs [4].

Migratory flyways are broad geographical routes taken by birds that may follow specific topographical areas, such as the Mississippi River or the Rocky Mountain range. In North America, there are four flyways: the Atlantic, Mississippi, Central, and the Pacific. The Atlantic flyway supports birds from the Atlantic coast to the Allegheny Mountain range. The Mississippi flyway ranges from west of the Allegheny Mountains and merges with the Central flyway along the Nebraska-Missouri boarder. The Central flyway continues to the eastern edge of the Rocky Mountains. The union of these two flyways, from the Northwest Territories to the Mississippi delta, represents a corridor between the Rocky Mountains and Appalachian Mountains used by large numbers of migratory waterbirds. The distinction of the Mississippi and the Central flyways are largely administrative as birds regularly migrate between these flyways. Duck banding programs have shown thousands of birds breeding in the northern Central Flyway region will migrate through the Central and Mississippi Flyways to Chesapeake Bay in the Atlantic flyway [5]. The Rocky Mountains however, are a significant geographical barrier that demarcates the Pacific flyway from the Central flyway.

Administrative boundaries are defined primarily along state borders except the Pacific and Central flyways, which are separated by the Rocky Mountains. Biological boundaries attempt to define flyways based upon actual bird movement and banding records and typically contained within administrative flyways, however, these flyways oversimplify bird movement [6]. Using duck banding and recovery information from the USGS Patuxent Wildlife Research Center, Duck’s Unlimited Canada demonstrated that while these birds will use specific flyways, individuals regularly disperse and migrate into neighboring defined flyways [5].

Flyways are poorly defined in higher latitudinal gradients where birds from multiple flyways congregate. The prairie pothole region, which covers five Midwestern US states and three Canadian provinces, is one where waterfowl from different flyways converge to feed and breed. Duck populations vacillate annually based primarily on the availability of suitable habitat (number and quality of wetlands). In 2011 an estimated 45 million ducks used the prairie pothole region, a critical nesting and breeding region from Nebraska to northern Saskatchewan. This was 11% higher than 2010 counts and 35% higher than average historical counts (1955-2010) [7]. The major sampling sites in Alberta are nested within the prairie pothole region. Additionally, birds that summer in Mackenzie Delta in the Northwest Territories of Canada or in Alaska may head south along the Pacific coast, through Alberta and onto the Great Salt Lake, following the Mississippi River, or to the Atlantic coast [8].

Stopover site selection during migration is critical because of the short temporal scale. Such sites are often limited and therefore numerous species will congregate to take advantage of abundant resources [9]. These birds are often physiologically stressed from migration and may be more susceptible to infection with avian influenza virus [10]. The primary shorebird migratory stopover and breeding sites in North America are: the Copper River Delta in southern Alaska, Gray’s Harbor in Washington state, the prairie pothole region in the northern portions of the Central flyway, Cheyenne Bottoms in Kansas, Delaware Bay shores in New Jersey and Delaware, and Fundy Bay in Nova Scotia and New Brunswick. Delaware Bay supports over 500,000 shorebirds annually and is designated by The Western Hemisphere Shorebird Reserve Network as a site of hemispheric importance for the natural history and conservation for shorebirds [11].

***1.1 References***

1. Berthold P (2001) Bird migration: a general survey. Oxford ornithology series. Oxford: Oxford University Press, 266 p.
2. U.S. Fish & Wildlife Service. List of Migratory Birds. *Migratory Bird Program* Available; <http://www.fws.gov/migratorybirds/RegulationsPolicies/mbta/mbtandx.html> Accessed April 11, 2012
3. Smithsonian National Zoological Park. Neotropical Migratory Bird Basics. *Migratory Bird Center* (2012). Available;

<http://nationalzoo.si.edu/scbi/MigratoryBirds/Fact_Sheets/default.cfm?fxsht=9> Accessed June 25, 2012

1. Webster RG, Bean WJ, Gorman OT, Chambers TM, Kawaoka Y, Evolution and ecology of influenza A viruses. Microbiol. Rev. 56: 152-179 (1992).
2. Ducks Unlimited Canada. International Migration Maps. *Ducks Unlimited Canada*. Availabe; <http://www.ducks.ca/resource/general/wetland/migration/maps.html> Accessed June 25, 2012
3. United States Geological Survey, *Migration of Birds: Routes of Migration.* (Northern Prairie Wildlife Research Center Available; <http://www.npwrc.usgs.gov/resource/birds/migratio/routes.htm> Accessed June 25, 2012
4. U.S. Fish and Wildlife Service (2011) Waterfowl population status, 2011. Available: <http://www.fws.gov/migratorybirds/NewReportsPublications/PopulationStatus/Waterfowl/2011WaterfowlStatusReport.pdf>. Accessed 20 June 2012
5. Cooke WW, Bird Migration in the Mackenzie Valley. *The Auk*. **32**, 442-459 (1915).
6. Morrison RIG, Harrington BA (1979) Critical shorebird resources in James Bay and eastern North America. Transactions of the 44th North American Wildlife and Natural Resources Conference, Wildlife Management Institute, Washington, D.C.
7. Reperant LA, van de Bildt MWG, van Amerongen G, Buehler DM, Osterhaus ADME, et al. (2011) Highly Pathogenic Avian Influenza Virus H5N1 Infection in a Long-Distance Migrant Shorebird under Migratory and Non-Migratory States. PLoS ONE 6: e27814. doi:10.1371/journal.pone.0027814
8. Western Hemisphere Shorebird Reserve Network. Delaware Bay. Western Hemisphere Shorebird Reserve Network Site Profile. Available; <http://www.whsrn.org/site-profile/delaware-bay> Accessed June 25, 2012
9. Squires RB, Noronha J, Hunt V, Garcia-Sastre A, Macken C, et al. (2012) [Influenza research database: an integrated bioinformatics resource for influenza research and surveillance](http://www.ncbi.nlm.nih.gov/pubmed/22260278). Influenza Other Respi Viruses DOI: 10.1111/j.1750-2659.2011.00331.x.
10. Delany S, Scott D (2006) Waterbird Population Estimates, Fourth Edition. Wageningen, Netherlands. Wetlands International.
11. U.S. Fish and Wildlife Service (2003) Delaware Bay shorebird-horseshoe crab assessment report and peer review. U.S. Fish and Wildlife Service Migratory Bird Publication R9-03/02. 2003 Arlington, VA. 99 p.
12. Niles LJ, Sitters HP, Dey AD, Arce N, Atkinson PW, et al. (2010) Update to the status of the red knot *Calidris canutus* in the western hemisphere. Available: <http://www.nj.gov/dep/fgw/ensp/pdf/redknot_update4-10.pdf>. Accessed 20 June 2012
13. Morrison RIG, McCaffery BJ, Gill RE, Skagen SK, Jones SL, et al. (2006) Population estimates of North American shorebirds, 2006. Wader Study Group Bull. 111: 67-85.
